# Comparative genomics and transcriptomics of 4 *Paragonimus* species provide insights into lung fluke parasitism and pathogenesis

**DOI:** 10.1093/gigascience/giaa073

**Published:** 2020-07-20

**Authors:** Bruce A Rosa, Young-Jun Choi, Samantha N McNulty, Hyeim Jung, John Martin, Takeshi Agatsuma, Hiromu Sugiyama, Thanh Hoa Le, Pham Ngoc Doanh, Wanchai Maleewong, David Blair, Paul J Brindley, Peter U Fischer, Makedonka Mitreva

**Affiliations:** Department of Internal Medicine, Washington University School of Medicine, 660 S Euclid Ave, St. Louis, MO 63110, USA; Department of Internal Medicine, Washington University School of Medicine, 660 S Euclid Ave, St. Louis, MO 63110, USA; The McDonnell Genome Institute at Washington University, School of Medicine, 4444 Forest Park Ave, St. Louis, MO 63108, USA; Department of Internal Medicine, Washington University School of Medicine, 660 S Euclid Ave, St. Louis, MO 63110, USA; Department of Internal Medicine, Washington University School of Medicine, 660 S Euclid Ave, St. Louis, MO 63110, USA; Department of Environmental Health Sciences, Kochi Medical School, Kohasu, Oko-cho 185-1, Nankoku, Kochi, 783-8505, Japan; Laboratory of Helminthology, Department of Parasitology, National Institute of Infectious Diseases, 1-23-1 Toyama, Shinjuku-ku, Tokyo 162-8640, Japan; Department of Immunology, Institute of Biotechnology, Vietnam Academy of Science and Technology, 18 Hoang Quoc Viet, Cay Giay, Ha Noi 10307, Vietnam; Institute of Ecology and Biological Resources, Vietnam Academy of Science and Technology, 18 Hoang Quoc Viet, Cay Giay, Ha Noi 10307, Vietnam; Graduate University of Science and Technology, Vietnam Academy of Science and Technology, 18 Hoang Quoc Viet, Cay Giay, Ha Noi 10307, Vietnam; Research and Diagnostic Center for Emerging Infectious Diseases, Khon Kaen University, 123 Moo 16 Mittraphap Rd., Nai-Muang, Muang District, Khon Kaen 40002, Thailand; Department of Parasitology, Faculty of Medicine, Khon Kaen University, 123 Moo 16 Mittraphap Rd., Nai-Muang, Muang District, Khon Kaen 40002, Thailand; College of Marine and Environmental Sciences, James Cook University, 1 James Cook Drive, Townsville, Queensland 4811, Australia; Departments of Microbiology, Immunology and Tropical Medicine, and Research Center for Neglected Diseases of Poverty, and Pathology School of Medicine & Health Sciences, George Washington University, Ross Hall 2300 Eye Street, NW, Washington, DC 20037, USA; Department of Internal Medicine, Washington University School of Medicine, 660 S Euclid Ave, St. Louis, MO 63110, USA; Department of Internal Medicine, Washington University School of Medicine, 660 S Euclid Ave, St. Louis, MO 63110, USA; The McDonnell Genome Institute at Washington University, School of Medicine, 4444 Forest Park Ave, St. Louis, MO 63108, USA

**Keywords:** lung flukes, *Paragonimus*, genomics, transcriptomics, diagnostics, paragonimiasis, infectious disease, trematodes

## Abstract

**Background:**

*Paragonimus* spp. (lung flukes) are among the most injurious foodborne helminths, infecting ~23 million people and subjecting ~292 million to infection risk. Paragonimiasis is acquired from infected undercooked crustaceans and primarily affects the lungs but often causes lesions elsewhere including the brain. The disease is easily mistaken for tuberculosis owing to similar pulmonary symptoms, and accordingly, diagnostics are in demand.

**Results:**

We assembled, annotated, and compared draft genomes of 4 prevalent and distinct *Paragonimus* species: *Paragonimus miyazakii, Paragonimus westermani, Paragonimus kellicotti*, and *Paragonimus heterotremus*. Genomes ranged from 697 to 923 Mb, included 12,072–12,853 genes, and were 71.6–90.1% complete according to BUSCO. Orthologous group analysis spanning 21 species (lung, liver, and blood flukes, additional platyhelminths, and hosts) provided insights into lung fluke biology. We identified 256 lung fluke–specific and conserved orthologous groups with consistent transcriptional adult-stage *Paragonimus* expression profiles and enriched for iron acquisition, immune modulation, and other parasite functions. Previously identified *Paragonimus* diagnostic antigens were matched to genes, providing an opportunity to optimize and ensure pan-*Paragonimus* reactivity for diagnostic assays.

**Conclusions:**

This report provides advances in molecular understanding of *Paragonimus* and underpins future studies into the biology, evolution, and pathogenesis of *Paragonimus* and related foodborne flukes. We anticipate that these novel genomic and transcriptomic resources will be invaluable for future lung fluke research.

## Background

The trematode genus *Paragonimus*, the lung flukes, is among the most injurious taxon of food-borne helminths. Approximately 23 million people are infected with lung flukes [1], an estimated 292 million people are at risk, mainly in eastern Asia [2], and billions of people live in areas where *Paragonimus* infections of animals are endemic. The life-cycle of *Paragonimus* species involves freshwater snails, crustacean intermediate hosts, and mammals in Asia, parts of Africa, and the Americas [3]. Human paragonimiasis is acquired by consuming raw or undercooked shrimp and crabs containing the metacercaria, which is the infective stage. Although primarily affecting the lungs, lesions can occur at other sites, including the brain, and pulmonary paragonimiasis is frequently mistaken for tuberculosis owing to similar respiratory symptoms [4].

Pathogenesis ensues because of the migration of the newly invading juveniles from the gut to the lungs and through not-infrequent ectopic migration to the brain, reproductive organs, and subcutaneous sites at the extremities, and because of toxins and other mediators released by the parasites during the larval migration [4, 5]. The presence of the flukes in the lung causes hemorrhage, inflammation with leukocytic infiltration, and necrosis of lung parenchyma that gradually proceeds to the development of fibrotic encapsulation except for a fistula from the evolving lesion to the respiratory tract. Eggs of the lung fluke exit the encapsulated lesion through the fistula to reach the sputum and/or feces of the host, where they pass to the external environment, accomplishing transmission of the parasite [6]. There are signs and symptoms that allow characterization of acute and chronic stages of paragonimiasis. In pulmonary paragonimiasis, for example, the most noticeable clinical symptom of an infected individual is a chronic cough with gelatinous, rusty brown, pneumonia-like, blood-streaked sputum [6]. Heavy work commonly induces hemoptysis. Pneumothorax, empyema from secondary bacterial infection, and pleural effusion might also be presented. When symptoms include only a chronic cough, the disease may be misinterpreted as chronic bronchitis and bronchiectasis or bronchial asthma. Pulmonary paragonimiasis is frequently confused with pulmonary tuberculosis [4]. The symptoms of extra-pulmonary paragonimiasis vary depending on the location of the fluke, including cerebral [5] and abdominal paragonimiasis [6].

*Paragonimus* is a large genus that includes >50 nominal species [7]. Seven of these species or species complexes of *Paragonimus* are known to infect humans [3]. This is also an ancient genus, thought to have originated before the breakup of Gondwana [8], but possibly also dispersing as colonists from the original East Asian clade, based on the distribution of host species [9]. To improve our understanding of pathogens across this genus at the molecular level, we have assembled, annotated, and compared draft genomes of 4 of these, 3 from Asia (*Paragonimus westermani* from Japan, *Paragonimus herotremus, Paragonimus miyazakii*) and 1 from North America (*Paragonimus kellicotti*). Among them, *P. westermani* is the best-known species causing pulmonary paragonimiasis. This name has been applied to a genetically and geographically diverse complex of lung fluke populations differing widely in biological features including infectivity to humans [10]. The complex extends from India and Sri Lanka eastwards to Siberia, Korea, and Japan, and southwards into Vietnam, Indonesia, and the Philippines. However, human infections are reported primarily from China, Korea, Japan, and the Philippines. Until this study, an Indian member of the *P. westermani* complex was the only lung fluke species for which a genome sequence was available [11]. *Paragonimus heterotremus* is the most common cause of pulmonary paragonimiasis in southern China, Lao PDR, Vietnam, northeastern India, and Thailand [6, 7]. *Paragonimus miyazakii* is a member of the *Paragonimus skrjabini* complex, to which Blair and co-workers accorded subspecific status [12]. Flukes of this complex tend not to mature in humans but frequently cause ectopic disease at diverse sites, including the brain. In North America, infection with *P*. *kellicotti* is primarily a disease of native, crayfish-eating mammals including the otter and mink. The occasional human infections can be severe, and thoracic involvement is typical [13, 14].

These 4 species represent a broad sampling of the phylogenetic diversity of the genus. Most of the known diversity, as revealed by DNA sequences from portions of the mitochondrial genome and the nuclear ribosomal genes, resides in Asia [15]. Analysis of the ITS2 marker by Blair et al. [15] indicates that each of the species sequenced occupies a distinct clade within the phylogenetic tree.

In addition to a greater understanding of the genome contents of this group of foodborne trematodes, the findings presented here provide new information to assist development of diagnostic tools and recognition of potential drug targets. The data and findings facilitate evolutionary, zoogeographical, and phylogenetic investigation of the genus *Paragonimus* and its host-parasite relationships through the comparative analysis of gene content relative to other sequenced platyhelminth and host species, and to known *Paragonimus* diagnostic antigen targets.

## Data Description

Genomic sequence data were generated from DNA samples from 4 distinct *Paragonimus* species: 3 from Asia, *P. miyazakii* (Japan), *P. heterotremus* (LC strain, Vietnam), and*Paragonimus westermani* (Japan), and 1 from North America, *P. kellicotti* (Missouri, USA). Illumina DNA sequencing produced short overlapping fragments and long insert size (3 and 8 kb) whole-genome shotgun libraries for all 4 spcies. Genome coverage per species is presented in Table 1. Owing to the higher fragmentation rate of the *P. kellicotti* assembly, long-read Pacific Biosciences (PacBio) reads were generated and used for assembly improvement (Table 2). To estimate the genetic divergence between geographically diverse samples, we compared our East Asian *P. westermani* sample from Japan with the previously published *P. westermani* genome from India by retrieving the Inidian genome from the previous study [11]. To facilitate gene annotation in the newly generated assemblies and to provide transcriptomic data for analysis, adult-stage RNA sequencing (RNA-seq) samples were also retrieved from our previous reports for *P. westermani* [16] and *P. kellicotti* [17]. We also collected adult-stage RNA samples for Illumina RNA-seq from young adult and adult samples for *P. heterotremus*, along with stages from the liver, peritoneal cavity, lung (adult), and pleural cavity for *P. miyazakii*.

**Table 1:** *Paragonimus*spp. genome and RNA-seq data accessions

**Genome assemblies, annotations, and raw reads**			
Species	NCBI accession	Bioproject ID	Genome coverage (×)
*Paragonimus miyazakii*	JTDE00000000	PRJNA245325	162
*Paragonimus heterotremus*	LUCH00000000	PRJNA284523	81
*Paragonimus kellicotti*	LOND00000000	PRJNA179523	77 (43*)
*Paragonimus westermani*	JTDF00000000	PRJNA219632	152
**RNA-Seq dataset accessions**			
Species	NCBI accession	Bioproject ID	Body site or stage
*Paragonimus miyazakii*	SRX1100074	PRJNA245325	Pleural cavity
	SRX1100062	PRJNA245325	Lung
	SRX1037170	PRJNA245325	Peritoneal cavity
	SRX1037172	PRJNA245325	Peritoneal cavity
	SRX1037171	PRJNA245325	Liver
*Paragonimus heterotremus*	SRX3713099	PRJNA284523	Adult (technical rep 1)
	SRX3713100	PRJNA284523	Adult (technical rep 2)
	SRX3713101	PRJNA284523	Young adult
	SRX3713102	PRJNA284523	Young adult
*Paragonimus kellicotti*	SRX3718311	PRJNA179523	Adult
	SRX3718310	PRJNA179523	Adult
*Paragonimus westermani*	SRX1507710	PRJNA219632	Adult

*Pacific Biosciences dataset coverage.

**Table 2:** The draft genome of *Paragonimus*: assembly, size, and annotation characteristics

Statistic	*Paragonimus miyazakii*	*Paragonimus heterotremus*	*Paragonimus kellicotti*	*Paragonimus westermani* (Japan)	*Paragonimus westermani* (India)
**Assembly statistics**					
Total genome length (Mb)	915.8	841.2	696.5	923.3	922.8
Number of contigs	22,318	27,557	29,377	22,477	30,455
Mean contig size (kb)	41	30.5	23.7	41.1	30.3
Median contig size (kb)	15.1	9.3	10.2	17.2	4.8
Maximum contig size (kb)	919.8	715.6	826	829	809.4
N50 length (kb)	108.8	92.5	56.0	100.8	135.2
N50 No.	2320	2506	3316	2664	1943
**BUSCO completeness (303 genes, eukarota_odb9)**					
Complete, single copy (%)	84.5	82.5	70.3	88.78	76.90
Complete, duplicated (%)	1.3	0.0	1.3	1.32	2.31
Fragmented (%)	7.6	10.9	15.2	6.27	14.85
Missing (%)	6.6	6.6	13.2	3.63	5.94
**Gene statistics**					
No. of genes	12,652	12,490	12,853	12,072	12,771
Mean gene length (kb)	25.9	22.6	17.6	24.1	18.0
Mean CDS length (kb)	1.5	1.4	1.1	1.4	1.4
Mean intron length (kb)	4.2	4	3.6	4.2	4.0
Mean No. exons per gene	6.7	6.2	5.3	6.3	5.2
Annotated (%)					
InterPro	82	85	81	87	82
KEGG	40	41	34	43	43

CDS: coding sequence.

Genomic raw reads, genome assemblies, genome annotations, and raw transcriptomic (RNA-Seq) fastq files were uploaded and are available for download from the NCBI SRA [18], with all accession numbers and relevant metadata provided in Table 1. Supplementary Table S1 provides, for each of the species, complete gene lists and gene expression levels for each of the RNA-Seq samples. All results of the genome-wide selection scan are provided in Supplementary Table S2. For each orthologous group (OG) identified, Supplementary Table S3 provides complete gene lists, counts of genes per species, and mean gene expression levels from each the *Paragonimus* transcriptome datasets described above. All relevant software versions, as well as commands specifying the parameters used, are presented in Supplementary Text S1.

## Results and Discussion

### Genome features

**Figure 1:**
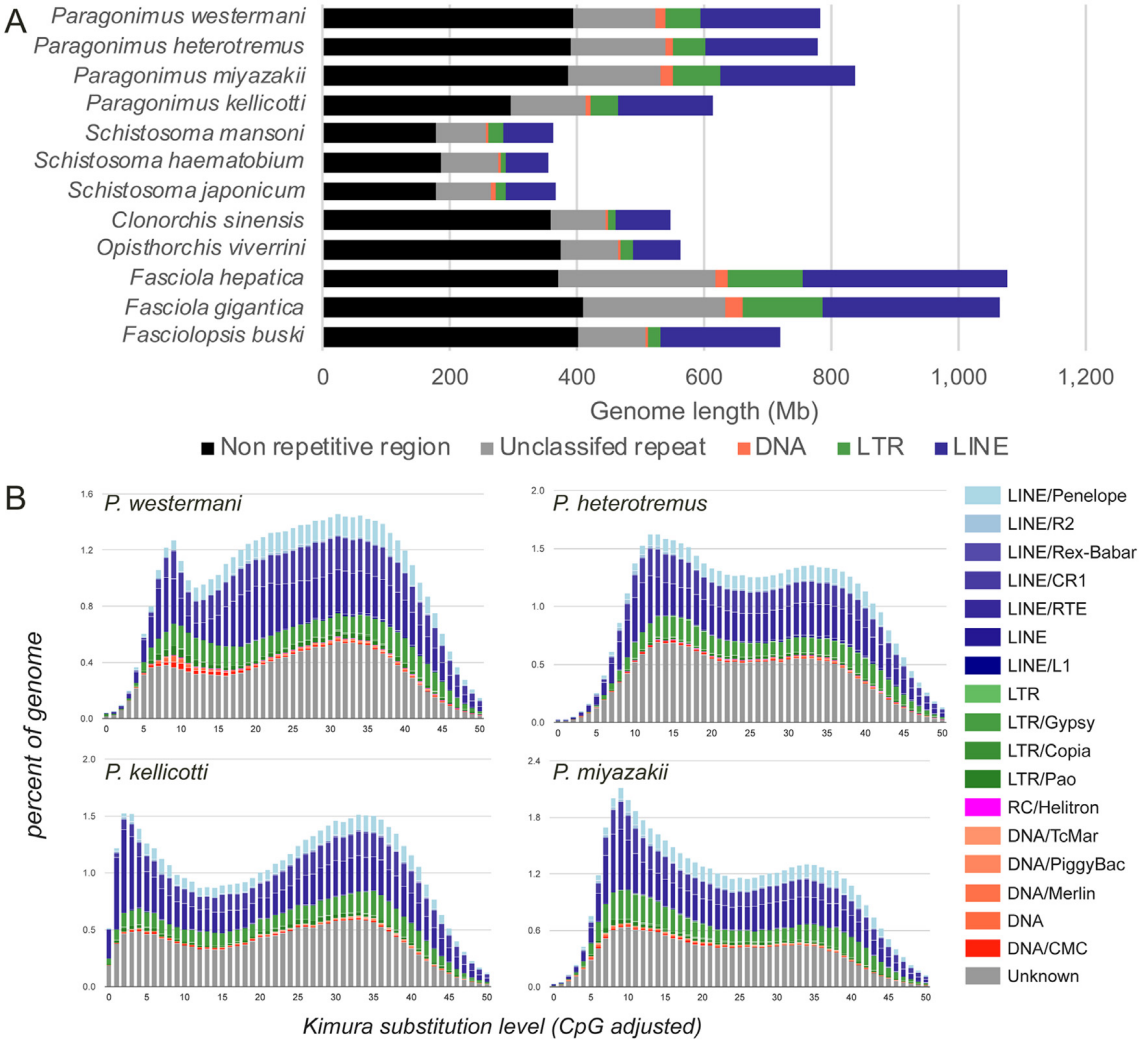
Comparisons of the overall content of the assembled *Paragonimus* genome assemblies. Comparisons are based on (**A**) length (including statistics for other sequenced trematode genomes) and (**B**) repeat landscapes, measured using the Kimura substitution level, which indicates how much a repeat sequence has degenerated since its incorporation into the genome (i.e., how recently the repeat sequence was added). The high peak at the far left of *P. kellicotti* indicates a recent incorporation or active transposable element activity. LINE: long interspersed nuclear element; LTR: long terminal repeat.

The sizes of the 4 newly generated *Paragonimus* genomes range from 697 to 923 Mb, containing between 12,072 and 12,853 genes. These draft genomes are estimated to be between 71.6% and 90.1% complete, according to the number of complete BUSCO eukaryote genes (single-copy or duplicate) [19], with the new *P. westermani* genome produced from a sample collected from Japan being more complete than the previously sequenced genome produced from a sample collected from India [11] (90.1% vs 70.2%, respectively; Table 2). Here, statements about *P. westermani* apply to the new Japanese genome unless otherwise stated. The total genome lengths of the *Paragonimus* spp. are larger than those of the Schistosomatidae and Opisthorchiidae but smaller than those of Fasciolidae. However, the total numbers of protein-coding genes are comparable (Table 2; complete gene lists for each species provided in Supplementary Table S1). Repetitive sequences occupy between 49% and 54% of the *Paragonimus* genomes (Fig. 1A). The repeat landscapes, depicting the relative abundance of repeat classes in the genome, vs the Kimura divergence from the consensus, revealed that *P. kellicotti* in particular has a significant number of copies of transposable elements (TEs) with high similarity to consensus (Kimura substitution level: 0–5), indicating recent and current TE activity (Fig. 1B). In a recent study [20], TE activity in the Fasciolidae was found to be low. TEs are potent sources of mutation that can rapidly create genetic variance, especially following genetic bottlenecks and environmental changes, providing bursts of allelic and phenotypic diversity upon which selection can act [21, 22]. Therefore, changes in TE activity, modulated by environmentally induced physiological or genomic stress, may have a major effect on adaptation of populations and species facing novel habitats and large environmental perturbations [23].

**Figure 2:**
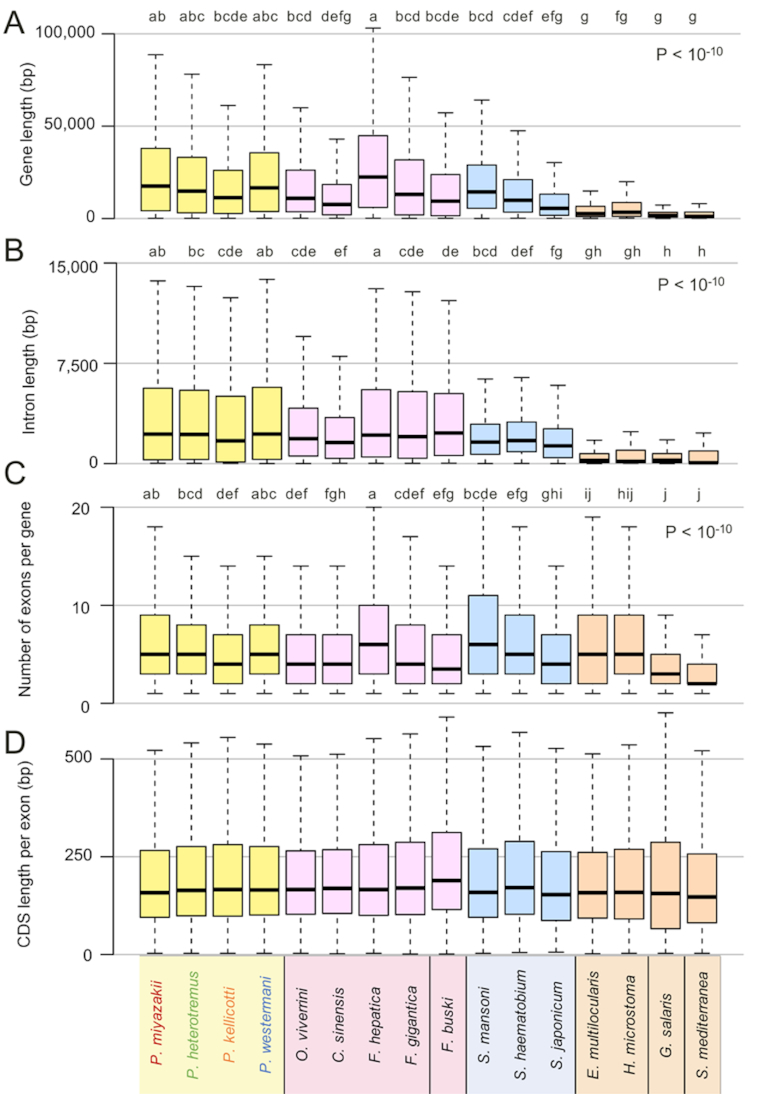
Comparison of genome annotation characteristics and attributes among several species of flatworms. Attributes characterized included (**A**) full gene lengths, including coding and noncoding sequences; (**B**) mean intron lengths per gene; (**C**) number of exons per gene; and (**D**) coding sequence (CDS) length per exon. *P*-values and letter groupings indicate significant differences among species, as calculated using ANOVA with Tukey HSD post hoc test (i.e., two species labeled the same letter in the group are not significantly different, P < 0.05). Boxes represent the interquartile ranges (IQRs) between the first and third quartiles, and the line inside the box represents the median value. Whiskers represent the lowest or highest values within values 1.5 times the IQR from the first or third quartiles.

Focusing on the gene content, *P. kellicotti* had the shortest mean total gene length among the species, and the lung flukes overall had similar gene lengths to other flukes, while platyhelminth species other than trematodes have shorter genes overall (Fig. 2A). The variability in gene lengths observed between species results from differences in both mean intron lengths (Fig. 2B) and the mean number of exons per gene (Fig. 2C), while the average coding sequence (CDS) lengths of the exons across all the platyhelminth species were similar to each other (Fig. 2D). Whereas there was species-to-species variability in gene lengths and exon counts, consistent patterns among the types of flukes were not apparent. Some of this variability may have arisen as a result of the variation in quality of the assemblies, but these differences were minimized by only using complete gene models with a start and stop codon identified in the same frame.

**Figure 3.**
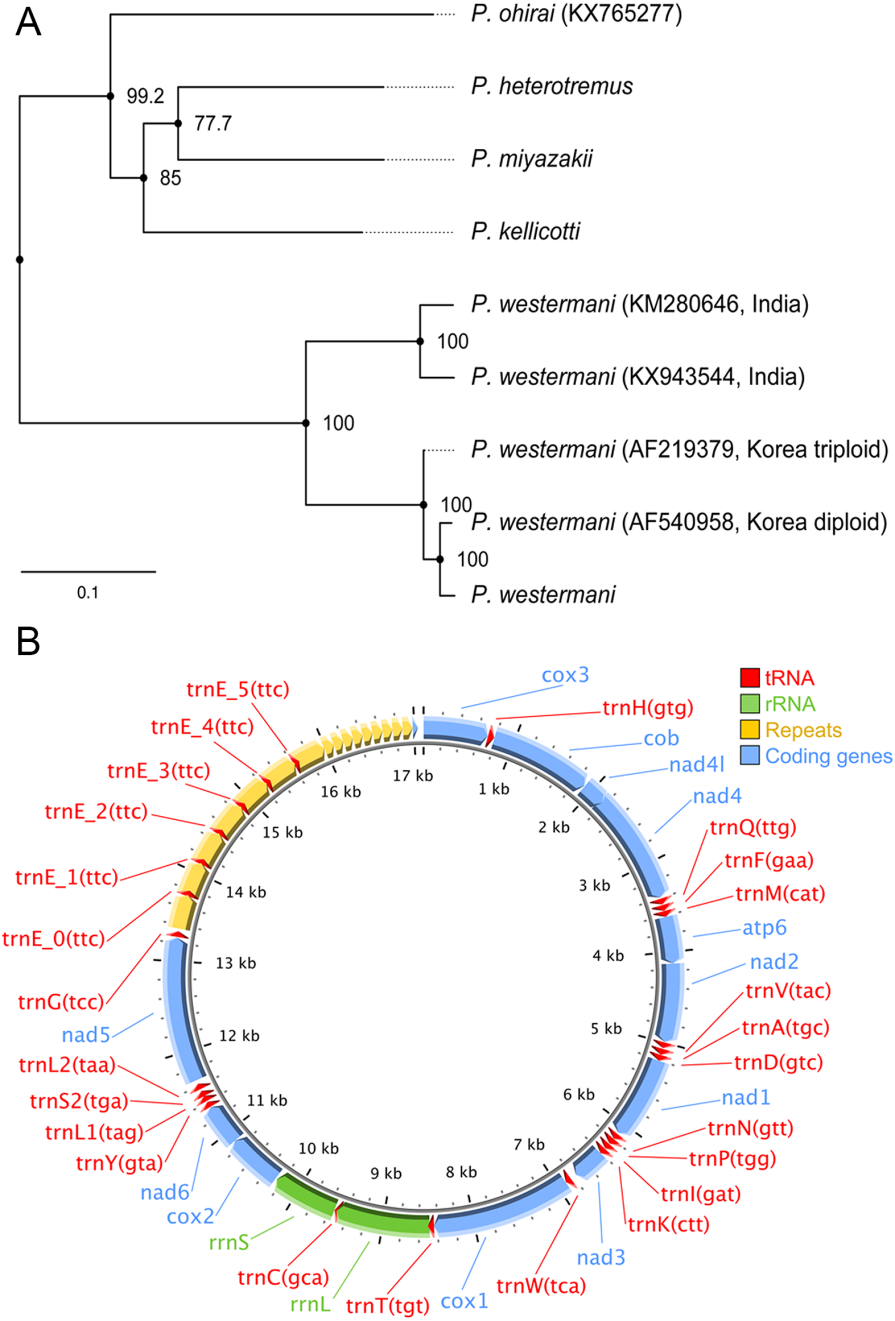
Clustering of *Paragonimus* species. (**A**) Mitochondrial whole-genome–based phylogeny, including previously sequenced *Paragonimus* mitochondrial genomes (with accessions indicated). The branch lengths represent nucleotide substitutions per site, and the numbers shown at nodes indicate SH-like approximate likelihood-ratio test (SH-aLRT) support values. (**B**) *Paragonimus kellicotti* mitogenome gene structure. rRNA: ribosomal RNA; tRNA: transfer RNA.

Mitochondrial whole-genome–based clustering was performed for the 4 *Paragonimus* species plus some additional existing previously sequenced mitochondrial genome assemblies for *P. ohirai* and 4 for *P. westermani* (Fig. 3A). This indicated that our Japanese *P. westermani* sample clustered with the existing known *P. westermani* samples from eastern Asia and that all the other 3 newly sequenced species were distinct from *P. ohirai*.

We generated a PacBio long-read–based mitochondrial assembly for *P. kellicotti*. The fully circularized complete genome was 17.3 kb in length, including a 3.7-kb non-coding repeat region between *tRNA^Gly^* and *cox3* (Fig. 3B). There are 7 copies of long repeats (378 bp) and 9.5 copies of short repeats (111 bp). The long repeats overlap with 6 copies of *tRNA^Glu^*. This structural organization of repeat sequences does not resemble those found in previous comparison of *Paragonimus ohirai* and *P. westermani* [11], where the non-coding region is partitioned by *tRNA^Glu^* into 2 parts.

**Figure 4:**
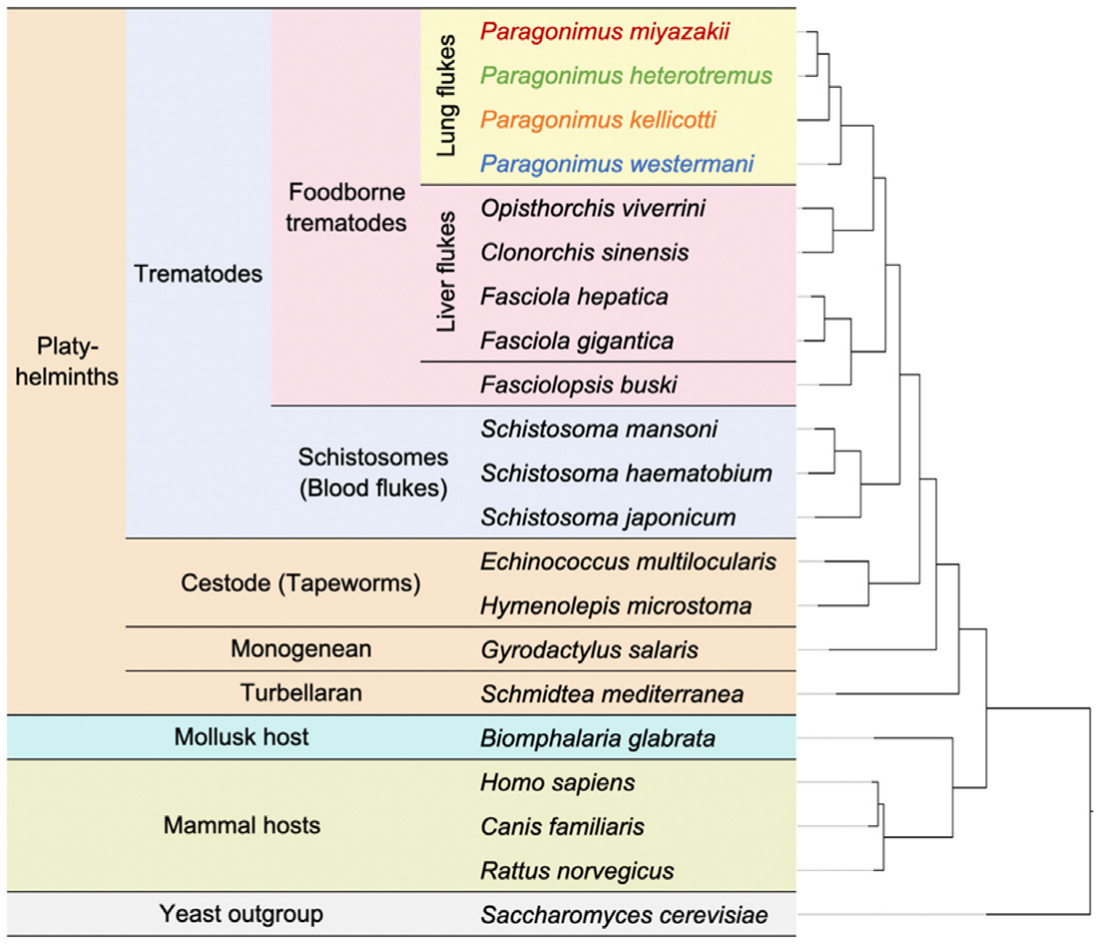
Species clustering based on single-member OPF sequences. A total of 262,720 genes (85% of all genes across the species) were assigned to 17,953 OPFs; 2,493 genes are in 326 species-specific OPFs.

Clustering based on nuclear genomes' single-member orthologous protein families (OPFs) of the 4 new lung flukes, 4 liver flukes, 3 blood flukes, 5 other platyhelminths, 4 host species, and a yeast outgroup was performed on the basis of the shared phylogeny among orthologous OPF groups. These findings mirrored the mitochondrial clustering results for the lung fluke species (Fig. 4), indicating that *P. westermani* is the earlier-diverging taxon, as previously suggested on the basis of ribosomal RNA [24].

Our *P. westermani* reference genome was assembled using samples collected from Japan (Amakusa, Kyushu). We compared the genomic sequences of our East Asian *P. westermani* to the recently published *P. westermani* genome from India (Changlang, Arunachal Pradesh) [11] to estimate the genetic divergence between geographically diverse samples. This analysis identified a mean nucleotide sequence identity of 87.6%.

### Gene-family dynamics identify expanded functions distinguishing lung fluke species

**Figure 5:**
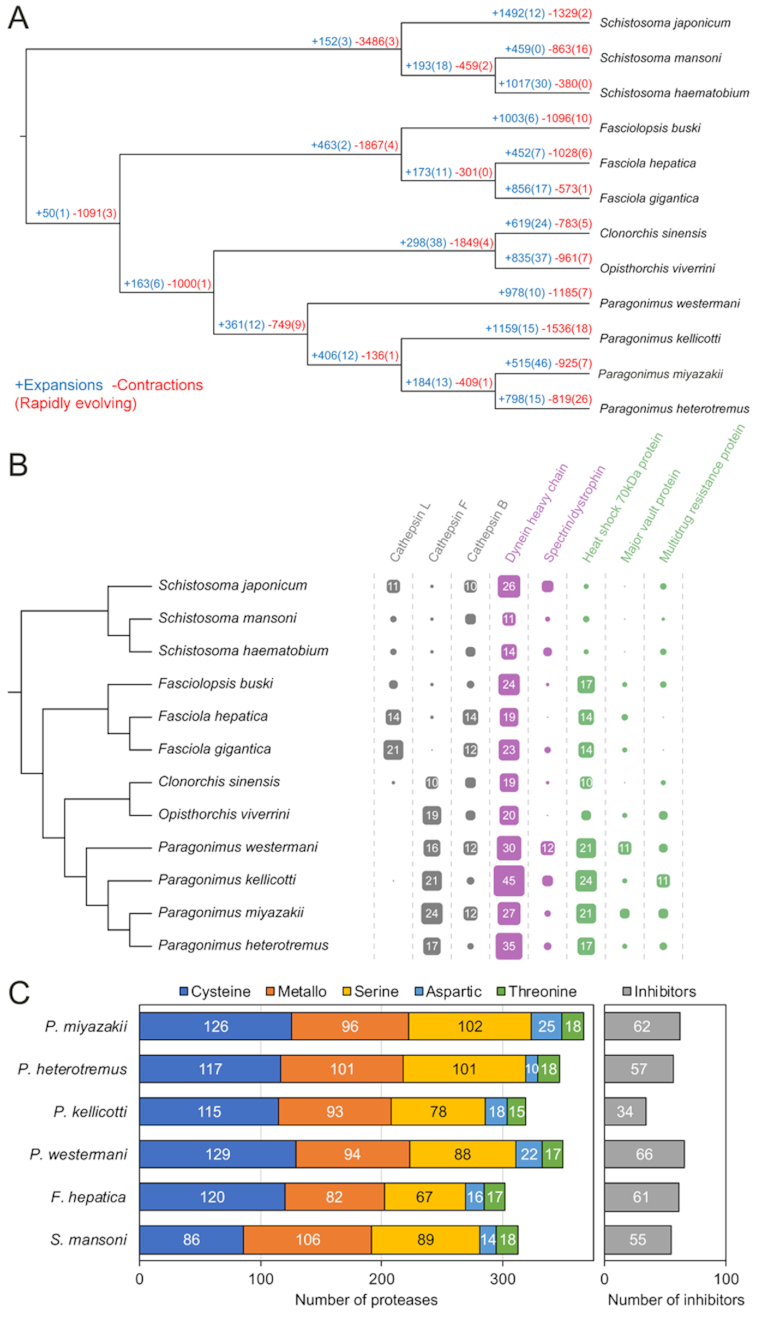
Gene-family dynamics among platyhelminth species. (**A**) Rapidly evolving families of interest are quantified at each stage of the phylogeny, including genes gained (blue) and lost (red) relative to other species. The numbers of rapidly evolving genes are indicated in parentheses. (**B**) Functionally annotated gene families of interest that displayed most pronounced differential expansions or contractions. (**C**) Overall protease and protease inhibitor abundance per species.

We investigated large-scale differences in gene complements among families of digenetic trematodes (Fig. 5A) and modeled gene gain and loss while accounting for the phylogenetic history of species [25]. Gene families of interest that displayed pronounced differential expansion or contraction (Fig. 5B) included the papain-family cysteine proteases, cathepsins L, B, and F, dynein heavy chain, spectrin/dystrophin, heat shock 70 kDa protein, major vault protein, and multidrug resistance protein. Total protease and protease inhibitor counts are shown in Fig. 5C. Cathepsin F genes may have roles in nutrient digestion and remodeling of other physiologically active molecules, and Ahn et al. [26] reported differential expression of cathepsin F genes during development of *P. westermani* and showed that most are highly immunogenic. This flagged them as prospective diagnostic targets. The importance of cathepsin F for *Paragonimus* contrasts with its function in the fasciolids, where cathepsin L genes are expanded and are thought to play a more critical role in host invasion [20, 27].

Differential expansion of cytoskeletal molecules is of interest in the context of tegument physiology [28]. Dynein is a microtubule motor protein, which transports intracellular cargo. Spectrin is an actin-binding protein, with a key role in maintenance of integrity of the plasma membrane. Dystrophin links microfilaments with extracellular matrix. The syncytial tegument of the surface of flatworms is a complex structure and a major adaptation to parasitism, and plays critical roles in nutrient uptake, immune response modulation and evasion, and other processes [28].

In *Paragonimus* spp., expanded gene families included heat shock proteins (HSPs), major vault proteins, and multidrug resistance proteins that play roles in maintaining cellular homeostasis under stress conditions. HSPs of flatworm parasites play a key role as molecular chaperones in the maintenance of protein homeostasis. They also are immunogenic and immunomodulatory. HSP is the most abundant family of proteins in the immature and mature egg of *Schistosoma mansoni*, and in the miracidium [29] and is highly abundant in the tegument of the adult schistosome [30]. In addition, HSP is abundant in the excretory/secretory products of the adult *Schistosoma japonicum* blood fluke [31]. HSP stimulates diverse immune cells, eliciting release of pro- and anti-inflammatory cytokines [32], and binds human low-density lipoprotein (the purpose of which is unknown but may be associated with transport of apoprotein B or in lipid trafficking [33]), and, given these properties, HSP represents a promising vaccine and diagnostic candidate [34]. Vaults, ribonucleoprotein complexes, are highly conserved in eukaryotes. Although their exact function remains unclear, it may be associated with multidrug resistance phenotypes and with signal transduction. In *S. mansoni*, up-regulation of major vault protein has been observed during the transition from cercaria to schistosomulum and in praziquantel-resistant adult worms [35]. ATP-binding cassette transporters (ABC transporters) are essential components of cellular physiological machinery, and some ABC transporters, including P-glycoproteins, pump toxins and xenobiotics out of the cell. Overexpression of P-glycoprotein has been reported in a praziquantel-resistant *S. mansoni* [36].

### Tetraspanin sequence evolution in *P. kellicotti*

**Figure 6:**
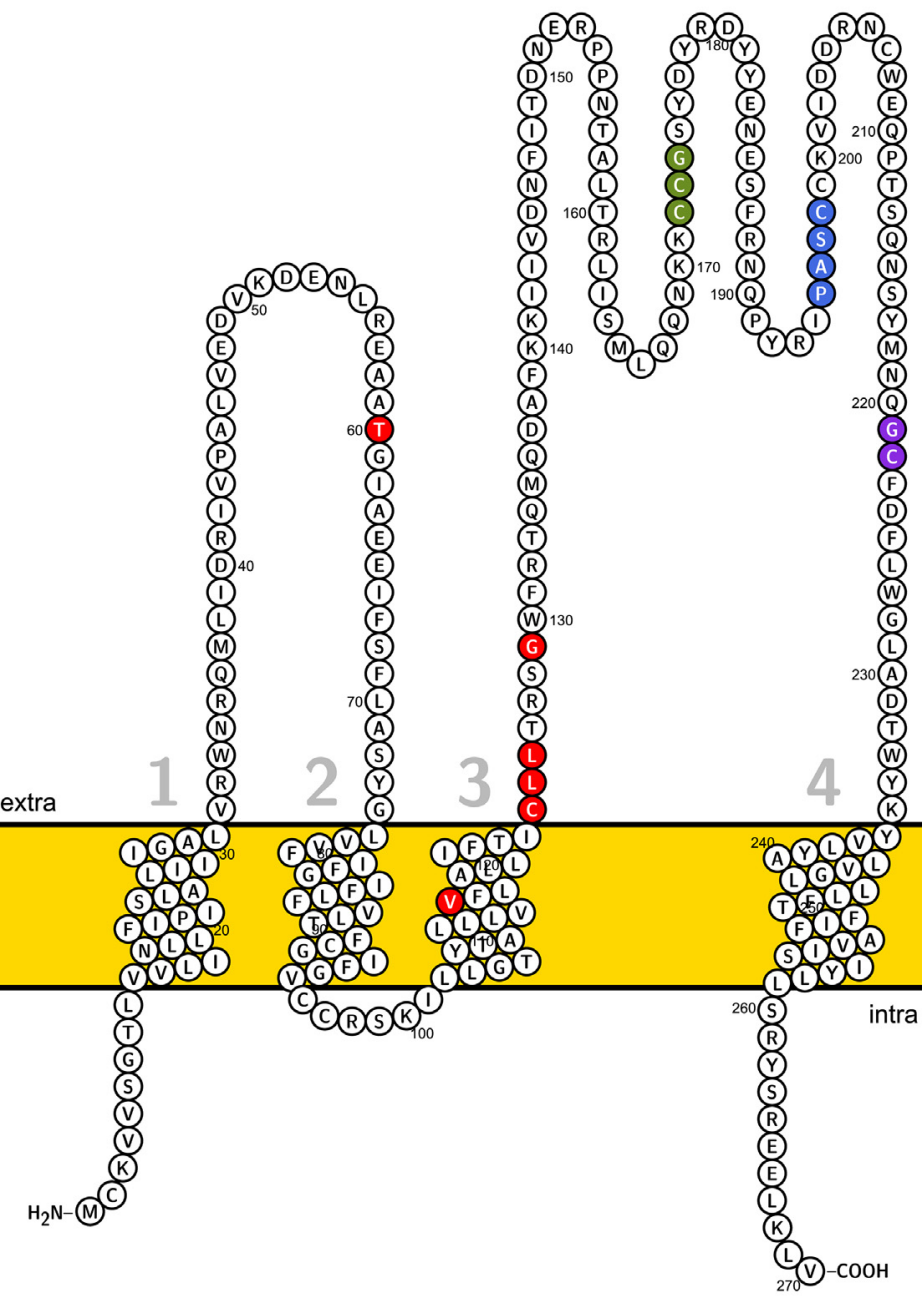
Predicted transmembrane helical topology of *Paragonimus kellicotti* tetraspanin (PKEL_00573). Amino acid sites under positive selection (red) and conserved motifs (CCG, PXSC, and GC motifs in green, blue and purple, respectively). The “PXSC” motif here is represented by the “PASC” sequence.

We searched for genes that evolved under positive selection in the 4 *Paragonimus* spp. based on the non-synonymous to synonymous substitution rate ratio (d_N_/d_S_). We conducted the branch-site test of positive selection to identify adaptive gene variants that became fixed in each species [37] (Supplementary Table S2). A tetraspanin from *P. kellicotti* (PKEL_00573) reached statistical significance after correction for multiple testing (d_N_/d_S_ = 9.9, false discovery rate = 0.018). Tetraspanins are small integral proteins bearing 4 transmembrane domains, which form 2 extracellular loops [38]. In trematodes, they are major components of the tegument at the host-parasite interface [39], are highly immunogenic vaccine antigens [40, 41], and may play a role in immune evasion [42]. In the tetraspanin sequence of *P. kellicotti*, we detected 6 amino acid sites under positive selection (Fig. 6). Five of the 6 sites were predicted to be located within the extracellular loops believed to interact with the immune system of the host. A similar pattern of positive selection within regions that code for extracellular loops has been reported in tetraspanin-23 from African *Schistosoma* species [43].

### Gene phylogeny analysis identifies functions conserved and specific to fluke groups

**Figure 7:**
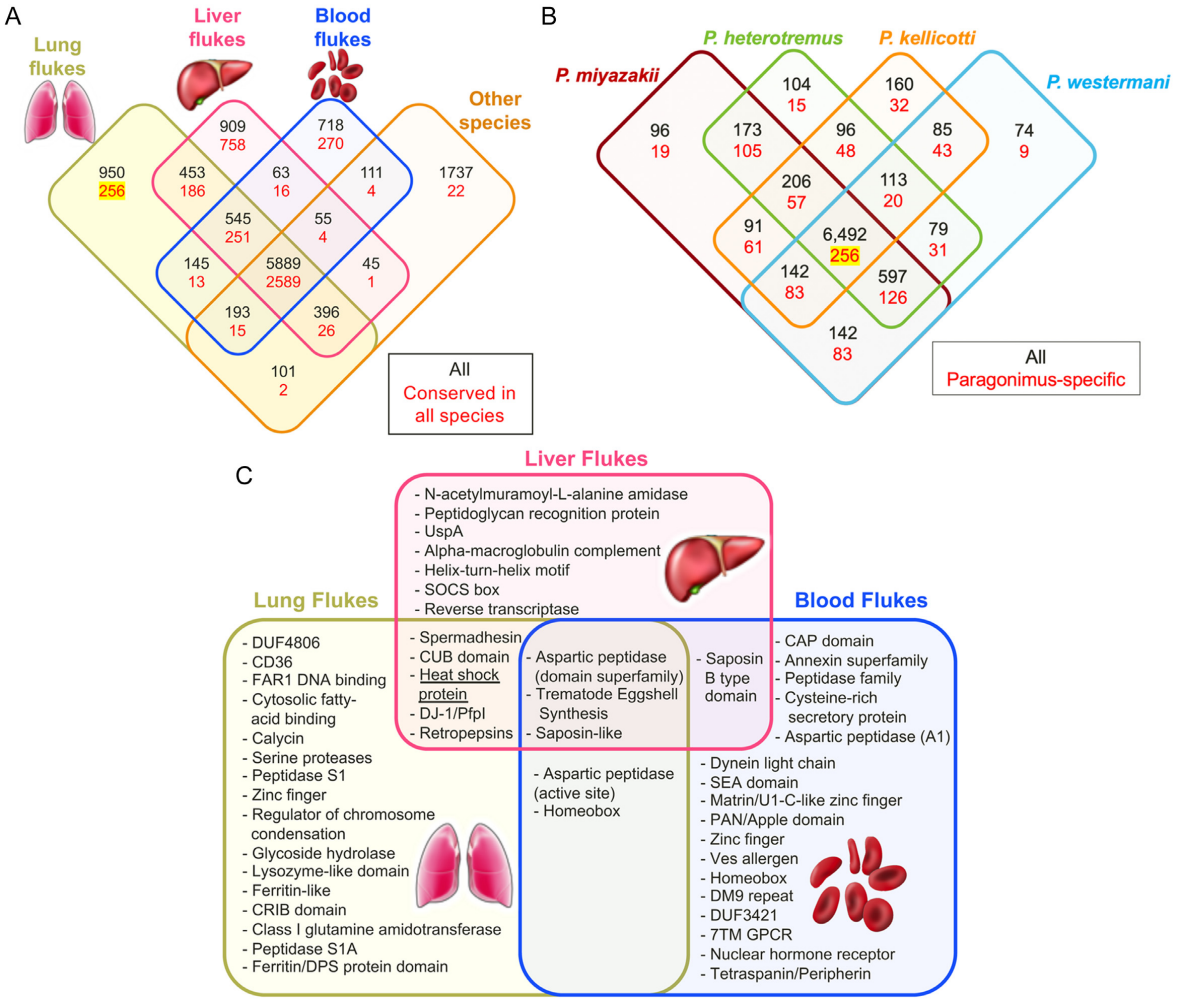
Orthologous Group (OG) distribution analysis. (**A**) OGs identified among groups of flukes. The OGs conserved in ≥1 of the species from each group are indicated in black, and the OGs conserved among all the species in the overlapping groups are indicated in red. (**B**) Counts of OGs among the 4 *Paragonimus* species, with *Paragonimus*-specific gene sets indicated in red. The 256 *Paragonimus* conserved-and-specific genes are highlighted in yellow. (**C**) Significant functional enrichment (Interpro domains) among the gene sets conserved among, and specific to, each major group of flukes (256, 758, and 270 OPFs in lung, liver, and blood flukes, respectively), relative to the functions in the complete gene sets.

**Figure 8:**
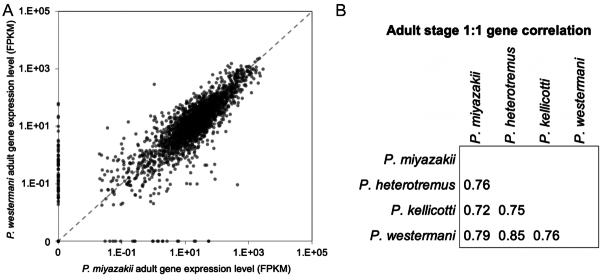
Analysis of gene expression data for species of lung flukes of the genus *Paragonimus*. (**A**) Comparison of adult-stage gene expression levels among 1:1 orthologs shared by *P. westermani* and *P. miyazakii*. Pearson correlation = 0.79. (**B**) Pearson correlation values between all lung fluke species for the adult-stage expression levels of all 1:1 orthologous genes.

We classified OGs on the basis of phylogenetic distribution of proteins from each of the 21 species (Fig. 4). Complete gene counts and lists per species and per OG are provided in Supplementary Table S3. These results were parsed to identify the OGs containing members among the platyhelminth species, and those that were conserved across all members of each group (lung, liver, and blood flukes and other platyhelminth species; Fig. 7A). This analysis identified 256 OGs that were conserved among, and exclusive to, the lung flukes (Fig. 7A and B). The lung fluke–conserved and –specific genes were significantly enriched for several gene ontology (GO) terms (Table 3; using *P. miyazakii* genes to test significance), most of which were related to peptidase activity (including serine proteases, which are involved in host tissue invasion, anticoagulation, and immune evasion [44]), as well as “iron binding” (which may be related to novel iron acquisition mechanisms from host tissue, which is not well understood in most metazoan parasites but has been described in schistosomes [45]). Lung (adult) stage RNA-Seq datasets were collected for each of the 4 lung fluke species (accessions in Table 1), and reads were mapped to each of their respective genomes. Based on the 1:1 gene orthologs (as defined by the previously described OG dataset), the orthologous genes across the lung flukes had consistent adult-stage gene expression levels, with Pearson correlations ranging from 0.72 to 0.85 (Fig. 8A and B).

**Table 3:** “Molecular Function” Gene Ontology terms enriched among *P. miyazakii* genes that are conserved among and exclusive to lung flukes

GO ID	GO term name	P value	No. conserved and specific	Total No. in genome
GO:0004175	Endopeptidase activity	5.2E−05	8	132
GO:0008236	Serine-type peptidase activity	5.6E−05	6	67
GO:0017171	Serine hydrolase activity	5.6E−05	6	67
GO:0 004252	Serine-type endopeptidase activity	1.6E−04	5	51
GO:00 70011	Peptidase activity, acting on L-amino acid peptides	6.1E−04	9	237
GO:0 008233	Peptidase activity	8.7E−04	9	249
GO:0 004568	Chitinase activity	2.1E−03	2	7
GO:0 004190	Aspartic-type endopeptidase activity	1.1E−02	2	16
GO:00 70001	Aspartic-type peptidase activity	1.1E−02	2	16
GO:0 008199	Ferric iron binding	1.1E−02	2	16

Expansion of unique aspartic proteases (including those predicted to be retropepsins) and other peptidases in the lung flukes may be associated with digestion of ingested blood, given the key role of this category of hydrolases and their inhibitors in nutrition and digestion of hemoglobin by schistosomes, and indeed other blood-feeding worms including hookworms [46, 47]. Given that pulmonary hemorrhage and hemoptysis are cardinal signs of lung fluke infection, it can be anticipated that the lung flukes ingest host blood when localized at the ulcerous lesion induced in the pulmonary parenchyma by infection. Overall, protease counts across species were similar (Fig. 5C) although *P. kellicotti* had substantially fewer protease inhibitors compared to the other *Paragonimus* species (34 vs 57, 62, and 66), *Fasciola hepatica* (61), and *S. mansoni* (55). Protease inhibitors in flukes are thought to be important for creating a safe environment for the parasite inside the host by inhibiting and regulating protease activity and immunomodulation [91], so this may suggest a novel host interaction strategy by *P. kellicotti*.

**Figure 9:**
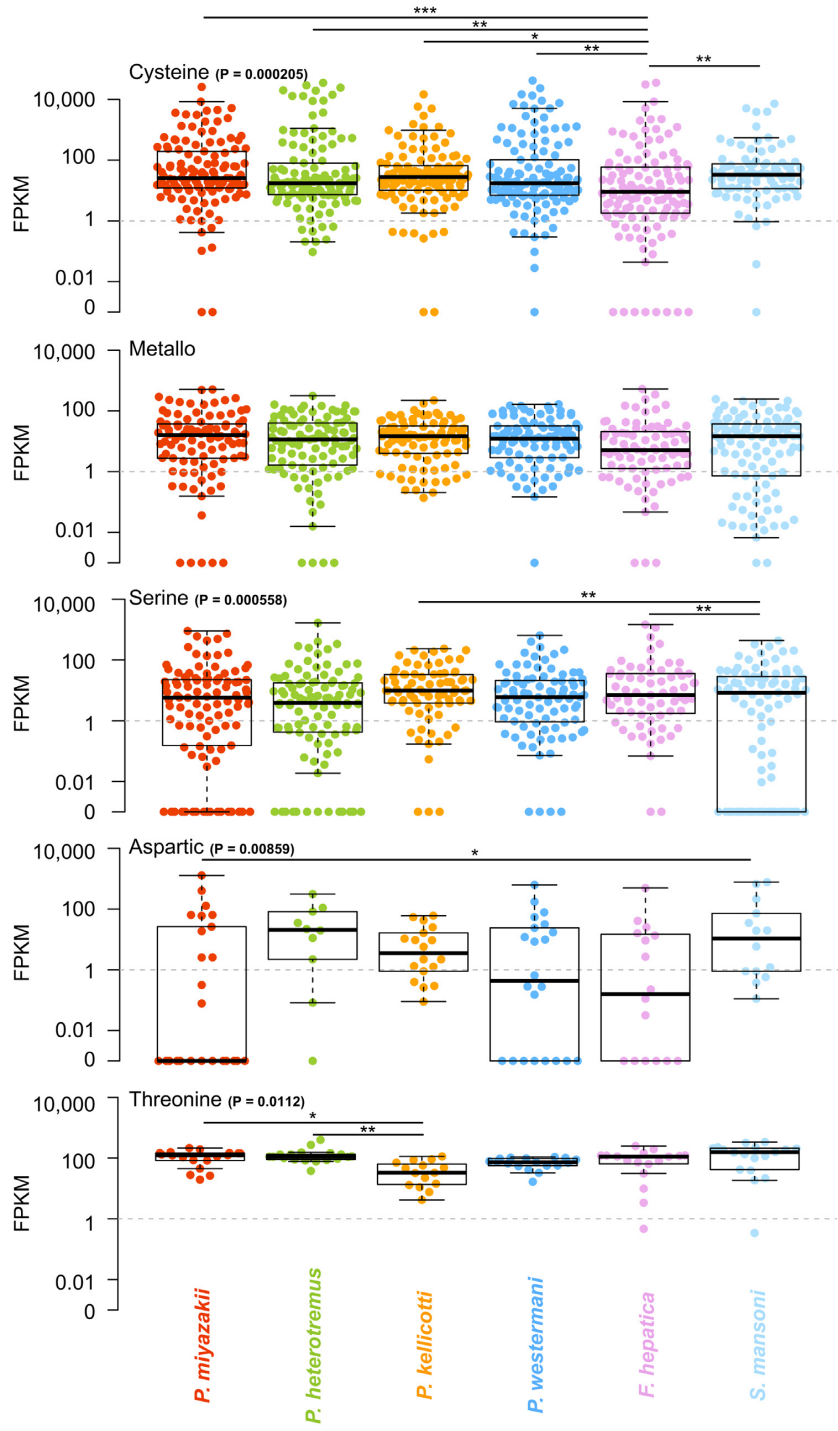
A comparison of adult-stage protease gene expression levels in the 4 *Paragonimus* species, *F. hepatica*, and *S. mansoni*. Boxes represent the interquartile ranges (IQRs) between the first and third quartiles, and the line inside the box represents the median value. Whiskers represent the lowest or highest values within values 1.5 times the IQR from the first or third quartiles. ANOVA P values are shown, and significant pairwise T-test comparisons are indicated with lines (* P ≤ 0.05, ** P ≤ 0.01, *** P ≤ 0.001).

Analysis of the adult-stage gene expression levels of the discrete protease classes (Fig. 9) did not identify substantial differences among the *Paragonimus* species, except for a lower expression of threonine proteases in *P. kellicotti*. During the adult stage, cysteine proteases in all *Paragonimus* species exhibited significantly higher expression overall compared to *F. hepatica*, but expression levels similar to those of *S. mansoni*. A previous study identified immunodominant excretory-secretory cysteine proteases of adult *P. westermani* involved in immune evasion [48], and another study identified critical roles for excretory-secretory cysteine proteases during tissue invasion by newly excysted metacercariae of *P. westermani* [49]. The rapid diversification and critical host-interaction functions of the proteases highlights their importance, both in terms of understanding *Paragonimus* biology and in terms of identifying targets for control.

Functional enrichment analysis among the lung, liver, and blood fluke conserved-and-exclusive OGs (Fig. 7C) indicated that each family of fluke has evolved a distinct set of aspartic peptidases, trematode eggshell synthesis genes, and saposin-like genes (which interact with lipids and are strongly immunogenic during fascioliasis [50]). The lung flukes, meanwhile, have uniquely expanded sets of serine proteases, as well as other gene families with functions including FAR1 DNA binding (a class of proteins that are important secreted host-interacting proteins in some parasitic nematodes [51]), fatty acid binding, and ferritin-like functions (intracellular proteins involved in iron metabolism, localized in vitelline follicles and eggs [52]).

### Treatments, vaccine targets, and diagnostics

The World Health Organization currently recommends the use of praziquantel or, as a backup, triclabendazole for the treatment of paragonimiasis; both are highly effective for curing infections [53]. However, there are concerns about the development of resistance to these drugs; triclabendazole resistance of *P. westermani* was reported in a human case from Korea [54]. Furthermore, there is widespread resistance to triclabendazole in liver flukes in cattle in Australia and South America [55], and praziquantel resistance is anticipated in the future owing to its widespread use as a single treatment for schistosomiasis, a worrisome situation that has encouraged the search for novel drugs [56]. The comparative analysis presented here identifies valuable putative protein targets for drug development, including *Paragonimus*-specific proteins and trematode-conserved proteins that do not share orthology to human proteins. The protein annotation data available in Supplementary Table S1 also will enable prioritization including biological functional annotations [57, 58], protein weight and pi predictions [59], predictions of signal peptides and transmembrane domains [60] and cellular compartment localization [57], and sequence similarity matches to targets in the ChEMBL database [61]. This information can provide a starting point for future bioinformatic prioritization and drug testing.

Vaccination to prevent future infections would offer an attractive alternative to treatment, but development of vaccine protection against trematode infection has so far been unsuccessful and is unlikely to be practical for paragonimiasis in the near future [62]. However, the complete genome sequences and comparative analysis of the gene sets presented here provide valuable resources for future vaccine target development.

**Figure 10:**
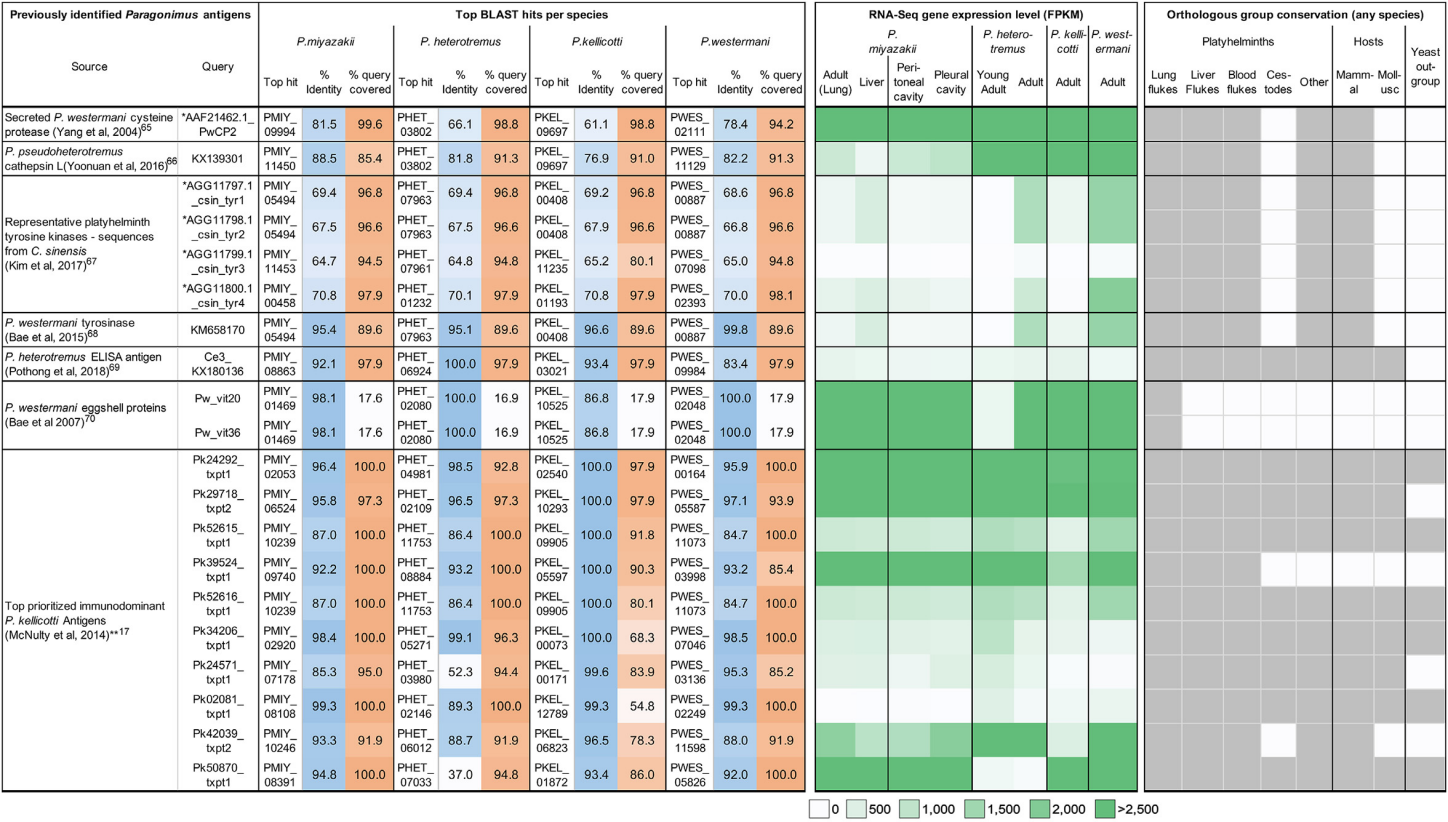
Gene matches, expression level, and orthology for previously identified *Paragonimus* antigens. Top gene matches in each species (Diamond blastp) are shown, and the percent identity and percentage of the query sequence covered with the match are shown. Gene expression data correspond to the matched gene for each species, and orthology data indicate the conservation of the matched proteins according to the Orthologous Group analysis (dark grey = ortholog present in ≥1 species in group). *Query sequence was an amino acid sequence instead of a nucleotide sequence. **Of the top 25 *P. kellicotti* immunodominant antigen transcripts identified by McNulty and coworkers [17], the 10 best matches are presented (in terms of percent identity between the assembled transcript and the annotated gene). For the other 3 species, the BLAST searches were performed against the orthologous gene in *P. kellicotti*, not the original transcript sequence.

Pulmonary paragonimiasis is frequently mistaken for tuberculosis or pneumonia, and often patients do not shed eggs, which leads to false-positive diagnoses of other conditions such as malaria or pneumonia [4, 63, 64]. This highlights a pressing need for accurate, rapid, and affordable diagnostic approaches for paragonimiasis, a topic that has been the focus of numerous reports. We performed BLAST sequence similarity searches of previously identified *Paragonimus* diagnostic antigen targets among the 4 species (Fig. 10). These included (i) *P. westermani* and *Paragonimus pseudoheterotremus* cysteine proteases identified in 2 previous studies [65, 66] (matching to the same protein targets from both studies in *P. heterotremus* and *P. kellicotti*), 1 of which had high adult-stage expression levels in all 4 species [65]; (ii) 3 different tyrosine kinases (1 of which was identified in 2 different studies, in *Clonorchis sinensis* and in *P. westermani* [67, 68]), all of which had relatively low gene expression levels in adult stages; (iii) a previously unannotated *P. heterotremus* ELISA antigen [69] with low expression across life cycle stages, which we now annotate as a saposin protein (which we found to rapidly evolve among flukes [Fig. 7C] and which is strongly immunogenic in fascioliasis [50]); and (iv) eggshell proteins of *P. westermani* [70], for which we now provide full-length sequences. We observed that this gene was conserved across and specific to the lung flukes, with lower gene expression in the young adult stage (*P. heterotremus*) but higher expression in the adult stages of all species; (v) among serodiagnostic *P. kellicotti* antigens based on a transcriptome assembly and proteomic evidence [16], we identified the top 10 of the 25 prioritized transcripts that best matched between the transcript sequence and the newly annotated draft genome of *P. kellicotti*. Thereafter, the full-length gene sequence in *P. kellicotti* was used to query the other species. Several of these were highly expressed in the adult stage of all 4 species, including 1 that is fluke specific (PKEL_0 5597). However, not all of these had high sequence conservation across all species, with 2 only having weak hits in *P. heterotremus* (PKEL_00171 and PKEL_0 1872).

As a result of this newly developed genomic resource for the lung flukes, previously identified diagnostic targets were identified with full gene sequences across all 4 species. The complete gene sequences, conservation information, and transcriptomic gene expression data for these target proteins can allow for optimization of the targets for diagnostic testing that is effective on species spanning the genus (Fig. 10). This is noteworthy given the absence of a standardized, commercially available test for serodiagnosis for human paragonimiasis.

## Conclusion

To substantially improve our understanding of the lung flukes at the molecular level, we sequenced, assembled, annotated, and compared draft genomes of 4 species of *Paragonimus*, 3 from Asia (*P. miyazakii, P. westermani* from Japan, *P. heterotremus*) and 1 from North America (*P. kellicotti*), thereby providing novel and valuable genomic resources across these important parasites for the first time. We have used these new resources to compare and analyze phylogenies, to identify gene sets and biological functions associated with parasitism in lung flukes, and to contribute a key resource for future investigation into host-parasite interactions for these poorly understood agents of neglected tropical disease. Our identification of previously prioritized *Paragonimus* diagnostic markers in each of the 4 lung fluke species revealed that the same protein targets were identified in multiple studies, and hence the availability of full gene sequences now should facilitate diagnostic assays aiming for reactivity across all species of lung fluke. Overall, the novel genomic and transcriptomic resources developed here will be invaluable for research on paragonimiasis, guiding experimental design and generation of novel hypotheses.

## Methods

### Parasite specimens

Samples of DNA and RNA of *Paragonimus westermani* were sourced in Japan. *Paragonimus heterotremus* (LC strain, Vietnam) were recovered from a cat experimentally infected with metacercariae from Lai Chau province, northern Vietnam (70% ethanol preserved; whole worm). *Paragonimus miyazakii* metacercariae were recovered from freshwater crabs (*Geothelphusa dehaani*), collected in Shizuoka Prefecture, central Japan [15], and were raised to adulthood in rats. DNA and RNA samples were prepared for each of the (pre-)adult flukes recovered from the lungs and from the pleural and peritoneal cavities of experimentally infected rats. *Paragonimus kellicotti* adult worms for genome sequencing were recovered from the lungs of Mongolian gerbils infected in the laboratory with metacercariae recovered from Missouri crayfish [71].

### Genome sequencing, assembly, and annotation

DNA and RNA samples were collected from parasites of 4 distinct *Paragonimus* species: *P. miyazakii* (Japan), *P. heterotremus* (LC strain, Vietnam), *P. kellicotti* (Missouri, USA), and *P. westermani* (Japan). Illumina DNA sequencing produced fragments, 3- and 8-kb insert whole-genome shotgun libraries, and PacBio reads were generated for *P. kellicotti*. The sequences were generated on the Illumina platform and assembled using Allpaths_LG [72]. Scaffolding was improved using an in-house tool called Pygap (gap closure tool), the Pyramid assembler with Illumina paired reads to close gaps and extend contigs, and L_RNA_scaffolder [73], which uses transcript alignments to improve contiguity. For *P. kellicotti*, Nanocorr [74] was used to perform error correction on the PacBios data and PBJelly was used to fill gaps and improve the Illumina allpaths assembly using the PacBio reads [75]. The nuclear genomes were annotated using the MAKER pipeline v2.31.8 [76]. Repetitive elements were softmasked with RepeatMasker v4.0.6 using a species-specific repeat library created by RepeatModeler v1.0.8, RepBase repeat libraries [77], and a list of known transposable elements provided by MAKER [76]. RNA-seq reads were aligned to their respective genome assemblies and assembled using StringTie v1.2.4 [78] (*P. miyazakii* samples collected from stages in the liver, peritoneal cavity [2 replicates], lung [adult], and pleural cavity; *P. heterotremus* samples from adults and young adults [2 replicates]; *P. westermani* [16] and *P. kellicotti* [17] adult-stage transcriptomic reads were retrieved from published reports). The resulting alignments and transcript assemblies were used by BRAKER [79] and MAKER pipelines, respectively, as extrinsic evidence. In addition, messenger RNA (mRNA) and EST sequences for each species were retrieved from NCBI and were provided to MAKER as protein homology evidence along with protein sequences from UniRef100 [80] (Trematoda-specific, n = 205,161) and WormBase ParaSite WBPS7 [81]. *Ab initio* gene predictions from BRAKER v2 [79] and AUGUSTUS v3.2.2 (trained by BRAKER and run within MAKER) were refined using the transcript and protein evidence. Previously unpredicted exons and untranslated regions were added, and split models were merged. The best-supported gene models were chosen on the basis of annotation edit distance (AED) [82]. To reduce false-positive results, gene predictions without supporting evidence were excluded in the final annotation build, with the exception of those encoding Pfam domains, as detected by InterProScan v5.19 [57]. These Pfam encoding domains were rescued in order to improve the annotation accuracy overall by balancing sensitivity and specificity [76, 83]. Gene products were named using PANNZER2 [84] and sma3s v2 [85]. Table 1 provides details of database accessions for the genomes. The completeness of annotated gene sets was assessed using BUSCO v3.0, eukaryota_odb9 [19]. GO, KEGG, and protease annotations were performed using InterProScan v5.19 [57], GhostKOALA [58], and MEROPS [86], respectively. ExPASy was used to perform protein weight and pi predictions [59], SignalP was used to predict signal peptides and transmembrane domains [60], and gene product localization was predicted using the “cellular component” GO annotations provided by InterProScan [57].

Functional enrichment testing was performed using GOSTATS [87] for GO enrichment and negative binomial distribution tests for InterPro domain enrichment (minimum 3 annotated genes required for significant enrichment). Ribosomal RNAs and tRNAs were annotated using RNAmmer v1.2.1 [88] and tRNAscan-SE v1.23 [89], respectively. Genome characteristics and statistics including CDS, numbers and lengths of genes, exons and introns were defined using the longest complete mRNA (with start and stop codon) for each gene. Across the 4 species of *Paragonimus*, complete mRNAs were found for an average of 86.2% of all annotated genes.

Assembly of the mitochondrial genome of *P. kellicotti* was achieved using CANU [90] to align PacBio long reads, followed by error correction using Pilon [91].

MUMmer v4.0 [92] was used to estimate the level of genetic divergence between *P. westermani* samples from Japan and India. Nucmer was run first to generate genome alignments using draft assembly sequences. Dnadiff was then used to calculate the average sequence identity between the genomes considering only 1-to-1 alignments.

### Transcriptome datasets and gene functional annotations

RNA-seq datasets were trimmed for adapters [93] and aligned [94] to their respective genome assemblies, and gene expression levels (FPKM) were quantified per gene per sample in each of the 4 species [95]. Interpro domains and GO terms [57], KEGG enzymes [58], and protease [86] annotations of the genes were used to identify putative functions of genes of interest and perform pathway enrichment [87]. All raw RNA-seq fastq files were uploaded to the NCBI SRA [18], and complete sample metadata and accession information are provided in Table 1. Supplementary Table S1 provides, for each of the species, complete gene lists and gene expression levels for each of the RNA-seq samples. Complete functional annotations for every gene are also provided for *P. miyazakii* in this table.

### Repeat analysis

RepeatModeler v1.0.8 (with WU-BLAST as its search engine) was used to build, refine, and classify consensus models of putative interspersed repeats for each species. With the resulting repeat libraries, genomic sequences were screened using RepeatMasker v4.0.6 in “slow search” mode to generate a detailed annotation of the interspersed and simple repeats. Per-copy distances to consensus were calculated (Kimura 2-parameter model, excluding CpG sites) and were plotted as repeat landscapes where divergence distribution reflected the activity of TEs on a relative time scale per genome using the calcDivergenceFromAlign.pl and createRepeatLandscape.pl scripts included in the RepeatMasker package.

### Gene family evolution

OGs of genes of 21 species were inferred with OrthoFinder v1.1.4 [96] using the longest isoform for each gene (*Paragonimus* genome source information in Table 1; worm gene sets were retrieved from WormBase ParaSite in June 2017 [81]; outgroup species gene sets were retrieved from Ensembl in June 2017 [97]). The CAFE method [25] was used to model gene gain and loss while accounting for the species' phylogenetic history based on an ultrametric species tree and the number of gene copies found in each species for each gene family. Birth-death (λ) parameters were estimated and the statistical significance of the observed family size differences among taxa were assessed. Results from OrthoFinder [96] were parsed to identify the OGs of interest based on conservation, including the lung fluke–conserved, liver fluke–conserved, and blood fluke–conserved OGs and gene sets per species. Supplementary Table S3 provides details of full OG counts per species and gene membership.

We used PosiGene [98] to search genome-wide for genes that evolved under positive selection based on the non-synonymous to synonymous substitution ratio. TMMOD [99] and Protter [100] were used for transmembrane helical topology prediction and visualization, respectively. We searched for genes that evolved under positive selection in the 4 *Paragonimus* spp. based on the non-synonymous to synonymous substitution rate ratio (d_N_/d_S_). We conducted the branch-site test of positive selection to identify adaptive gene variants that became fixed in each species [37].

### Previously identified *Paragonimus* diagnostic antigen search

Nucleotide sequences (or, if unavailable, amino acid sequences) were retrieved from each of the cited publications (Fig. 10). Diamond blastx (nucleotides; v0.9.9.110) or Diamond blastp (amino acids; v0.9.9.110) were used to identify the top hit gene in each *Paragonimus* genome annotation (default settings). The best BLAST E-value was used to identify the top match, followed by top bit score, length, and percent ID in the case of ties. For the top 25 *P. kellicotti* immunodominant antigen transcripts identified in McNulty et al. 2014 [17], matches were identified between the assembled transcript and the annotated gene. For the other 3 species, the BLAST searches are performed against the identified *P. kellicotti* gene and not the original transcript sequence.

### RNA-seq–based gene expression profiling

After adapter trimming using Trimmomatic v0.36 [93], RNA-seq reads were aligned to their respective genome assemblies using the STAR aligner [94] (2-pass mode, basic). All raw RNA-seq fastq files were uploaded to the NCBI SRA [18], and complete sample metadata and accession information are provided in Table 1. Read fragments (read pairs or single reads) were quantified per gene per sample using featureCounts (version 1.5.1) [95]. FPKM (fragments per kilobase of gene length per million reads mapped) normalization was also performed. Pearson correlation–based RNA-seq sample clustering was performed in R (using the hclust package, complete linkage).

### Statistics

ANOVA analysis followed by Tukey HSD post hoc testing was performed to compare genome statistics and protease expression between species (Figs 2 and 9). Because comparisons for the genome statistics by *t* tests involved large numbers of values, which can falsely indicate positive statistical significance, a random selection of 100 values from each species was used (excluding the top and bottom 1% of data to avoid outliers). For Figure 2, Letter labels above the species indicate statistical groups; i.e., if 2 species share the same letter then they were not statistically significantly different. For Figure 3, individual pairwise significance values are indiciated since there were fewer differences between species. from each other.

## Availability of Supporting Data and Materials

Genomic raw reads, genome assemblies, genome annotations, and raw transcriptomic (RNA-seq) fastq files were uploaded and are available for download from the NCBI SRA [18], with all accession numbers and relevant metadata provided in Table 1. Supplementary Table S1 provides, for each of the species, complete gene lists and gene expression levels for each of the RNA-seq samples. Other data further supporting this work are openly available in the *GigaScience* repository, GigaDB [101].

## Additional Files

**Supplementary Table S1**: Gene expression and orthologous group data for each gene, for the 4 *Paragonimus* species: (A) *P. miyazakii*, (B) *P. heterotremus*, (C) *P. kellicotti*, (D) *P. westermani* (provided as a separate MS Excel database).

**Supplementary Table S2:** Genome-wide selection scan results for all *Paragonimus* species (provided as a separate MS Excel database).

**Supplementary Table S3:** Complete Orthologous Group (OG) counts per species, gene membership, and average *Paragonimus* gene expression levels per RNA-seq sample (provided as a separate MS Excel database).

**Supplementary Text S1**. Commands and parameters for analyses (provided as a separate MS Word file).

giaa073_GIGA-D-19-00411_Original_Submission

giaa073_GIGA-D-19-00411_Revision_1

giaa073_GIGA-D-19-00411_Revision_2

giaa073_GIGA-D-19-00411_Revision_3

giaa073_Response_to_Reviewer_Comments_Original_Submission

giaa073_Response_to_Reviewer_Comments_Revision_1

giaa073_Response_to_Reviewer_Comments_Revision_2

giaa073_Reviewer_1_Report_Original_Submission Rodrigo Baptista, Ph.D. -- 1/19/2020 Reviewed

giaa073_Reviewer_1_Report_Revision_1 Rodrigo Baptista, Ph.D. -- 4/13/2020 Reviewed

giaa073_Reviewer_2_Report_Original_Submission James Wasmuth -- 2/3/2020 Reviewed

giaa073_Reviewer_2_Report_Revision_1 James Wasmuth -- 4/21/2020 Reviewed

giaa073_Supplemental_Files

## Abbreviations

ANOVA: analysis of variance; ATP: adenosine triphosphate; BLAST: Basic Local Alignment Search Tool; bp: base pairs; BUSCO: Benchmarking Universal Single-Copy Orthologs; CDS: coding sequence; ELISA: enzyme-linked immunosorbent assay; EST: expressed sequence tag; FPKM: fragments per kilobase of gene length per million reads mapped; GO: gene ontology; HSP: heat shock protein; kb: kilobase pairs; KEGG: Kyoto Encyclopedia of Genes and Genomes; LINE: long interspersed nuclear element; LTR: long terminal repeat; Mb: megabase pairs; mRNA: messenger RNA; NCBI: National Center for Biotechnology Information; NIH: National Institutes of Health; OG: Orthologous Group; OPF: orthologous protein family; PacBio: Pacific Biosciences; PDR: People's Democratic Republic; RNA-seq: RNA sequencing; SRA: Sequence Read Archive; TE: transposable element; tRNA: transfer RNA.

## Competing Interests

The authors declare that they have no competing interests.

## Funding

Sequencing of the genomes was supported by the “Sequencing the etiological agents of the Food-Borne Trematodiases” project (NIH—National Human Genome Research Institute award No. U54HG003079). Comparative genome analysis was funded by grants NIH—National Institute of Allergy and Infectious Diseases AI081803 and NIH—National Institute of General Medical Sciences GM097435 to M.M. Parasite material from Thailand was supported by Distinguished Research Professor Grant (W.M.), Thailand Research Fund (Grant No. DPG6280002).

## Authors' Contributions

1. Conceptualization: M.M., P.J.B.
2. Formal analysis: B.A.R., Y.J.C., S.N.M., H.J., J.M.
3. Funding acquisition: P.J.B., M.M.
4. Methodology: P.J.B., P.U.F., D.B., M.M.
5. Resources: M.M., T.A., H.S., T.H.L., P.N.D., W.M., D.B., P.U.F.
6. Visualization: B.A.R., Y.J.C.
7. Writing—original draft: B.A.R., Y.J.C., M.M.
8. Writing—review and editing: D.B., P.J.B., P.U.F., M.M.
