# Supplementary material for: Comparative genomics and transcriptomics of 4 Paragonimus species provide insights into lung fluke parasitism and pathogenesis
Source: Gigascience. 2020 Jul 20;9(7):giaa073. doi: 10.1093/gigascience/giaa073 (PMC7370270; doi:10.1093/gigascience/giaa073)
Supplement: giaa073_GIGA-D-19-00411_Revision_1 [file giaa073_giga-d-19-00411_revision_1.pdf]

GigaScience

Comparative genomics and transcriptomics of four Paragonimus species provide insights into lung fluke parasitism and pathogenesis

--Manuscript Draft--

**Manuscript Number:**	GIGA-D-19-00411R1
**Full Title:**	Comparative genomics and transcriptomics of four Paragonimus species provide insights into lung fluke parasitism and pathogenesis
**Article Type:**	Research
**Funding Information:**	National Institutes of Health - National Human Genome Research Institute (U54HG003079) — Dr. Makedonka Mitreva<br>National Institutes of Health - National Institute of Allergy and Infectious Diseases (AI081803) — Dr. Makedonka Mitreva<br>National Institutes of Health - National Institute of General Medical Sciences (GM097435) — Dr. Makedonka Mitreva<br>Thailand Research Fund (TH) - Distinguished Research Professor Grant (DPG6280002) — Dr. Wanchai Maleewong

Funding source	Recipient
National Institutes of Health - National Human Genome Research Institute (U54HG003079)	Dr. Makedonka Mitreva
National Institutes of Health - National Institute of Allergy and Infectious Diseases (AI081803)	Dr. Makedonka Mitreva
National Institutes of Health - National Institute of General Medical Sciences (GM097435)	Dr. Makedonka Mitreva
Thailand Research Fund (TH) - Distinguished Research Professor Grant (DPG6280002)	Dr. Wanchai Maleewong

Abstract:

Background

Paragonimus spp. (lung flukes) are among the most injurious food-borne helminths, infecting ~23 million people, (~293 million with infection risk). Paragonimiasis is acquired from infected undercooked crustaceans and primarily affects the lungs, but often causes lesions elsewhere including the brain. The disease is easily mistaken for tuberculosis due to similar pulmonary symptoms, and accordingly, diagnostics are in demand.

Results

We assembled, annotated and compared draft genomes of four prevalent and distinct *Paragonimus* species: *P. miyazakii*, *P. westermani*, *P. kellicotti* and *P. heterotremus*. Genomes ranged from 697 to 923 Mb, included 12,072 to 12,853 genes, and were 87% to 96% complete according to BUSCO. Orthologous group (OG) analysis spanning 21 species (lung, liver and blood flukes, additional platyhelminths and hosts) provided insights into lung fluke biology, including identifying 256 lung fluke-specific and conserved OGs enriched for iron acquisition, immune modulation and other parasite functions. Transcriptome analysis identified consistent adult-stage *Paragonimus* expression profiles, and 388 genes differentially expressed between stages in the host body cavities and tissues, enriched for functions including proteolysis, nutrient transport and iron acquisition. Previously identified *Paragonimus* diagnostic antigens were matched to genes, providing an opportunity to optimize and ensure pan- *Paragonimus* -reactivity for diagnostic assays.

Conclusions

We anticipate that these novel genomic and transcriptomic resources will be invaluable for future lung fluke research. This report represents a major contribution to ongoing trematode genome sequencing efforts and underpins future studies into the biology, evolution and pathogenesis of *Paragonimus* and related food-borne flukes.

**Corresponding Author:**	Makedonka Mitreva<br><br>UNITED STATES
**Corresponding Author Secondary Information:**	
**Corresponding Author's Institution:**	
**Corresponding Author's Secondary Institution:**	

Powered by Editorial Manager® and ProduXion Manager® from Aries Systems Corporation

**First Author:**	Bruce A Rosa
**First Author Secondary Information:**	
**Order of Authors:**	Bruce A Rosa
	Young-Jun Choi
	Samantha N McNulty
	Hyeim Jung
	John Martin
	Takeshi Agatsuma
	Hiromu Sugiyama
	Thanh Le Hoa
	Pham Ngoc Doanh
	Wanchai Maleewong
	David Blair
	Paul J. Brindley
	Peter U. Fischer
	Makedonka Mitreva
**Order of Authors Secondary Information:**	
**Response to Reviewers:**	Please see attached point-by-point response file (at the end of the PDF).
**Additional Information:**	
**Question**	**Response**
Are you submitting this manuscript to a special series or article collection?	No
**Experimental design and statistics**<br><br>Full details of the experimental design and statistical methods used should be given in the Methods section, as detailed in our Minimum Standards Reporting Checklist. Information essential to interpreting the data presented should be made available in the figure legends.<br><br>Have you included all the information requested in your manuscript?	Yes
**Resources**<br><br>A description of all resources used, including antibodies, cell lines, animals and software tools, with enough information to allow them to be uniquely identified, should be included in the	Yes

Methods section. Authors are strongly encouraged to cite Research Resource Identifiers (RRIDs) for antibodies, model organisms and tools, where possible.<br><br>Have you included the information requested as detailed in our Minimum Standards Reporting Checklist?	
**Availability of data and materials**<br><br>All datasets and code on which the conclusions of the paper rely must be either included in your submission or deposited in publicly available repositories (where available and ethically appropriate), referencing such data using a unique identifier in the references and in the “Availability of Data and Materials” section of your manuscript.<br><br>Have you have met the above requirement as detailed in our Minimum Standards Reporting Checklist?	Yes

Powered by Editorial Manager® and ProduXion Manager® from Aries Systems Corporation

Manuscript

Click here to access/download;Manuscript;Paragonimus_Genomes_REVISE

Comparative genomics and transcriptomics of four *Paragonimus* species provide insights into lung fluke parasitism and pathogenesis

Bruce A. Rosa[1]*, Young-Jun Choi[1]*, Samantha N. McNulty[2], Hyeim Jung[1], John Martin[1], Takeshi Agatsuma[3], Hiromu Sugiyama[4], Thanh Le Hoa[5], Pham Ngoc Doanh[6,7], Wanchai Maleewong[8], David Blair[9], Paul J. Brindley[10], Peter U. Fischer[1], Makedonka Mitreva[1,2†]

[1]Department of Internal Medicine, Washington University School of Medicine, St. Louis, MO 63110, USA

[2]The McDonnell Genome Institute at Washington University, School of Medicine, St. Louis, MO 63108, USA

[3]Department of Environmental Health Sciences, Kochi Medical School, Oko, Nankoku City, Kochi 783-8505, Japan

[4]Laboratory of Helminthology, Department of Parasitology, National Institute of Infectious Diseases, Tokyo 162-8640, Japan

[5]Department of Immunology, Institute of Biotechnology, Vietnam Academy of Science and Technology, Hanoi, Vietnam

[6]Institute of Ecology and Biological Resources, Vietnam Academy of Science and Technology, Hanoi, Vietnam

[7]Graduate University of Science and Technology, Vietnam Academy of Science and Technology, Hanoi, Vietnam

[8]Research and Diagnostic Center for Emerging Infectious Diseases, Khon Kaen University, Khon Kaen, Thailand, Department of Parasitology, Faculty of Medicine, Khon Kaen University, Khon Kaen, Thailand

[9]College of Marine and Environmental Sciences, James Cook University, Townsville, Queensland 4811, Australia

[10]Departments of Microbiology, Immunology and Tropical Medicine, and Research Center for Neglected Diseases of Poverty, and Pathology School of Medicine & Health Sciences, George Washington University, Washington, DC 20037, USA

*Authors contributed equally to this work

†Correspondence should be addressed to Makedonka Mitreva. Tel. +1-314-285-2005, Fax +1-314-286-1800, Email: mmitreva@wustl.edu

Emails:

Bruce A. Rosa: barosa@wustl.edu

Young-Jun Choi: choi.y@wustl.edu

Samantha N. McNulty: samantha.n.mcnulty@gmail.com

Hyeim Jung: jungh@wustl.edu

John Martin: jmartin@wustl.edu

Takeshi Agatsuma: agatsuma@kochi-u.ac.jp

Hiromu Sugiyama: hsugi@niid.go.jp

Thanh Le Hoa: imibtvn@gmail.com

Pham Ngoc Doanh: pndoanh@yahoo.com

Wanchai Maleewong: wanch_ma@kku.ac.th

David Blair: david.blair@jcu.edu.au

Paul J. Brindley: pbrindley@gwu.edu

Peter U. Fischer: pufischer@wustl.edu

Makedonka Mitreva: mmitreva@wustl.edu

Keywords

Lung flukes, genomics, transcriptomics, paragonimiasis, infectious disease, trematodes

Abstract

Background

Paragonimus spp. (lung flukes) are among the most injurious food-borne helminths, infecting ~23 million people, (~293 million with infection risk). Paragonimiasis is acquired from infected undercooked crustaceans and primarily affects the lungs, but often causes lesions elsewhere including the brain. The disease is easily mistaken for tuberculosis due to similar pulmonary symptoms, and accordingly, diagnostics are in demand.

Results

We assembled, annotated and compared draft genomes of four prevalent and distinct *Paragonimus* species: *P. miyazakii*, *P. westermani*, *P. kellicotti* and *P. heterotremus*. Genomes ranged from 697 to 923 Mb, included 12,072 to 12,853 genes, and were 87% to 96% complete according to BUSCO. Orthologous group (OG) analysis spanning 21 species (lung, liver and blood flukes, additional platyhelminths and hosts) provided insights into lung fluke biology, including identifying 256 lung fluke-specific and conserved OGs enriched for iron acquisition, immune modulation and other parasite functions. Transcriptome analysis identified consistent adult-stage *Paragonimus* expression profiles, and 388 genes differentially expressed between stages in the host body cavities and tissues, enriched for functions including proteolysis, nutrient transport and iron acquisition. Previously identified *Paragonimus* diagnostic antigens were matched to genes, providing an opportunity to optimize and ensure pan-*Paragonimus*-reactivity for diagnostic assays.

Conclusions

We anticipate that these novel genomic and transcriptomic resources will be invaluable for future lung fluke research. This report represents a major contribution to ongoing trematode genome sequencing efforts and underpins future studies into the biology, evolution and pathogenesis of *Paragonimus* and related food-borne flukes.

Background

The trematode genus *Paragonimus*, the lung flukes, is among the most injurious taxon of food-borne helminths. About 23 million people are infected with lung flukes [1], an estimated 292 million people are at-risk, mainly in eastern Asia [2] , and billions of people live in areas where *Paragonimus* infections of animals are endemic. The life-cycle of *Paragonimus* species involves freshwater snails, crustacean intermediate hosts and mammals in Asia, parts of Africa, and the Americas [3]. Human paragonimiasis is acquired by consuming raw or undercooked shrimp and crabs containing the metacercaria, which is the infective stage. Although primarily affecting the lungs, lesions can occur at other sites, including the brain [4], and pulmonary paragonimiasis is frequently mistaken for tuberculosis due to similar respiratory symptoms [4].

Pathogenesis ensues because of the migration of the newly invading juveniles from the gut to the lungs and through not-infrequent ectopic migration to the brain, reproductive organs, and subcutaneous sites at the extremities, and because of toxins and other mediators released by the parasites during the larval migration [4, 5]. The presence of the flukes in the lung causes hemorrhage, inflammation with leukocytic infiltration and necrosis of lung parenchyma that gradually proceeds to the development of fibrotic encapsulation except for a fistula from the evolving lesion to the respiratory tract. Eggs of the lung fluke exit the encapsulated lesion through the fistula to reach the sputum and/or feces of the host, where they pass to the external environment, accomplishing transmission of the parasite [6]. There are signs and symptoms that allow characterization of acute and chronic stages of paragonimiasis. In pulmonary paragonimiasis, for example, the most noticeable clinical symptom of an infected individual is a chronic cough with gelatinous, rusty brown, pneumonia-like, blood-streaked sputum [6]. Heavy work commonly induces hemoptysis. Pneumothorax, empyema from secondary bacterial infection and pleural effusion might also be presented. When symptoms include only a chronic cough, the disease may be misinterpreted as chronic bronchitis and bronchiectasis or bronchial asthma. Pulmonary paragonimiasis is frequently confused with pulmonary tuberculosis [7]. The symptoms of extra-pulmonary paragonimiasis vary depending on the location of the fluke, including cerebral [5] and abdominal paragonimiasis [6].

Paragonimus is a large genus that includes more than 50 nominal species [8]. Seven of these species or species complexes of *Paragonimus* are known to infect humans [3]. This is also an ancient genus, thought to have originated before the breakup of Gondwana [9], but possibly also dispersing as colonists from the original East

Asian clade, based on the distribution of host species [10]. To improve our understanding of pathogens across this genus at the molecular level, we have assembled, annotated and compared draft genomes of four of these, three from Asia (*P. westermani* from Japan, *P. heterotremus, P. miyazakii*) and one from North America (*P. kellicotti*). Among them, *P. westermani* is the best-known species causing pulmonary paragonimiasis. This name has been applied to a genetically and geographically diverse complex of lung fluke populations differing widely in biological features including infectivity to humans [11]. The complex extends from India and Sri Lanka eastwards to Siberia, Korea and Japan, and southwards into Vietnam, Indonesia and the Philippines. However, human infections are reported primarily from China, Korea, Japan and the Philippines. Until this study, an Indian member of the *P. westermani* complex was the only lung fluke species for which a genome sequence was available [12]. *Paragonimus heterotremus* is the most common cause of pulmonary paragonimiasis in southern China, Lao PDR, Vietnam, northeastern India and Thailand [6, 8]. *Paragonimus miyazakii* is a member of the *P. skrjabini* complex, to which Blair and co-workers accorded sub-specific status [13]. Flukes of this complex tend not to mature in humans but frequently cause ectopic disease at diverse sites, including the brain. In North America, infection with *P. kellicotti* is primarily a disease of native, crayfish-eating mammals including the otter and mink. The occasional human infections can be severe, and thoracic involvement is typical [14, 15].

These four species represent a broad sampling of the phylogenetic diversity of the genus. Most of the known diversity, as revealed by DNA sequences from portions of the mitochondrial genome and the nuclear ribosomal genes, resides in Asia [16]. Analysis of the ITS2 marker by Blair et al [16] indicates that each of the species sequenced occupies a distinct clade within the phylogenetic tree.

In addition to a greater understanding of the genome contents of this group of food-borne trematodes, the findings presented here provide new information to assist development of diagnostic tools and recognition of potential drug targets. The findings will facilitate evolutionary, zoogeographical and phylogenetic investigation of the genus *Paragonimus* and its host-parasite relationships through the comparative analysis of gene content relative to other sequenced platyhelminth and host species, as well as through a comparative transcriptomic analysis.

Results and Discussion

Genome features

The sizes of the four novel *Paragonimus* genomes range from 697 to 923 Mb, containing between 12,072 and 12,853 genes. These draft genomes are estimated to be between 87% and 96% complete, according to BUSCO completeness estimates that include complete and fragmented eukaryote genes [17], with the new *P. westermani* genome produced from a sample collected from Japan being slightly more complete than the previously-sequenced genome produced from a sample collected from India [12] (96.4% vs 94.1%, respectively; **Table 1**). Here, statements about *P. westermani* apply to the new Japanese genome, unless otherwise stated. The total genome lengths of the *Paragonimus* spp. are larger than those of the Schistosomatidae and Opisthorchiidae, but smaller than those of Fasciolidae. However, the total numbers of protein-coding genes are comparable (**Table 1**). Repetitive sequences occupy between 49% and 54% of the *Paragonimus* genomes (**Figure 1A**). The repeat landscapes, depicting the relative abundance of repeat classes in the genome, versus the Kimura divergence from the consensus, revealed that *P. kellicotti* in particular has a significant number of copies of transposable elements (TE) with high similarity to consensus (Kimura substitution level: 0-5), indicating recent and current TE activity (**Figure 1B**). In a recent study [18], TE activity in the Fasciolidae was found to be low. TEs are potent sources of mutation that can rapidly create genetic variance, especially following genetic bottlenecks and environmental changes, providing bursts of allelic and phenotypic diversity upon which selection can act [19, 20]. Therefore, changes in TE activity, modulated by environmentally induced physiological or genomic stress, may have a major effect on adaptation of populations and species facing novel habitats and large environmental perturbations [21].

Focusing on the gene content, *P. kellicotti* had the shortest average total gene length among the species, and the lung flukes overall had similar gene lengths to other flukes, while platyhelminth species other than trematodes have shorter genes overall (**Figure 2A**). The variability in gene lengths observed between species results from differences in both average intron lengths (**Figure 2B**) and the average number of exons per gene (**Figure 2C**) while the average coding sequence (CDS) lengths of the exons across all the platyhelminth species were similar to each other (**Figure 2D**). Whereas there was species-to-species variability in gene lengths and exon counts, consistent patterns among the types of flukes were not apparent. Some of this variability may have

arisen due to the variation in quality of the assemblies, but these differences were minimized by only using complete gene models with a start and stop codon identified in the same frame.

Mitochondrial whole genome-based clustering was performed for the four *Paragonimus* species plus some additional existing mitochondrial genome assemblies for *P. ohirai* and four for *P. westermani*, including previously-sequenced mitochondrial genomes of *Paragonimus* (**Figure 3A**). This indicated that our Japanese *P. westermani* sample clustered with the existing known *P. westermani* samples from eastern Asia, and that all the other three newly sequenced species were distinct from *P. ohirai*.

We generated a PacBio long-read based mitochondrial assembly for *P. kellicotti*. The fully circularized complete genome was 17.3 kb in length, including a 3.7 kb non-coding repeat region between $tRNA^{Gly}$ and *cox3* (**Supplementary Figure S1**). There are seven copies of long repeats (378 bp) and 9.5 copies of short repeats (111 bp). The long repeats overlap with six copies of $tRNA^{Glu}$. This structural organization of repeat sequences does not resemble those found in *Paragonimus ohirai* [12] and *P. westermani* [12] where the non-coding region is partitioned by $tRNA^{Glu}$ into two parts.

Clustering of the four new lung fluke genomes, four liver fluke genomes, three blood fluke genomes, five other platyhelminth species, four host species and a yeast outgroup was performed based on the shared phylogeny among orthologous protein groups. These findings mirrored the mitochondrial clustering results for the lung fluke species (**Figure 3B**), indicating that *P. westermani* is the earlier-diverging taxon, as previously suggested based on ribosomal RNA [22].

Although our *P. westermani* reference genome was assembled using samples collected from Japan (Amakusa, Kyusyu). We compared the genomic sequences of our East Asian *P. westermani* to the recently published *P. westermani* genome from India (Changlang, Arunachal Pradesh) [12] to estimate the genetic divergence between geographically diverse samples. This analysis identified an average nucleotide sequence identity of 87.6%.

<u>Gene-family dynamics identify expanded functions distinguishing lung fluke species</u>

We investigated large-scale differences in gene complements among families of digenetic trematodes (**Figure 4A**) and modeled gene gain and loss while accounting for the phylogenetic history of species [23]. Gene families of interest that displayed pronounced differential expansion or contraction (**Figure 4B**) included the

papain-family cysteine proteases, cathepsins L, B and F, dynein heavy chain, spectrin/dystrophin, heat shock 70 kDa protein, major vault protein, and multidrug resistance protein. Total protease and protease inhibitor counts are shown in **Figure 4C**.

Lineage-specific expansion was observed in cathepsin F genes in *Paragonimus* spp. *Paragonimus miyazakii* RNA-seq reads showed that nine cathepsin F genes (out of 24 total) were differentially expressed, with expression levels in (peritoneal and pleural) cavity stage parasites significantly higher than in the tissue (lung and liver) developmental stages. Gene expression levels for each gene are provided in **Supplementary Table S2**. This suggested that (1) these enzymes are highly expressed during parasite penetration of the intestinal wall and invasion and migration through the abdominal and thoracic cavities (1- to 7-week-old immature stages), and (2) they might participate in metacercarial excystment, tissue invasion/migration and immune evasion. The remaining 15 constitutively expressed cathepsin F genes may have roles in nutrient digestion and remodeling of other physiologically active molecules. Ahn et al. [24] also reported differential expression of cathepsin F genes during development of *P. westermani*, and showed that most are highly immunogenic. This flagged them as prospective diagnostic targets. The importance of cathepsin F for *Paragonimus* contrasts with its function in the fasciolids, where cathepsin L genes are expanded and are thought to play a more critical role in host invasion [18, 25].

Differential expansion of cytoskeletal molecules is of interest in the context of tegument physiology [26]. Dynein is a microtubule motor protein, which transports intracellular cargo. Spectrin is an actin-binding protein, with a key role in maintenance of integrity of the plasma membrane. Dystrophin links microfilaments with extracellular matrix. The syncytial tegument of the surface of flatworms is a complex structure and a major adaptation to parasitism, and plays critical roles in nutrient uptake, immune response modulation and evasion, and other processes [26].

In *Paragonimus* spp., expanded gene families included heat shock proteins (HSPs), major vault proteins, and multidrug resistance proteins that play roles in maintaining cellular homeostasis under stress conditions. HSPs of flatworm parasites play a key role as molecular chaperones in the maintenance of protein homeostasis. They also are immunogenic and immunomodulatory. HSP is the most abundant family of proteins in the immature and mature egg of *Schistosoma mansoni*, and in the miracidium [27] and is highly abundant in the tegument of the adult schistosome [28]. In addition, HSP is abundant in the excretory/secretory products of the adult

Schistosoma japonicum blood fluke [29]. HSP stimulates diverse immune cells, eliciting release of pro- and anti-inflammatory cytokines [30], binds human LDL (the purpose of which is unknown but may be associated with transport of apoprotein B or in lipid trafficking [31]) and, given these properties, HSP represents a promising vaccine and diagnostic candidate [32]. Vaults, ribonucleoprotein complexes, are highly conserved in eukaryotes. Although their exact function remains unclear, it may be associated with multidrug resistance phenotypes and with signal transduction. In *S. mansoni*, up-regulation of major vault protein has been observed during the transition from cercaria to schistosomulum and in praziquantel-resistant adult worms [33]. ATP-binding cassette transporters (ABC transporters) are essential components of cellular physiological machinery, and some ABC transporters, including P-glycoproteins, pump toxins and xenobiotics out of the cell. Overexpression of P-glycoprotein has been reported in a praziquantel-resistant *S. mansoni* [34].

Tetraspanin sequence evolution in *P. kellicotti*

We searched for genes that evolved under positive selection in the four *Paragonimus* spp. based on the non-synonymous to synonymous substitution rate ratio (d_N/d_S). We conducted the branch-site test of positive selection to identify adaptive gene variants that became fixed in each species [35] (**Supplementary Table S3**). A tetraspanin from *P. kellicotti* (PKEL_00573) reached statistical significance after correction for multiple testing (d_N/d_S = 9.9, FDR = 0.018). Tetraspanins are small integral proteins bearing four transmembrane domains which form two extracellular loops [36]. In trematodes, they are major components of the tegument at the host-parasite interface [37], are highly immunogenic vaccine antigens [38, 39], and may play a role in immune evasion [40]. In the tetraspanin sequence of *P. kellicotti*, we detected six amino acid sites under positive selection (**Supplementary Figure S2**). Five of the six sites were predicted to be located within the extracellular loops believed to interact with the immune system of the host. A similar pattern of positive selection within regions that code for extracellular loops has been reported in tetraspanin-23 from African *Schistosoma* species [41].

Gene phylogeny analysis identifies functions conserved and specific to fluke groups

We classified orthologous groups (OGs) based on phelogenetic distribution of proteins from each of the 21 species (**Figure 3B)**. Complete gene counts and lists per species and per OG are provided in **Supplementary Table S4**. These results were parsed to identify the OGs containing members among the platyhelminth species,

and those that were conserved across all members of each group (lung, liver, and blood flukes, and other platyhelminth species (**Figure 5A**). This analysis identified 256 OGs that were conserved among, and exclusive to, the lung flukes (**Figures 5A and 5B**). The lung fluke-conserved and -specific genes were significantly enriched for several gene ontology (GO) terms (**Table 2**; using *P. miyazakii* genes to test significance), most of which were related to peptidase activity (including serine proteases which are involved in host tissue invasion, anticoagulation, and immune evasion [42]), as well as “iron binding” (which may be related to novel iron acquisition mechanisms from host tissue, which is not well understood in most metazoan parasites, but has been described in schistosomes [43]).

Expansion of unique aspartic proteases (including those predicted to be retropepsins) and other peptidases in the lung flukes may be associated with digestion of ingested blood, given the key role of this category of hydrolases and their inhibitors in nutrition and digestion of hemoglobin by schistosomes, and indeed other blood-feeding worms including hookworms [44, 45]. Given that pulmonary hemorrhage and hemoptysis are cardinal signs of lung fluke infection, it can be anticipated that the lung flukes ingest host blood when localized at the ulcerous lesion induced in the pulmonary parenchyma by infection. Overall, protease counts across species were similar (**Figure 4C**) although *P. kellicotti* had substantially fewer protease inhibitors compared to the other *Paragonimus* species (34 vs 57, 62 and 66), *F. hepatica* (61) and *S. mansoni* (55). Protease inhibitors in flukes are thought to be important for creating a safe environment for the parasite inside the host by inhibiting and regulating protease activity and immunomodulation [91], so this may suggest a novel host interaction strategy by *P. kellicotti*.

Analysis of the adult-stage gene expression levels of the discrete protease classes (**Supplementary Figure S3**) did not identify substantial differences among the *Paragonimus* species, except for a lower expression of threonine proteases in *P. kellicotti.* During the adult stage, cysteine proteases in all *Paragonimus* species exhibited significantly higher expression overall compared to *F. hepatica*, but similar expression levels to *S. mansoni.* A previous study identified immunodominant excretory-secretory cysteine proteases of adult *Paragonimus westermani* involved in immune evasion [46] and another study identified critical roles for excretory-secretory cysteine proteases during tissue invasion by newly excysted metacercariae of *P. westermani* [47]. The rapid diversification and critical host-interaction functions of the proteases highlights their importance, both in terms of understanding *Paragonimus* biology and in terms of identifying targets for control.

Functional enrichment analysis among the lung, liver and blood fluke conserved-and-exclusive OGs (**Figure 5C**) indicated that each family of fluke has evolved a distinct set of aspartic peptidases, trematode eggshell synthesis genes and saposin-like genes (which interact with lipids and are strongly immunogenic during fascioliasis [48]). The lung flukes, meanwhile, have uniquely expanded sets of serine proteases, as well as other genes families with functions including FAR1 DNA binding (a class of proteins which are important secreted host-interacting proteins in some parasitic nematodes [49]), fatty-acid binding, and ferritin-like functions (intracellular proteins involved in iron metabolism, localized in vitelline follicles and eggs [50]).

Gene expression analysis identifies stage-specific lung fluke functions

Lung (adult) stage RNA-Seq datasets were collected for each of the four lung fluke species (accessions in **Supplementary Table S1**), and reads were mapped to each of their respective genomes. Based on the 1:1 gene orthologs (as defined by the previously described OG dataset), the orthologous genes across the lung flukes had consistent adult-stage gene expression levels, with Pearson correlations ranging from 0.72 to 0.85 (**Figure 6A**, **6B**). Worms from additional life cycle stages were collected for *P. miyazakii*, including samples sequenced from cavities (peritoneal and pleural cavities) and tissues (lung and liver). Based on gene expression profiles across all genes, the cavity samples clustered and correlated more closely with each other than with the peritoneal samples (and vice versa; **Supplementary Figure S1**). Differential expression analysis comparing tissue and cavity stages identified 216 genes significantly overexpressed in the cavities relative to the tissues, and 172 genes significantly overexpressed in the tissues relative to the cavities (**Figure 6C**). Functional enrichment among these gene sets (**Table 3**) indicates that within the cavities, *P. miyazakii* overexpresses genes related to cysteine peptidase activity (critical for larval migration through host tissues [51]), iron ion binding (related to oxygen scavenging), and sulfotransferase (responsible for anthelmintic resistance in *S. mansoni* [52]). Within the tissues, *P. miyazakii* overexpresses genes related to cytoskeleton and microtubules, lyases and phosphatases, carbon-oxygen lyase and ribonucleotide binding.

The *P. miyazakii* genes belonging to the lung fluke-conserved and -exclusive OGs (described above) on average had significantly higher expression levels in the liver stage compared to the pleural cavity and lung stages, and significantly lower expression in the lung stage compared to all of the other stages. These results suggest that most of these OGs contain genes that are actively expressed during the transit through the host

mammal, en route to the lungs (**Figure 6D**), although some were more highly expressed in the lung stage (**Table 4**), and these genes had annotated functions including serine and aspartic peptidases and an MFS transporter gene (transports nutrients and ions between cells and the environment [53]). However, to confirm these gene expression patterns for specific larval stages, followup studies with additional biological replicates are needed. Gene expression levels and orthologous group identifiers for each gene in each of the four species are provided in **Supplementary Table S2**, along with detailed functional annotations for each of the *P. miyazakii* genes.

This stage-specific gene expression offers insight into known and novel biological functions of lung flukes at different developmental stages and within different organs and tissues of the mammalian host and represents a sophisticated new resource for study of specific genes of the lung fluke.

<u>Treatments, vaccine targets and diagnostics</u>

The World Health Organization (WHO) currently recommends the use of praziquantel or, as a backup, triclabendazole for the treatment of paragonimiasis; both are highly effective for curing infections [54]. However, there are concerns about the development of resistance to these drugs; triclabendazole resistance of *P. westermani* was reported in a human case from Korea [55]. Furthermore, there is widespread resistance to triclabendazole in liver flukes in cattle in Australia and South America [56], and praziquantel resistance is anticipated in the future due to its widespread use as a single treatment for schistosomiasis, a worrisome situation which has encouraged the search for novel drugs [57]. The comparative analysis presented here identifies valuable putative protein targets for drug development, including *Paragonimus*-specific proteins and trematode-conserved proteins which do not share orthology to human proteins. The protein annotation data available in **Supplementary Table S2** also will enable prioritization including biological functional annotations [58, 59], protein weight and pi predictions [60], predictions of signal peptides and transmembrane domains [61] and cellular compartment localization [58], and sequence similarity matches to targets in the CHEMBL database [62]. This information can provide a starting point for future bioinformatic prioritization and drug testing (**Supplementary Tables S2** and **S3**).

Vaccination to prevent future infections would offer an attractive alternative to treatment, but development of vaccine protection against trematode infection has so far been unsuccessful and is unlikely to be practical for

paragonimiasis in the near future [63]. However, the complete genome sequences and comparative analysis of the gene sets presented here provide valuable resources for future vaccine target development.

Pulmonary paragonimiasis is frequently mistaken for tuberculosis or pneumonia, and often patients do not shed eggs, which leads to false positive diagnoses of other conditions such as malaria or pneumonia [4, 64, 65]. This highlights a pressing need for accurate, rapid and affordable diagnostic approaches for paragonimiasis, a topic which has been the focus of numerous reports. We performed BLAST sequence similarity searches of previously identified *Paragonimus* diagnostic antigen targets among the four species (**Supplementary Figure S5**). These included: (i) *P. westermani* and *P. pseudoheterotremus* cysteine proteases identified in two previous studies [66, 67] (matching to the same protein targets from both studies in *P. heterotremus* and *P. kellicotti*), one of which had high adult-stage expression levels in all four species [66]; (ii) three different tyrosine kinases (one of which was identified in two different studies, in *Clonorchis sinensis* and in *P. westermani* [68, 69]), all of which had relatively low gene expression levels in adult stages; (iii) a previously unannotated *P. heterotremus* ELISA antigen [70] with low expression across life cycle stages, which we now annotate as a saposin protein (which we found to rapidly evolve among flukes [**Figure 5C**], and which is strongly immunogenic in fascioliasis [48]); (iv) eggshell proteins of *P. westermani* [71], for which we now provide full-length sequences. We observed that this gene was conserved across and specific to the lung flukes, with lower gene expression in the young adult stage (*P. heterotremus*), but higher expression in the adult stages of all species; (v) among serodiagnostic *P. kellicotti* antigens based on a transcriptome assembly and proteomic evidence [72], we identified the top 10 of the 25 prioritized transcripts that best matched between the transcript sequence and the newly annotated draft genome of *P. kellicotti.* Thereafter, the full-length gene sequence in *P. kellicotti* was employed to query the other species. Several of these were highly expressed in the adult stage of all four species, including one that is fluke specific (PKEL_05597). However, not all of these had high sequence conservation across all species, with two only having weak hits in *P. heterotremus* (PKEL_00171 and PKEL_01872).

As a result of this newly developed genomic resource for the lung flukes, previously identified diagnostic targets were identified with full gene sequences across all four species. The complete gene sequences, conservation information and transcriptomic gene expression data for these target proteins can allow for optimization of the targets for diagnostic testing that is effective on species spanning the genus (**Supplementary**

Figure S5). This is noteworthy given the absence of a standardized, commercially-available test for serodiagnosis for human paragonimiasis.

Conclusion

To substantially improve our understanding of the lung flukes at the molecular level, we sequenced, assembled, annotated and compared draft genomes of four species of *Paragonimus*, three from Asia (*P. miyazakii*, *P. westermani* from Japan, *P. heterotremus*) and one from North America (*P. kellicotti*), thereby providing novel and valuable genomic resources across these important parasites for the first time. We have utilized these new resources to compare and analyze phylogenies, to identify gene sets and biological functions associated with parasitism in lung flukes, and to contribute a key resource for future investigation into host-parasite interactions for these poorly-understood agents of neglected tropical disease. Our identification of previously prioritized *Paragonimus* diagnostic markers in each of the four lung fluke species revealed that the same protein targets were identified in multiple studies, and hence the availability of full gene sequences now should facilitate diagnostic assays aiming for reactivity across all species of lung fluke. Overall, the novel genomic and transcriptomic resources developed here will be invaluable for research on paragonimiasis, guiding experimental design and generation of novel hypotheses.

Methods

Parasite specimens

Samples of DNA and RNA of *Paragonimus westermani* were sourced in Japan. *Paragonimus heterotremus* (LC strain, Vietnam) were recovered from a cat experimentally infected with metacercariae from Lai Chau province, northern Vietnam (70% ethanol preserved; whole worm). *Paragonimus miyazakii* metacercariae were recovered from freshwater crabs (*Geothelphusa dehaani*), collected in Shizuoka Prefecture, central Japan [15], and were raised to adulthood in rats. DNA and RNA samples were prepared for each of the (pre-)adult flukes recovered from the lungs and from the pleural and peritoneal cavities of experimentally infected rats. *Paragonimus kellicotti* adult worms for genome sequencing were recovered from the lungs of Mongolian gerbils infected in the laboratory with metacercariae recovered from Missouri crayfish [73].

Genome sequencing, assembly and annotation

DNA and RNA samples were collected from adult-stage parasites of four distinct *Paragonimus* species: *P. miyazakii* (Japan), *P. heterotremus* (LC strain, Vietnam), *P. kellicotti* (Missouri, USA) and *Paragonimus westermani* (Japan). Illumina DNA sequencing produced fragments, 3kb- and 8kb-insert whole-genome shotgun libraries, and PacBio reads were generated for *P. kellicotti*. The sequences were generated on the Illumina platform and assembled using Allpaths_LG [74]. Scaffolding was improved using an in-house tool called Pygap (gap closure tool), the Pyramid assembler with Illumina paired reads to close gaps and extend contigs, and L_RNA_scaffolder [75] which uses transcript alignments to improve contiguity. For *P. kellicotti*, PacBio reads were assembled using PBJelly [76], utilizing the Illumina allpaths assembly as the reference. Nanocorr was used to perform error correction on the PacBio data. The nuclear genomes were annotated using the MAKER pipeline v2.31.8 [77]. Repetitive elements were softmasked with RepeatMasker v4.0.6 using a species-specific repeat library created by RepeatModeler v1.0.8, RepBase repeat libraries [78], and a list of known transposable elements provided by MAKER [77]. RNA-seq reads were aligned to their respective genome assemblies and assembled using StringTie v1.2.4 [79] (*P. miyazakii* samples collected from stages in the liver, peritoneal cavity [2 replicates], lung (adult) and pleural cavity; *P. heterotremus* samples from adults and young adults [2 replicates]; *P. westermani* [72] and *P. kellicotti* [80] adult-stage transcriptomic reads were retrieved from published reports). The resulting alignments and transcript assemblies were used by BRAKER [81] and MAKER pipelines, respectively, as extrinsic evidence. In addition, mRNA and EST sequences for each species were retrieved from NCBI, and were provided to MAKER as protein homology evidence along with protein sequences from UniRef100 [82] (Trematoda-specific, n=205,161) and WormBase ParaSite WBPS7 [83]. *Ab initio* gene predictions from BRAKER v2 [81] and AUGUSTUS v3.2.2 (trained by BRAKER and run within MAKER) were refined using the transcript and protein evidence. Previously unpredicted exons and UTRs were added, and split models were merged. The best-supported gene models were chosen based on Annotation Edit Distance (AED) [84]. To reduce false positives, gene predictions without supporting evidence were excluded in the final annotation build, with the exception of those encoding Pfam domains, as detected by InterProScan v5.19 [58]. These Pfam encoding domains were rescued in order to improve the annotation accuracy overall by balancing sensitivity and specificity [77, 85]. Gene products were named using PANNZER2 [86] and sma3s v2 [87]. **Supplementary Table S1** provides details of database accessions for the genomes. The completeness of

annotated gene sets was assessed using BUSCO v3.0, eukaryota_odb9 [17]. Gene Ontology (GO), KEGG and protease annotations were performed using InterProScan v5.19 [58], GhostKOALA [59], and MEROPS [88], respectively. ExPASy was used to perform protein weight and pi predictions [60], SignalP was used to predict predictions signal peptides and transmembrane domains [61], and gene product localization was predicted using the "cellular component" Gene Ontology annotations provided by InterProScan [58].

Functional enrichment testing was performed using GOSTATS [89] for GO enrichment and negative binomial distribution tests for InterPro domain enrichment (minimum 3 annotated genes required for significant enrichment). Ribosomal RNAs and tRNAs were annotated using RNAmmer v1.2.1 [90] and tRNAscan-SE v1.23 [91], respectively. Genome characteristics and statistics including CDS, numbers and lengths of genes, exons and introns were defined using the longest complete mRNA (with start and stop codon) for each gene. Across the four species of *Paragonimus*, complete mRNAs were found for an average of 86.2% of all annotated genes.

Assembly of the mitochondrial genome of *P. kellicotti* was achieved using CANU [92] to align PacBio long-reads, followed by error-correction using Pilon [93].

MUMmer v4.0 [94] was used to estimate the level of genetic divergence between *P. westermani* samples from Japan and India. Nucmerum was run first to generate genome alignments using draft assembly sequences. Dnadiff was then used to calculate the average sequence identity between the genomes considering only 1-to-1 alignments.

Transcriptome datasets and gene functional annotations

RNA-seq datasets were trimmed for adapters [95] and aligned [96] to their respective genome assemblies, and gene expression levels (FPKM) were quantified per gene per sample in each of the four species [97]. For *P. miyazakii*, differential gene expression analysis [98] identified genes significantly differentially expressed between the cavity and tissue samples. Interpro domains and Gene Ontology (GO) terms [58], KEGG enzymes [59], and protease [88] annotations of the genes were used to identify putative functions of genes of interest and perform pathway enrichment [89]. All raw RNA-Seq fastq files were uploaded to the NCBI Sequence Read Archive (SRA [99]), and complete sample metadata and accession information are provided in **Supplementary Table S1**. **Supplementary Table S2** provides, for each of the species, complete gene lists and

gene expression levels for each of the RNA-Seq samples. Complete functional annotations for every gene and the differential gene expression dataset are also provided for *P. miyazakii* in this table.

Repeat analysis

RepeatModeler v1.0.8 (with WU-BLAST as its search engine) was used to build, refine and classify consensus models of putative interspersed repeats for each species. With the resulting repeat libraries, genomic sequences were screened using RepeatMasker v4.0.6 in "slow search" mode to generate a detailed annotation of the interspersed and simple repeats. Per-copy distances to consensus were calculated (Kimura 2-parameter model, excluding CpG sites) and were plotted as repeat landscapes where divergence distribution reflected the activity of transposable elements (TE) on a relative time scale per genome using the calcDivergenceFromAlign.pl and createRepeatLandscape.pl scripts included in the RepeatMasker package.

Gene family evolution

Orthologous groups (OG) of genes of 21 species were inferred with OrthoFinder v1.1.4 [100] using the longest isoform for each gene (*Paragonimus* genome source information in **Supplementary Table S1**; Worm gene sets were retrieved from WormBase ParaSite in June 2017 [83]; Outgroup species gene sets were retrieved from Ensembl in June 2017 [101]). CAFE method [23] was employed to model gene gain and loss while accounting for the species' phylogenetic history based on an ultrametric species tree and the number of gene copies found in each species for each gene family. Birth-death (λ) parameters were estimated and the statistical significance of the observed family size differences among taxa were assessed. Results from OrthoFinder [100] were parsed to identify the OGs of interest based on conservation, including the lung fluke-conserved, liver fluke-conserved and blood fluke-conserved OGs and gene sets per species. **Supplementary Table S4** provides details of full OG counts per species and gene membership.

We used PosiGene [102] to search genome-wide for genes that evolved under positive selection based on the non-synonymous to synonymous substitution ratio. TMMOD [103] and Protter [104] were used for transmembrane helical topology prediction and visualization, respectively. We searched for genes that evolved under positive selection in the four *Paragonimus* spp. based on the non-synonymous to synonymous substitution

rate ratio (d_N/d_S). We conducted the branch-site test of positive selection to identify adaptive gene variants that became fixed in each species [35].

Previously identified *Paragonimus* diagnostic antigen search

Nucleotide sequences (or, if unavailable, amino acid sequences) were retrieved from each of the cited publications (**Supplementary Figure S5**). Diamond blastx (nucleotides; v0.9.9.110) or Diamond blastp (amino acids; v0.9.9.110) were used to identify the top hit gene in each *Paragonimus* genome annotation (default settings). The best BLAST E-value was used to identify the top match, followed by top bitscore, length and % ID in the case of ties. For the top 25 *P. kellicotti* immunodominant antigen transcripts identified in McNulty et al, 2014 [80], matches were identified between the assembled transcript and the annotated gene. For the other three species, the BLAST searches are performed against the identified *P. kellicotti* gene, and not the original transcript sequence.

RNAseq-based gene expression profiling

After adapter trimming using Trimmomatic v0.36 [95], RNA-seq reads were aligned to their respective genome assemblies using the STAR aligner [96] (2-pass mode, basic). All raw RNA-Seq fastq files were uploaded to the NCBI Sequence Read Archive (SRA [99]), and complete sample metadata and accession information are provided in **Supplementary Table S1**. Read fragments (read pairs or single reads) were quantified per gene per sample using featureCounts (version 1.5.1) [97]. FPKM (fragments per kilobase of gene length per million reads mapped) normalization was also performed. For *P. miyazakii*, significantly differentially expressed genes between the cavity and tissue sample sets were identified using DESeq2 (version 1.4.5) [98] with default settings, and a minimum *P*-value significance threshold of 0.05 (after False Discovery Rate [FDR [105]] correction for the number of tests). Pearson correlation-based RNA-Seq sample clustering was performed in R (using the hclust package, complete linkage).

Statistics

ANOVA analysis followed by Tukey's HSD post-hoc testing was performed to compare genome statistics and protease expression between species (**Figure 2**, **Supplementary Figure S3**). Because comparisons for the

genome statistics by *t* tests involved large numbers of values, which can falsely indicate positive statistical significance, a random selection of 100 values from each species was used (excluding the upper and lower 1% of data to avoid outliers). Letter labels above the species indicate statistical groups, i.e., if two species share the same letter then they were not statistically significant from each other.

Availability of supporting data and materials

Genomic raw reads, genome assemblies, genome annotations, and raw transcriptomic (RNA-Seq) fastq files were uploaded and are available for download from the NCBI Sequence Read Archive (SRA [99]), with all accession numbers and relevant metadata provided in **Supplementary Table S1**. **Supplementary Table S2** provides, for each of the species, complete gene lists and gene expression levels for each of the RNA-Seq samples. Complete functional annotations for every gene and the differential gene expression dataset are also provided for *P. miyazakii* in this table. All results of the genome-wide selection scan are provided in **Supplementary Table S3**. For each orthologous group identified, **Supplementary Table S4** provides complete gene lists, counts of genes per species, and average gene expression levels from each the *Paragonimus* transcriptome datasets described above. All relevant software versions, and commands specifying the parameters used are presented in **Supplementary Text S1**.

Declarations

<u>List of Abbreviations</u>

FPKM - Fragments Per Kilobase of gene length per Million reads mapped (gene expression level)

OG - Orthologous Group

TE – Transposable Elements

<u>Consent for Publication</u>

Not Applicable.

Competing Interests

The authors declare that they have no competing interests.

Funding

Sequencing of the genomes was supported by the ‘Sequencing the etiological agents of the Food-Borne Trematodiases’ project (National Institutes of Health - National Human Genome Research Institute award number U54HG003079). Comparative genome analysis was funded by grants National Institutes of Health - National Institute of Allergy and Infectious Diseases AI081803 and National Institutes of Health - National Institute of General Medical Sciences GM097435 to M.M. Parasite material from Thailand was supported by Distinguished Research Professor Grant (WM), Thailand Research Fund (Grant no. DPG6280002).

Author’s Contributions

1. **Conceptualization:** MM PJB.
2. **Formal analysis:** BAR YJC SNM HJ JM.
3. **Funding acquisition:** PJB MM.
4. **Methodology:** PJB PUF DB MM.
5. **Resources:** MM TA HS TLH PND WM DB PUF.
6. **Visualization:** BAR YJC.
7. **Writing – original draft:** BAR YJC MM.
8. **Writing – review & editing:** DB PJB PUF MM.

Acknowledgements

We gratefully acknowledge assistance provided by Xu Zhang and Kymberlie Pepin with genome assembly and annotation and by Rahul Tyagi for figure graphics. We thank Kurt Curtis for his help generating *P. kellicotti* parasite material.

References

1. Furst T, Keiser J and Utzinger J. Global burden of human food-borne trematodiasis: a systematic review and meta-analysis. Lancet Infect Dis. 2012;12 3:210-21. doi:10.1016/S1473-3099(11)70294-8.
2. Utzinger J, Becker SL, Knopp S, Blum J, Neumayr AL, Keiser J, et al. Neglected tropical diseases: diagnosis, clinical management, treatment and control. Swiss Med Wkly. 2012;142:w13727. doi:10.4414/smw.2012.13727.
3. Blair D. Paragonimiasis. Adv Exp Med Biol. 2014;766:115-52. doi:10.1007/978-1-4939-0915-5_5.
4. Furst T, Sayasone S, Odermatt P, Keiser J and Utzinger J. Manifestation, diagnosis, and management of foodborne trematodiasis. BMJ. 2012;344:e4093. doi:10.1136/bmj.e4093.
5. Lv S, Zhang Y, Steinmann P, Zhou XN and Utzinger J. Helminth infections of the central nervous system occurring in Southeast Asia and the Far East. Adv Parasitol. 2010;72:351-408. doi:S0065-308X(10)72012-1 [pii] 10.1016/S0065-308X(10)72012-1.
6. Sripa B, Kaewkes S, Intapan PM, Maleewong W and Brindley PJ. Food-borne trematodiases in Southeast Asia epidemiology, pathology, clinical manifestation and control. Adv Parasitol. 2010;72:305-50. doi:S0065-308X(10)72011-X [pii] 10.1016/S0065-308X(10)72011-X.
7. Liu Q, Wei F, Liu W, Yang S and Zhang X. Paragonimiasis: an important food-borne zoonosis in China. Trends Parasitol. 2008;24 7:318-23. doi:S1471-4922(08)00137-2 [pii] 10.1016/j.pt.2008.03.014.
8. Blair D, Xu ZB and Agatsuma T. Paragonimiasis and the genus Paragonimus. Adv Parasitol. 1999;42:113-222.
9. Blair D, Davis GM and Wu B. Evolutionary relationships between trematodes and snails emphasizing schistosomes and paragonimids. Parasitology. 2001;123:S229-S43. doi:Doi 10.1017/S003118200100837x.
10. Attwood SW, Upatham ES, Meng XH, Qiu DC and Southgate VR. The phylogeography of Asian Schistosoma (Trematoda: Schistosomatidae). Parasitology. 2002;125 Pt 2:99-112. doi:10.1017/s0031182002001981.
11. Doanh NP, Tu AL, Bui TD, Loan TH, Nonaka N, Horii Y, et al. Molecular and morphological variation of Paragonimus westermani in Vietnam with records of new second intermediate crab hosts and a new locality in a northern province. Parasitology. 2016;143 12:1639-46. doi:10.1017/S0031182016001219.
12. Oey H, Zakrzewski M, Narain K, Devi KR, Agatsuma T, Nawaratna S, et al. Whole-genome sequence of the oriental lung fluke Paragonimus westermani. Gigascience. 2019;8 1 doi:10.1093/gigascience/giy146.
13. Blair D, Chang Z, Chen M, Cui A, Wu B, Agatsuma T, et al. Paragonimus skrjabini Chen, 1959 (Digenea: Paragonimidae) and related species in eastern Asia: a combined molecular and morphological approach to identification and taxonomy. Syst Parasitol. 2005;60 1:1-21. doi:10.1007/s11230-004-1378-5.
14. Lane MA, Marcos LA, Onen NF, Demertzis LM, Hayes EV, Davila SZ, et al. Paragonimus kellicotti flukes in Missouri, USA. Emerg Infect Dis. 2012;18 8:1263-7. doi:10.3201/eid1808.120335.
15. Fischer PU and Weil GJ. North American paragonimiasis: epidemiology and diagnostic strategies. Expert Rev Anti-Infe. 2015;13 6:779-86. doi:10.1586/14787210.2015.1031745.
16. Blair D, Nawa Y, Mitreva M and Doanh PN. Gene diversity and genetic variation in lung flukes (genus Paragonimus). Trans R Soc Trop Med Hyg. 2016;110 1:6-12. doi:10.1093/trstmh/trv101.
17. Waterhouse RM, Seppey M, Simao FA, Manni M, Ioannidis P, Klioutchnikov G, et al. BUSCO applications from quality assessments to gene prediction and phylogenomics. Mol Biol Evol. 2017; doi:10.1093/molbev/msx319.
18. Choi YJ, Fontenla S, Fischer PU, Le TH, Costabile A, Blair D, et al. Adaptive Radiation of the Flukes of the Family Fasciolidae Inferred from Genome-Wide Comparisons of Key Species. Mol Biol Evol. 2020;37 1:84-99. doi:10.1093/molbev/msz204.
19. Stapley J, Santure AW and Dennis SR. Transposable elements as agents of rapid adaptation may explain the genetic paradox of invasive species. Mol Ecol. 2015;24 9:2241-52. doi:10.1111/mec.13089.
20. Schrader L and Schmitz J. The impact of transposable elements in adaptive evolution. Mol Ecol. 2018; doi:10.1111/mec.14794.

21. Chenais B, Caruso A, Hiard S and Casse N. The impact of transposable elements on eukaryotic genomes: from genome size increase to genetic adaptation to stressful environments. Gene. 2012;509 1:7-15. doi:10.1016/j.gene.2012.07.042.
22. Prasad PK, Tandon V, Biswal DK, Goswami LM and Chatterjee A. Phylogenetic reconstruction using secondary structures and sequence motifs of ITS2 rDNA of Paragonimus westermani (Kerbert, 1878) Braun, 1899 (Digenea: Paragonimidae) and related species. BMC Genomics. 2009;10 Suppl 3:S25. doi:10.1186/1471-2164-10-S3-S25.
23. Han MV, Thomas GW, Lugo-Martinez J and Hahn MW. Estimating gene gain and loss rates in the presence of error in genome assembly and annotation using CAFE 3. Mol Biol Evol. 2013;30 8:1987-97. doi:10.1093/molbev/mst100.
24. Ahn CS, Na BK, Chung DL, Kim JG, Kim JT and Kong Y. Expression characteristics and specific antibody reactivity of diverse cathepsin F members of Paragonimus westermani. Parasitol Int. 2015;64 1:37-42. doi:10.1016/j.parint.2014.09.012.
25. McNulty SN, Tort JF, Rinaldi G, Fischer K, Rosa BA, Smircich P, et al. Genomes of Fasciola hepatica from the Americas Reveal Colonization with Neorickettsia Endobacteria Related to the Agents of Potomac Horse and Human Sennetsu Fevers. PLoS Genet. 2017;13 1:e1006537. doi:10.1371/journal.pgen.1006537.
26. Jones MK, Gobert GN, Zhang L, Sunderland P and McManus DP. The cytoskeleton and motor proteins of human schistosomes and their roles in surface maintenance and host-parasite interactions. Bioessays. 2004;26 7:752-65. doi:10.1002/bies.20058.
27. Mathieson W and Wilson RA. A comparative proteomic study of the undeveloped and developed Schistosoma mansoni egg and its contents: the miracidium, hatch fluid and secretions. Int J Parasitol. 2010;40 5:617-28. doi:10.1016/j.ijpara.2009.10.014.
28. Sotillo J, Pearson M, Becker L, Mulvenna J and Loukas A. A quantitative proteomic analysis of the tegumental proteins from Schistosoma mansoni schistosomula reveals novel potential therapeutic targets. Int J Parasitol. 2015;45 8:505-16. doi:10.1016/j.ijpara.2015.03.004.
29. Liu F, Cui SJ, Hu W, Feng Z, Wang ZQ and Han ZG. Excretory/secretory proteome of the adult developmental stage of human blood fluke, Schistosoma japonicum. Mol Cell Proteomics. 2009;8 6:1236-51. doi:10.1074/mcp.M800538-MCP200.
30. Kolinski T, Marek-Trzonkowska N, Trzonkowski P and Siebert J. Heat shock proteins (HSPs) in the homeostasis of regulatory T cells (Tregs). Cent Eur J Immunol. 2016;41 3:317-23. doi:10.5114/ceji.2016.63133.
31. Pereira AS, Cavalcanti MG, Zingali RB, Lima-Filho JL and Chaves ME. Isoforms of Hsp70-binding human LDL in adult Schistosoma mansoni worms. Parasitol Res. 2015;114 3:1145-52. doi:10.1007/s00436-014-4292-z.
32. He S, Yang L, Lv Z, Hu W, Cao J, Wei J, et al. Molecular and functional characterization of a mortalin-like protein from Schistosoma japonicum (SjMLP/hsp70) as a member of the HSP70 family. Parasitol Res. 2010;107 4:955-66. doi:10.1007/s00436-010-1960-5.
33. Reis EV, Pereira RV, Gomes M, Jannotti-Passos LK, Baba EH, Coelho PM, et al. Characterisation of major vault protein during the life cycle of the human parasite Schistosoma mansoni. Parasitol Int. 2014;63 1:120-6. doi:10.1016/j.parint.2013.10.005.
34. Messerli SM, Kasinathan RS, Morgan W, Spranger S and Greenberg RM. Schistosoma mansoni P-glycoprotein levels increase in response to praziquantel exposure and correlate with reduced praziquantel susceptibility. Mol Biochem Parasitol. 2009;167 1:54-9. doi:10.1016/j.molbiopara.2009.04.007.
35. Yang Z. PAML 4: phylogenetic analysis by maximum likelihood. Mol Biol Evol. 2007;24 8:1586-91. doi:10.1093/molbev/msm088.
36. Huang S, Yuan S, Dong M, Su J, Yu C, Shen Y, et al. The phylogenetic analysis of tetraspanins projects the evolution of cell-cell interactions from unicellular to multicellular organisms. Genomics. 2005;86 6:674-84. doi:10.1016/j.ygeno.2005.08.004.
37. Chaiyadet S, Krueajampa W, Hipkaeo W, Plosan Y, Piratae S, Sotillo J, et al. Suppression of mRNAs encoding CD63 family tetraspanins from the carcinogenic liver fluke Opisthorchis viverrini results in distinct tegument phenotypes. Sci Rep. 2017;7 1:14342. doi:10.1038/s41598-017-13527-5.
38. Krautz-Peterson G, Debatis M, Tremblay JM, Oliveira SC, Da'dara AA, Skelly PJ, et al. Schistosoma mansoni Infection of Mice, Rats and Humans Elicits a Strong Antibody Response to a Limited Number

of Reduction-Sensitive Epitopes on Five Major Tegumental Membrane Proteins. PLoS Negl Trop Dis. 2017;11 1:e0005306. doi:10.1371/journal.pntd.0005306.
39. Tran MH, Pearson MS, Bethony JM, Smyth DJ, Jones MK, Duke M, et al. Tetraspanins on the surface of Schistosoma mansoni are protective antigens against schistosomiasis. Nat Med. 2006;12 7:835-40. doi:10.1038/nm1430.
40. Wu C, Cai P, Chang Q, Hao L, Peng S, Sun X, et al. Mapping the binding between the tetraspanin molecule (Sjc23) of Schistosoma japonicum and human non-immune IgG. PLoS One. 2011;6 4:e19112. doi:10.1371/journal.pone.0019112.
41. Sealey KL, Kirk RS, Walker AJ, Rollinson D and Lawton SP. Adaptive radiation within the vaccine target tetraspanin-23 across nine Schistosoma species from Africa. Int J Parasitol. 2013;43 1:95-103. doi:10.1016/j.ijpara.2012.11.007.
42. Yang Y, Wen Y, Cai YN, Vallee I, Boireau P, Liu MY, et al. Serine proteases of parasitic helminths. Korean J Parasitol. 2015;53 1:1-11. doi:10.3347/kjp.2015.53.1.1.
43. Glanfield A, McManus DP, Anderson GJ and Jones MK. Pumping iron: a potential target for novel therapeutics against schistosomes. Trends Parasitol. 2007;23 12:583-8. doi:10.1016/j.pt.2007.08.018.
44. Brindley PJ, Kalinna BH, Wong JY, Bogitsh BJ, King LT, Smyth DJ, et al. Proteolysis of human hemoglobin by schistosome cathepsin D. Mol Biochem Parasitol. 2001;112 1:103-12.
45. Williamson AL, Brindley PJ, Abbenante G, Prociv P, Berry C, Girdwood K, et al. Cleavage of hemoglobin by hookworm cathepsin D aspartic proteases and its potential contribution to host specificity. FASEB J. 2002;16 11:1458-60. doi:10.1096/fj.02-0181fje.
46. Lee EG, Na BK, Bae YA, Kim SH, Je EY, Ju JW, et al. Identification of immunodominant excretory-secretory cysteine proteases of adult Paragonimus westermani by proteome analysis. Proteomics. 2006;6 4:1290-300. doi:10.1002/pmic.200500399.
47. Na BK, Kim SH, Lee EG, Kim TS, Bae YA, Kang I, et al. Critical roles for excretory-secretory cysteine proteases during tissue invasion of Paragonimus westermani newly excysted metacercariae. Cell Microbiol. 2006;8 6:1034-46. doi:10.1111/j.1462-5822.2006.00685.x.
48. Caban-Hernandez K and Espino AM. Differential expression and localization of saposin-like protein 2 of Fasciola hepatica. Acta Trop. 2013;128 3:591-7. doi:10.1016/j.actatropica.2013.08.012.
49. Basavaraju SV, Zhan B, Kennedy MW, Liu Y, Hawdon J and Hotez PJ. Ac-FAR-1, a 20 kDa fatty acid- and retinol-binding protein secreted by adult Ancylostoma caninum hookworms: gene transcription pattern, ligand binding properties and structural characterisation. Mol Biochem Parasitol. 2003;126 1:63-71.
50. Jones MK, McManus DP, Sivadorai P, Glanfield A, Moertel L, Belli SI, et al. Tracking the fate of iron in early development of human blood flukes. Int J Biochem Cell Biol. 2007;39 9:1646-58. doi:10.1016/j.biocel.2007.04.017.
51. Grote A, Caffrey CR, Rebello KM, Smith D, Dalton JP and Lustigman S. Cysteine proteases during larval migration and development of helminths in their final host. PLoS Negl Trop Dis. 2018;12 8:e0005919. doi:10.1371/journal.pntd.0005919.
52. Taylor AB, Roberts KM, Cao X, Clark NE, Holloway SP, Donati E, et al. Structural and Enzymatic Insights into Species-specific Resistance to Schistosome Parasite Drug Therapy. 2017; doi:10.1074/jbc.M116.766527.
53. Pao SS, Paulsen IT and Saier MH, Jr. Major facilitator superfamily. Microbiol Mol Biol Rev. 1998;62 1:1-34.
54. World Health Organization. 2019. Accessed August 25, 2019.
55. Kyung SY, Cho YK, Kim YJ, Park JW, Jeong SH, Lee JI, et al. A paragonimiasis patient with allergic reaction to praziquantel and resistance to triclabendazole: successful treatment after desensitization to praziquantel. Korean J Parasitol. 2011;49 1:73-7. doi:10.3347/kjp.2011.49.1.73.
56. Kelley JM, Elliott TP, Beddoe T, Anderson G, Skuce P and Spithill TW. Current Threat of Triclabendazole Resistance in Fasciola hepatica. Trends Parasitol. 2016; doi:10.1016/j.pt.2016.03.002.
57. Mader P, Rennar GA, Ventura AMP, Grevelding CG and Schlitzer M. Chemotherapy for Fighting Schistosomiasis: Past, Present and Future. ChemMedChem. 2018;13 22:2374-89. doi:10.1002/cmdc.201800572.
58. Jones P, Binns D, Chang HY, Fraser M, Li W, McAnulla C, et al. InterProScan 5: genome-scale protein function classification. Bioinformatics. 2014;30 9:1236-40. doi:10.1093/bioinformatics/btu031 [pii].

59. Kanehisa M, Sato Y and Morishima K. BlastKOALA and GhostKOALA: KEGG Tools for Functional Characterization of Genome and Metagenome Sequences. J Mol Biol. 2016;428 4:726-31. doi:10.1016/j.jmb.2015.11.006.
60. Artimo P, Jonnalagedda M, Arnold K, Baratin D, Csardi G, de Castro E, et al. ExPASy: SIB bioinformatics resource portal. Nucleic Acids Res. 2012;40 Web Server issue:W597-603. doi:10.1093/nar/gks400.
61. Almagro Armenteros JJ, Tsirigos KD, Sonderby CK, Petersen TN, Winther O, Brunak S, et al. SignalP 5.0 improves signal peptide predictions using deep neural networks. Nat Biotechnol. 2019;37 4:420-3. doi:10.1038/s41587-019-0036-z.
62. Mendez D, Gaulton A, Bento AP, Chambers J, De Veij M, Felix E, et al. ChEMBL: towards direct deposition of bioassay data. Nucleic Acids Res. 2019;47 D1:D930-D40. doi:10.1093/nar/gky1075.
63. Stutzer C, Richards SA, Ferreira M, Baron S and Maritz-Olivier C. Metazoan Parasite Vaccines: Present Status and Future Prospects. Front Cell Infect Microbiol. 2018;8:67. doi:10.3389/fcimb.2018.00067.
64. Radzikowska E, Chabowski M and Bestry I. Tuberculosis mimicry. Eur Respir J. 2006;27 3:652; author reply doi:10.1183/09031936.06.00121205.
65. Eapen S, Espinal E and Firstenberg M. Delayed diagnosis of paragonimiasis in Southeast Asian immigrants: A need for global awareness. 2018;4 2:173-7. doi:10.4103/ijam.Ijam_2_18.
66. Yang SH, Park JO, Lee JH, Jeon BH, Kim WS, Kim SI, et al. Cloning and characterization of a new cysteine proteinase secreted by Paragonimus westermani adult worms. Am J Trop Med Hyg. 2004;71 1:87-92.
67. Yoonuan T, Nuamtanong S, Dekumyoy P, Phuphisut O and Adisakwattana P. Molecular and immunological characterization of cathepsin L-like cysteine protease of Paragonimus pseudoheterotremus. Parasitol Res. 2016;115 12:4457-70. doi:10.1007/s00436-016-5232-x.
68. Kim SH and Bae YA. Lineage-specific expansion and loss of tyrosinase genes across platyhelminths and their induction profiles in the carcinogenic oriental liver fluke, Clonorchis sinensis. Parasitology. 2017;144 10:1316-27. doi:10.1017/S003118201700083X.
69. Bae YA, Kim SH, Ahn CS, Kim JG and Kong Y. Molecular and biochemical characterization of Paragonimus westermani tyrosinase. Parasitology. 2015;142 6:807-15. doi:10.1017/S0031182014001942.
70. Pothong K, Komalamisra C, Kalambaheti T, Watthanakulpanich D, Yoshino TP and Dekumyoy P. ELISA based on a recombinant Paragonimus heterotremus protein for serodiagnosis of human paragonimiasis in Thailand. Parasit Vectors. 2018;11 1:322. doi:10.1186/s13071-018-2878-5.
71. Bae YA, Kim SH, Cai GB, Lee EG, Kim TS, Agatsuma T, et al. Differential expression of Paragonimus westermani eggshell proteins during the developmental stages. Int J Parasitol. 2007;37 3-4:295-305. doi:10.1016/j.ijpara.2006.10.006.
72. Li BW, McNulty SN, Rosa BA, Tyagi R, Zeng QR, Gu KZ, et al. Conservation and diversification of the transcriptomes of adult Paragonimus westermani and P. skrjabini. Parasit Vectors. 2016;9:497. doi:10.1186/s13071-016-1785-x.
73. Fischer PU, Curtis KC, Marcos LA and Weil GJ. Molecular characterization of the North American lung fluke Paragonimus kellicotti in Missouri and its development in Mongolian gerbils. Am J Trop Med Hyg. 2011;84 6:1005-11. doi:10.4269/ajtmh.2011.11-0027.
74. Gnerre S, Maccallum I, Przybylski D, Ribeiro FJ, Burton JN, Walker BJ, et al. High-quality draft assemblies of mammalian genomes from massively parallel sequence data. Proc Natl Acad Sci U S A. 2011;108 4:1513-8. doi:10.1073/pnas.1017351108.
75. Xue W, Li JT, Zhu YP, Hou GY, Kong XF, Kuang YY, et al. L_RNA_scaffolder: scaffolding genomes with transcripts. BMC Genomics. 2013;14:604. doi:10.1186/1471-2164-14-604.
76. English AC, Richards S, Han Y, Wang M, Vee V, Qu J, et al. Mind the gap: upgrading genomes with Pacific Biosciences RS long-read sequencing technology. PLoS One. 2012;7 11:e47768. doi:10.1371/journal.pone.0047768.
77. Holt C and Yandell M. MAKER2: an annotation pipeline and genome-database management tool for second-generation genome projects. BMC Bioinformatics. 2011;12:491. doi:10.1186/1471-2105-12-491.
78. Bao W, Kojima KK and Kohany O. Repbase Update, a database of repetitive elements in eukaryotic genomes. Mob DNA. 2015;6:11. doi:10.1186/s13100-015-0041-9.

79. Pertea M, Pertea GM, Antonescu CM, Chang TC, Mendell JT and Salzberg SL. StringTie enables improved reconstruction of a transcriptome from RNA-seq reads. Nat Biotechnol. 2015;33 3:290-5. doi:10.1038/nbt.3122.
80. McNulty SN, Fischer PU, Townsend RR, Curtis KC, Weil GJ and Mitreva M. Systems biology studies of adult paragonimus lung flukes facilitate the identification of immunodominant parasite antigens. PLoS Negl Trop Dis. 2014;8 10:e3242. doi:10.1371/journal.pntd.0003242.
81. Hoff KJ, Lange S, Lomsadze A, Borodovsky M and Stanke M. BRAKER1: Unsupervised RNA-Seq-Based Genome Annotation with GeneMark-ET and AUGUSTUS. Bioinformatics. 2016;32 5:767-9. doi:10.1093/bioinformatics/btv661.
82. The UniProt C. UniProt: the universal protein knowledgebase. Nucleic Acids Res. 2017;45 D1:D158-D69. doi:10.1093/nar/gkw1099.
83. Howe KL, Bolt BJ, Shafie M, Kersey P and Berriman M. WormBase ParaSite - a comprehensive resource for helminth genomics. Mol Biochem Parasitol. 2017;215:2-10. doi:10.1016/j.molbiopara.2016.11.005.
84. Eilbeck K, Moore B, Holt C and Yandell M. Quantitative measures for the management and comparison of annotated genomes. BMC Bioinformatics. 2009;10:67. doi:10.1186/1471-2105-10-67.
85. Campbell MS, Law M, Holt C, Stein JC, Moghe GD, Hufnagel DE, et al. MAKER-P: a tool kit for the rapid creation, management, and quality control of plant genome annotations. Plant Physiol. 2014;164 2:513-24. doi:10.1104/pp.113.230144.
86. Koskinen P, Toronen P, Nokso-Koivisto J and Holm L. PANNZER: high-throughput functional annotation of uncharacterized proteins in an error-prone environment. Bioinformatics. 2015;31 10:1544-52. doi:10.1093/bioinformatics/btu851.
87. Casimiro-Soriguer CS, Munoz-Merida A and Perez-Pulido AJ. Sma3s: A universal tool for easy functional annotation of proteomes and transcriptomes. Proteomics. 2017;17 12 doi:10.1002/pmic.201700071.
88. Rawlings ND, Barrett AJ and Finn R. Twenty years of the MEROPS database of proteolytic enzymes, their substrates and inhibitors. Nucleic Acids Res. 2016;44 D1:D343-50. doi:10.1093/nar/gkv1118.
89. Falcon S and Gentleman R. Using GOstats to test gene lists for GO term association. Bioinformatics. 2007;23 2:257-8. doi:10.1093/bioinformatics/btl567.
90. Lagesen K, Hallin P, Rodland EA, Staerfeldt HH, Rognes T and Ussery DW. RNAmmer: consistent and rapid annotation of ribosomal RNA genes. Nucleic Acids Res. 2007;35 9:3100-8. doi:10.1093/nar/gkm160.
91. Lowe TM and Eddy SR. tRNAscan-SE: a program for improved detection of transfer RNA genes in genomic sequence. Nucleic Acids Res. 1997;25 5:955-64.
92. Koren S, Walenz BP, Berlin K, Miller JR, Bergman NH and Phillippy AM. Canu: scalable and accurate long-read assembly via adaptive k-mer weighting and repeat separation. Genome Res. 2017;27 5:722-36. doi:10.1101/gr.215087.116.
93. Walker BJ, Abeel T, Shea T, Priest M, Abouelliel A, Sakthikumar S, et al. Pilon: an integrated tool for comprehensive microbial variant detection and genome assembly improvement. PLoS One. 2014;9 11:e112963. doi:10.1371/journal.pone.0112963.
94. Marcais G, Delcher AL, Phillippy AM, Coston R, Salzberg SL and Zimin A. MUMmer4: A fast and versatile genome alignment system. PLoS Comput Biol. 2018;14 1:e1005944. doi:10.1371/journal.pcbi.1005944.
95. Bolger AM, Lohse M and Usadel B. Trimmomatic: a flexible trimmer for Illumina sequence data. Bioinformatics. 2014;30 15:2114-20. doi:10.1093/bioinformatics/btu170.
96. Dobin A, Davis CA, Schlesinger F, Drenkow J, Zaleski C, Jha S, et al. STAR: ultrafast universal RNA-seq aligner. Bioinformatics. 2013;29 1:15-21. doi:10.1093/bioinformatics/bts635.
97. Liao Y, Smyth GK and Shi W. featureCounts: an efficient general purpose program for assigning sequence reads to genomic features. Bioinformatics. 2014;30 7:923-30. doi:10.1093/bioinformatics/btt656.
98. Anders S and Huber W. Differential expression analysis for sequence count data. Genome Biol. 2010;11 10:R106. doi:10.1186/gb-2010-11-10-r106.
99. Leinonen R, Sugawara H, Shumway M and on behalf of the International Nucleotide Sequence Database C. The Sequence Read Archive. Nucleic Acids Res. 2011;39 Database issue:D19-D21. doi:10.1093/nar/gkq1019.

100. Emms DM and Kelly S. OrthoFinder: solving fundamental biases in whole genome comparisons dramatically improves orthogroup inference accuracy. Genome Biol. 2015;16:157. doi:10.1186/s13059-015-0721-2.
101. Zerbino DR, Achuthan P, Akanni W, Amode MR, Barrell D, Bhai J, et al. Ensembl 2018. Nucleic Acids Res. 2018;46 D1:D754-D61. doi:10.1093/nar/gkx1098.
102. Sahm A, Bens M, Platzer M and Szafranski K. PosiGene: automated and easy-to-use pipeline for genome-wide detection of positively selected genes. Nucleic Acids Res. 2017;45 11:e100. doi:10.1093/nar/gkx179.
103. Kahsay RY, Gao G and Liao L. An improved hidden Markov model for transmembrane protein detection and topology prediction and its applications to complete genomes. Bioinformatics. 2005;21 9:1853-8. doi:10.1093/bioinformatics/bti303.
104. Omasits U, Ahrens CH, Muller S and Wollscheid B. Protter: interactive protein feature visualization and integration with experimental proteomic data. Bioinformatics. 2014;30 6:884-6. doi:10.1093/bioinformatics/btt607.
105. Benjamini Y and Hochberg Y. Controlling the False Discovery Rate: A Practical and Powerful Approach to Multiple Testing. Journal of the Royal Statistical Society Series B (Methodological). 1995;57 1:289-300. doi:10.2307/2346101.

Figure Captions

Figure 1. Comparisons of the overall content of the assembled *Paragonimus* genome assemblies. Comparisons are based on (**A**) length (including statistics for other sequenced trematode genomes) and (**B**) Repeat landscapes, measured using the Kimura substitution level, which indicates how much a repeat sequence has degenerated since its incorporation into the genome (i.e., how recently the repeat sequence was added). The high peak at the far left of *P. kellicotti* indicates a recent incorporation or active transposable element activity.

Figure 2: Comparison of genome annotation characteristics and attributes among several species of flatworms. Attributes characterized included (**A**) Full gene lengths, including coding and noncoding sequences, (**B**) Average intron lengths per gene, (**C**) Number of exons per gene, and (**D**) Coding sequence (CDS) length per exon. *P* values and letter groupings indicating significant differences among species, as calculated using ANOVA with Tukey's HSD post-hoc test.

Figure 3. Clustering of *Paragonimus* species. (**A**) Mitochondrial whole genome-based phylogeny, including previously-sequenced *Paragonimus* mitochondrial genomes (with accessions indicated). (**B**) Species clustering based on single-member OPF sequences. 262,720 genes (85% of all genes across the species) were assigned to 17,953 OPFs; 2,493 genes are in 326 species-specific OPFs.

Figure 4. Gene-family dynamics among platyhelminth species. (**A**) Rapidly evolving families of interest are quantified at each stage of the phylogeny, including genes gained (blue) and lost (red) relative to other species. The number of rapidly evolving genes are indicated in parentheses. (**B**) Functionally annotated gene families of interest that displayed most pronounced differential expansions or contractions. (**C**) Overall protease and protease inhibitor abundance per species.

Figure 5. Orthologous Group (OG) distribution analysis. (**A**) OGs identified among groups of flukes. The OGs conserved in at least one of the species from each group are indicated in black, and the OGs conserved among all the species in the overlapping groups are indicated in red. (**B**) Counts of OGs among the four *Paragonimus*

species, with *Paragonimus*-specific gene sets indicated in red text. The 256 *Paragonimus* conserved-and-specific genes are indicated with highlight (Table 4). (**C**) Significant functional enrichment (Interpro domains) among the gene sets conserved among, and specific to, each major group of flukes (256, 758 and 270 OPFs in lung, liver and blood flukes, respectively), relative to the functions in the complete gene sets.

Figure 6: Analysis of gene expression data for species of lung flukes of the genus *Paragonimus*. (**A**) Comparison of adult-stage gene expression levels among 1:1 orthologs shared by *P. westermani* and *P. miyazakii*. Pearson correlation = 0.79. (**B**) Pearson correlation values between all lung fluke species for the adult-stage expression levels of all 1:1 orthologous genes. (**C**) Differential gene expression between cavities (blue) and tissues (orange) in *P. miyazakii*. Clustering based on FPKM value across all genes is indicated in the bottom right (Pearson clustering, complete linkage). (**D**) A comparison of the average relative expression of the lung fluke-specific and -conserved genes in each *P. miyazakii* tissue type. ** P < 0.01, *** P < 0.001, according to an ANOVA test of all Z-score values.

Tables

Table 1: The draft genome of *Paragonimus*: assembly, size and annotation characteristics

Statistic	*Paragonimus miyazakii*	*Paragonimus heterotremus*	*Paragonimus kellicotti*	*Paragonimus westermani* (Japan)	*Paragonimus westermani* (India)
**Assembly statistics**					
Total genome length (Mb)	915.8	841.2	696.5	923.3	922.8
Number of contigs	22,318	27,557	29,377	22,477	30,455
Mean contig size (kb)	41	30.5	23.7	41.1	30.3
Median contig size (kb)	15.1	9.3	10.2	17.2	4.8
Max. contig size (kb)	919.8	715.6	826	829	809.4
N50 length (kb)	108.8	92.5	56.0	100.8	135.2
N50 number	2,320	2,506	3,316	2,664	1,943
**BUSCO completeness (303 genes, eukarota_odb9)**					
Complete, single copy	84.5%	82.5%	70.3%	88.78%	76.90%
Complete, duplicated	1.3%	0.0%	1.3%	1.32%	2.31%
Fragmented	7.6%	10.9%	15.2%	6.27%	14.85%
Missing	6.6%	6.6%	13.2%	3.63%	5.94%
Overall completeness	93.4%	93.4%	86.8%	96.37%	94.06%
**Gene statistics**					
Number of genes	12,652	12,490	12,853	12,072	12,771
Avg gene length (kb)	25.9	22.6	17.6	24.1	18.0
Avg CDS length (kb)	1.5	1.4	1.1	1.4	1.4
Avg intron length (kb)	4.2	4	3.6	4.2	4.0
Avg # exons per gene	6.7	6.2	5.3	6.3	5.2
% annotated InterPro	82%	85%	81%	87%	82%
% annotated KEGG	40%	41%	34%	43%	43%

Table 2. "Molecular Function" Gene Ontology terms enriched among *P. miyazakii* genes that are conserved among and exclusive to lung flukes.

GO ID	GO term name	P value	# Conserved and Specific	Total # in genome
GO:0004175	endopeptidase activity	5.2E-05	8	132
GO:0008236	serine-type peptidase activity	5.6E-05	6	67
GO:0017171	serine hydrolase activity	5.6E-05	6	67
GO:0004252	serine-type endopeptidase activity	1.6E-04	5	51
GO:0070011	peptidase activity, acting on L-amino acid peptides	6.1E-04	9	237
GO:0008233	peptidase activity	8.7E-04	9	249
GO:0004568	chitinase activity	2.1E-03	2	7
GO:0004190	aspartic-type endopeptidase activity	1.1E-02	2	16
GO:0070001	aspartic-type peptidase activity	1.1E-02	2	16
GO:0008199	ferric iron binding	1.1E-02	2	16

Table 3. "Molecular Function" Gene Ontology terms enriched among the 216 *P. miyazakii* genes that are overexpressed in cavities (peritoneal, pleural) relative to tissues (lung, liver), and among the 172 overexpressed in tissues relative to cavities.

**Sample group**	**GO term**	**Term**	**FDR-corrected P value**	**Number of genes over-expressed**
Cavities	GO:0008234	cysteine-type peptidase activity	1.0E-05	8
	GO:0005509	calcium ion binding	1.1E-05	13
	GO:0008233	peptidase activity	3.0E-04	11
	GO:0046872	metal ion binding	6.6E-04	17
	GO:0043169	cation binding	7.6E-04	17
	GO:0070011	peptidase activity, acting on L-amino acid peptides	8.3E-04	10
	GO:0008375	acetylglucosaminyltransferase activity	8.4E-04	3
	GO:0008194	UDP-glycosyltransferase activity	1.5E-03	3
	GO:0008146	sulfotransferase activity	2.4E-03	2
	GO:0016787	hydrolase activity	2.4E-03	21
	GO:0005544	calcium-dependent phospholipid binding	5.6E-03	2
	GO:0016782	transferase activity, transferring sulfur-containing groups	6.9E-03	2
	GO:0005506	iron ion binding	7.2E-03	3
Tissues	GO:0005200	structural constituent of cytoskeleton	1.1E-07	8
	GO:0016829	lyase activity	2.7E-04	6
	GO:0017111	nucleoside-triphosphatase activity	5.5E-04	14
	GO:0016462	pyrophosphatase activity	6.9E-04	14
	GO:0016818	hydrolase activity, acting on acid anhydrides…	7.6E-04	14
	GO:0004634	phosphopyruvate hydratase activity	8.2E-04	2
	GO:0016817	hydrolase activity, acting on acid anhydrides	8.3E-04	14
	GO:0003924	GTPase activity	1.6E-03	8
	GO:0003824	catalytic activity	2.0E-03	48
	GO:0016836	hydro-lyase activity	2.1E-03	3
	GO:0016835	carbon-oxygen lyase activity	2.6E-03	3
	GO:0003777	microtubule motor activity	3.1E-03	5
	GO:0016830	carbon-carbon lyase activity	3.6E-03	3
	GO:0016491	oxidoreductase activity	3.6E-03	10
	GO:0003774	motor activity	4.6E-03	5
	GO:0005198	structural molecule activity	6.6E-03	8
	GO:0032561	guanyl ribonucleotide binding	6.8E-03	8
	GO:0032550	purine ribonucleoside binding	6.8E-03	8
	GO:0001883	purine nucleoside binding	6.8E-03	8
	GO:0005525	GTP binding	6.8E-03	8
	GO:0032549	ribonucleoside binding	7.3E-03	8
	GO:0001882	nucleoside binding	7.6E-03	8
	GO:0019001	guanyl nucleotide binding	8.1E-03	8
	GO:0008017	microtubule binding	9.9E-03	4

Table 4: *Paragonimus*-conserved and -specific genes with relatively high expression levels in the *P. miyazakii* lung (adult) stage relative to other stages (minimum 1.5-fold expression difference compared to all other stages).

Gene	Gene function (InterPro)	Expression level (FPKM)			
		Peritoneal	Pleural	Liver	Lung
PMIY_10706	-	0	0	0	1.00
PMIY_04932	-	0	0	0	0.50
PMIY_04199	-	0	0	0	0.23
PMIY_05623	IPR009003: Peptidase S1, PA clan (Serine protease)	0	0	0	0.03
PMIY_05645	-	0.03	0	0	1.08
PMIY_10421	-	0	0	0	0.03
PMIY_10154	-	0.03	0.17	0.25	2.10
PMIY_10315	-	0.30	0.26	0	1.67
PMIY_07404	IPR028089: Domain of unknown function DUF4455	0.05	0	0	0.22
PMIY_06071	IPR035914: Spermadhesin, CUB domain superfamily	0.16	0.11	0.20	0.80
PMIY_09494	-	1.61	1.76	0.08	5.88
PMIY_01683	-	0.24	0.18	0.05	0.79
PMIY_12118	IPR021109: Aspartic peptidase domain superfamily	0.02	0	0	0.04
PMIY_06957	-	0.87	1.68	1.65	3.55
PMIY_00756	-	14.75	20.66	16.12	43.07
PMIY_01507	-	0.23	0.38	0.39	0.79
PMIY_11874	-	0.05	0.05	0	0.10
PMIY_05793	-	0.03	0.06	0.26	0.47
PMIY_05272	-	5.44	4.20	4.22	9.67
PMIY_12189	IPR036259: MFS transporter superfamily	1.84	1.72	1.73	3.25
PMIY_12491	-	0.63	0	0.18	1.08
PMIY_08946	-	0	0.06	0.00	0.10
PMIY_12111	-	1.23	0.35	1.04	2.04
PMIY_02371	IPR016024: Armadillo-type fold	10.30	9.68	12.40	20.06
PMIY_11682	-	4.36	6.64	5.66	10.58
PMIY_12247	IPR036236: Zinc finger C2H2 superfamily	2.84	3.22	4.16	6.60
PMIY_06566	-	0.63	1.02	1.04	1.64
PMIY_02942	-	5.32	5.71	8.45	13.11
PMIY_11404	-	2.40	2.82	2.58	4.36
PMIY_07606	IPR009060: UBA-like superfamily	1.33	0.12	0.49	2.03

Click here to access/download;Figure;Figure 1.tif

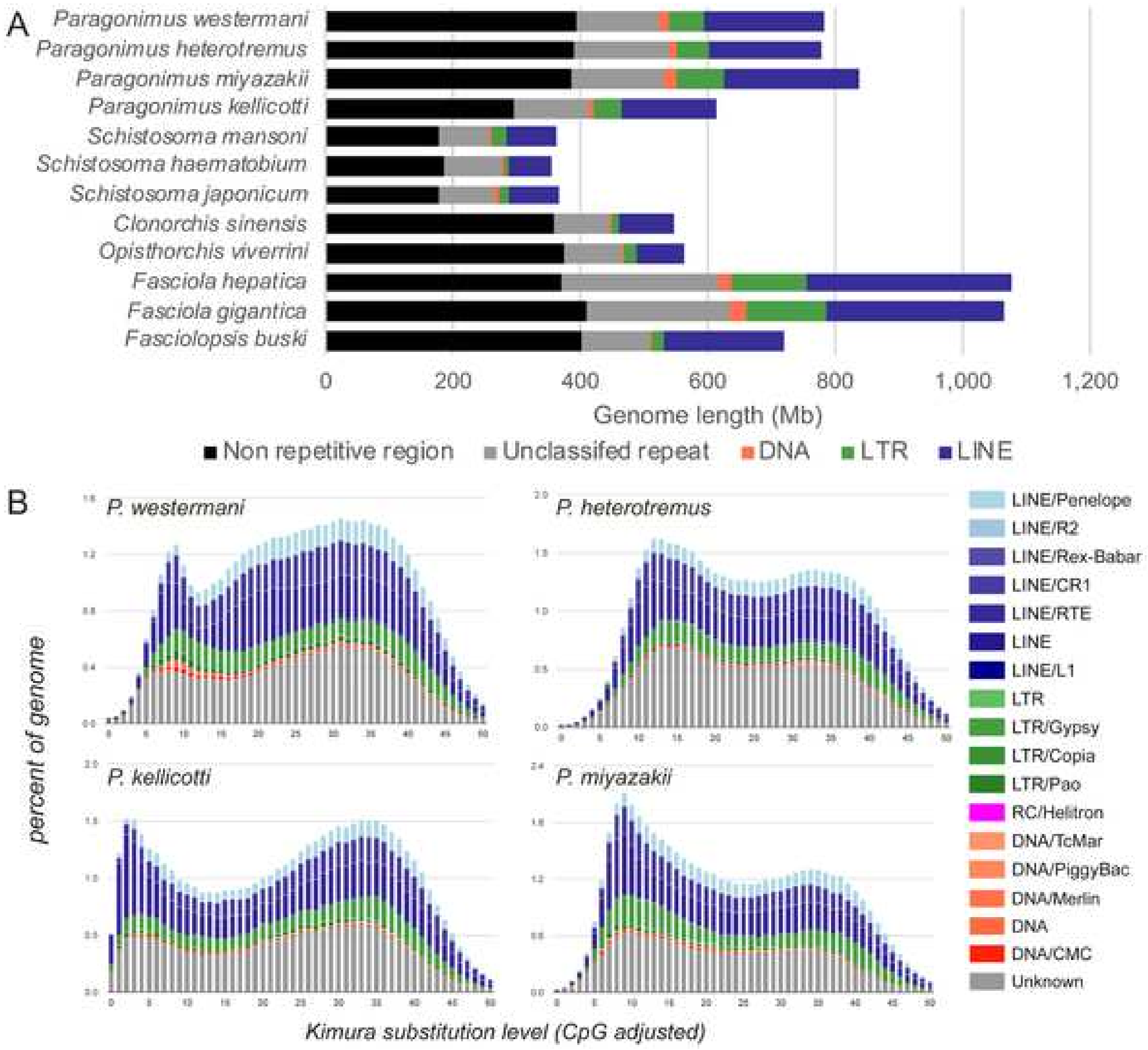

Figure 2

Click here to access/download;Figure;Figure 2.tif

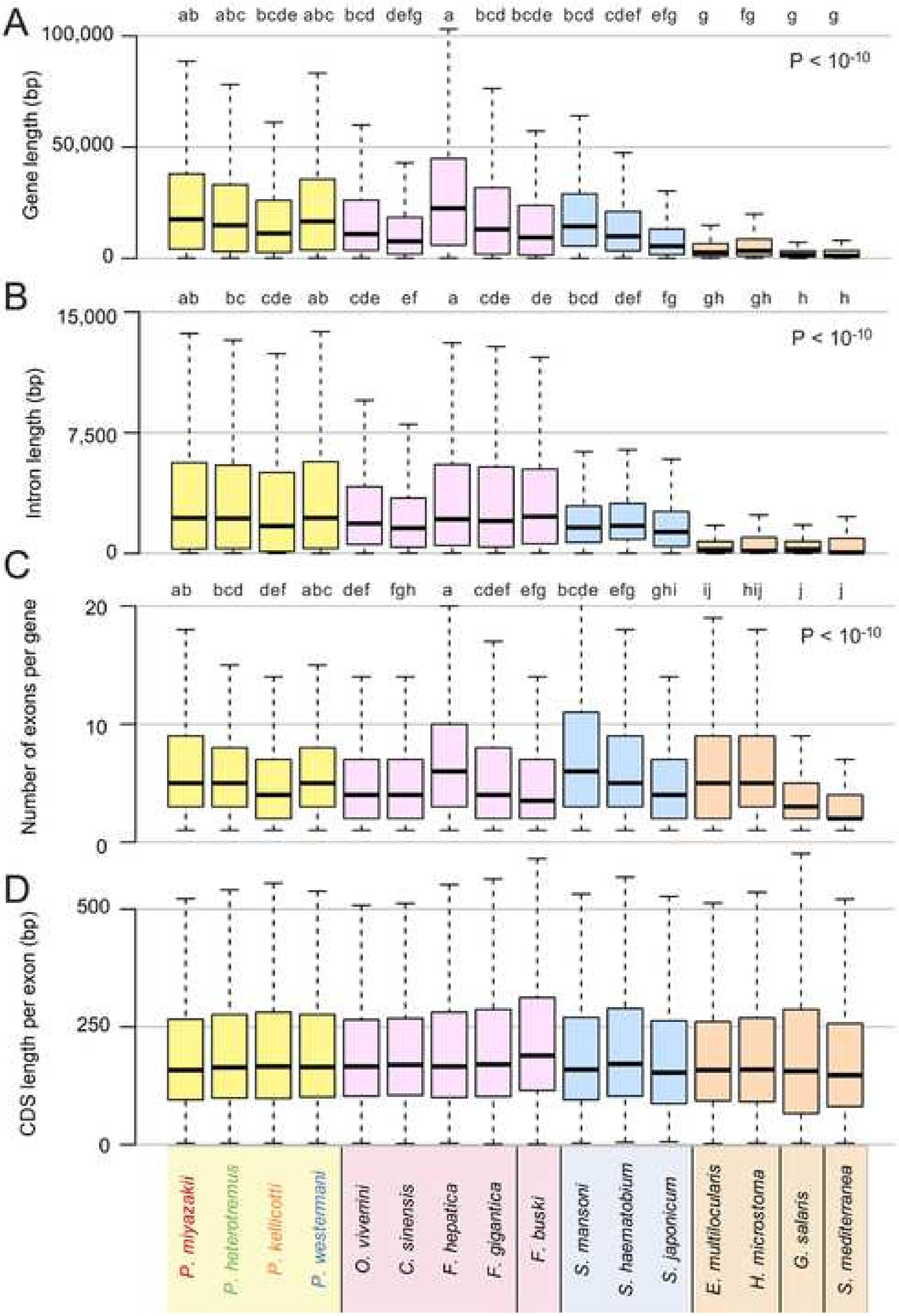

Figure 3

Click here to access/download;Figure;Figure 3.tif

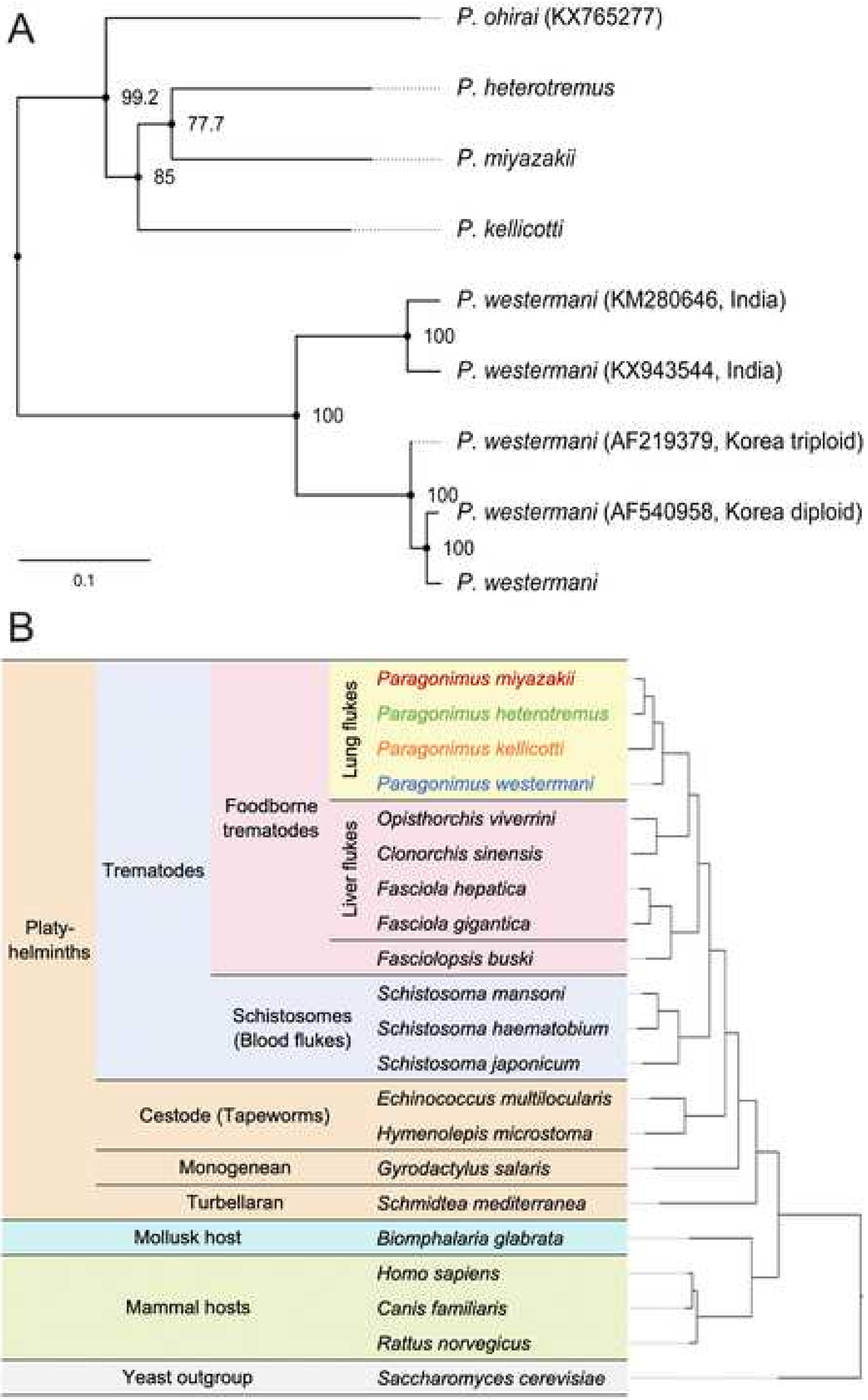

Figure 4
Click here to access/download;Figure;Figure 4.tif

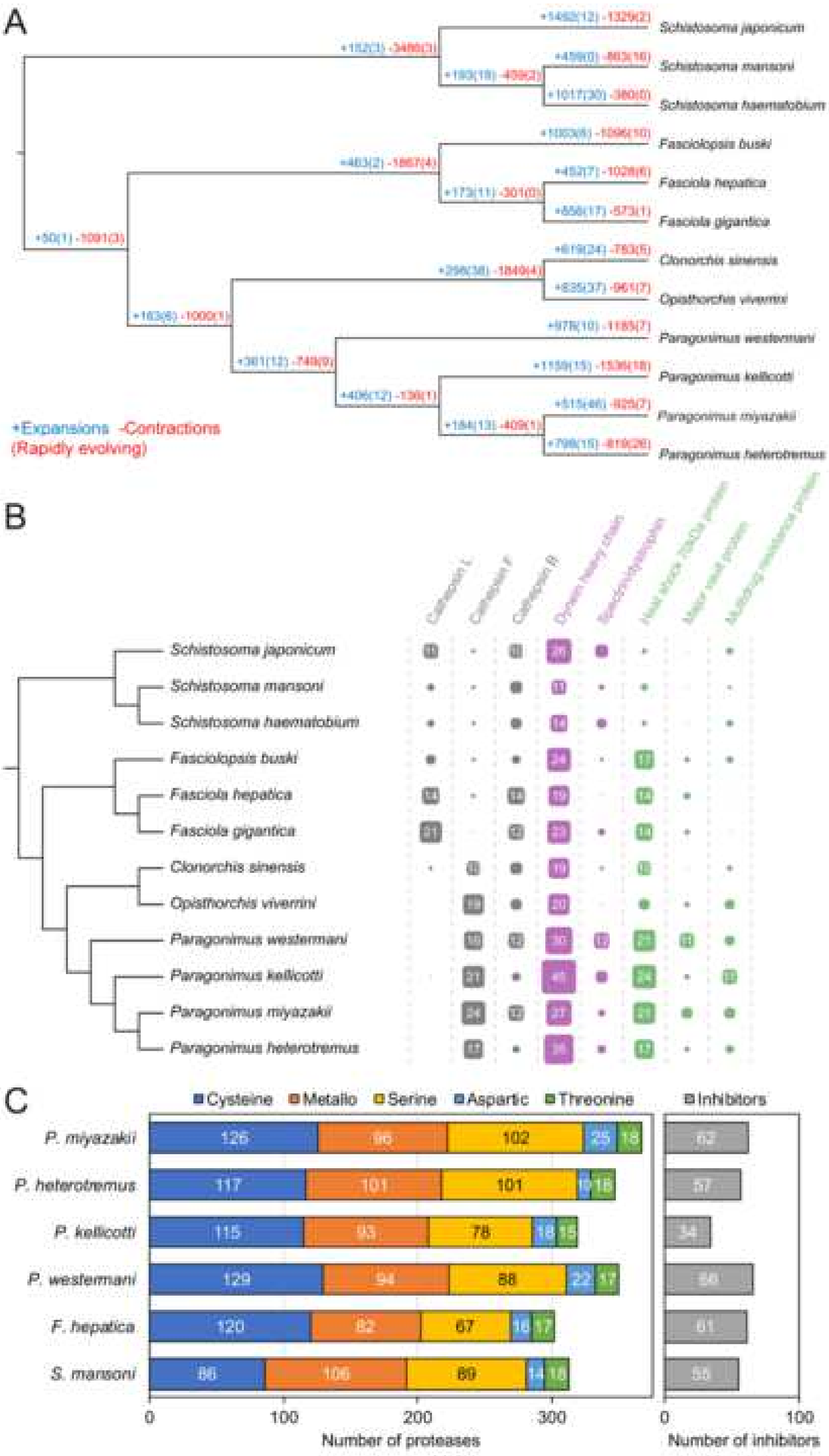

Figure 5

Click here to access/download;Figure;Figure 5.tif

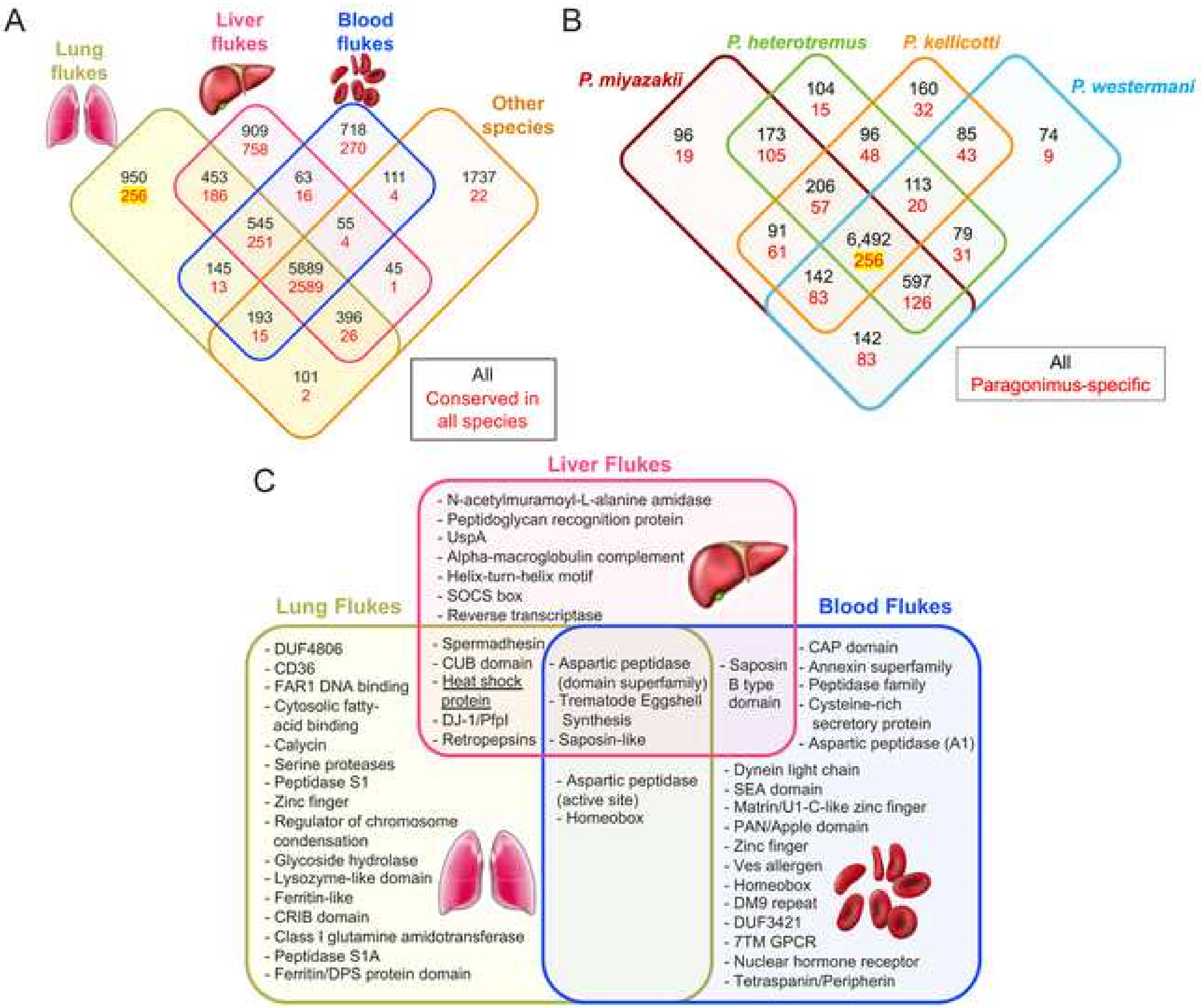

Figure 6

Click here to access/download;Figure;Figure 6.tif

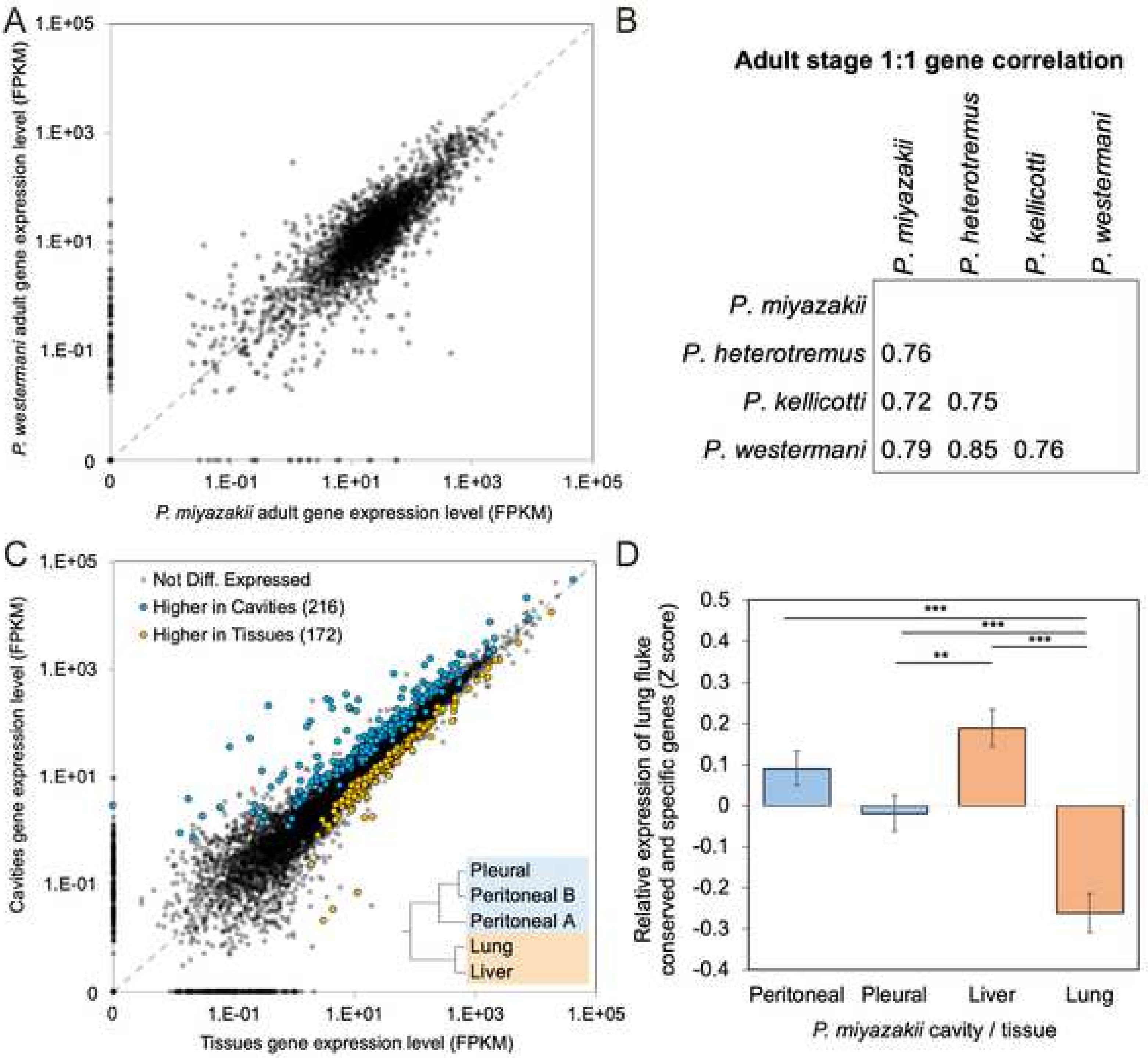

Click here to access/download
Supplementary Material
Supplementary Information (Combined) V5.docx

Supp Table S1

Click here to access/download

Supplementary Material

Supp Table S1 - Accessions.xlsx

Click here to access/download
Supplementary Material
Supp Table S2 - Paragonimus expression data per gene.xlsx

Supp Table S3

Click here to access/download
Supplementary Material
Supp Table S3 - Genome-wide selection scan.xlsx

Supp Table S4

Click here to access/download
Supplementary Material
Supp Table S4 - OGs and FPKM.xlsx

Cover Letter

Click here to access/download;Personal Cover;Gigascience Cover Letter Resubmission V2.docx

Washington University in St. Louis
SCHOOL OF MEDICINE

MCDONNELL GENOME INSTITUTE

To
Dr. Nicole Nogoy
Editor
Gigascience

3/18/2019

Re: **GIGA-D-19-00411** revision

Dear Dr. Nogoy,

Thank you for inviting us to submit a revised version of our manuscript: "*Comparative genomics and transcriptomics of four* Paragonimus *species provide insights into lung fluke parasitism and pathogenesis*" (GIGA-D-19-00411).

We appreciate the reviewer suggestions for improving the manuscript, and as our point-by-point response to the reviewer document shows, we have now comprehensively addressed all of the concerns and revised the manuscript in accordance with the reviewer's recommendations. We very much appreciate the efforts of the referee in recommending how to best revise this manuscript. We have followed their advice especially closely and are hopeful that the paper will now pass muster and we trust you will find it suitable for publication in Gigascience.

Thank you for your consideration.

Yours sincerely,

M. Mitreva

Makedonka Mitreva, PhD

Professor, Department of Medicine and of Genetics,
Assistant Director, McDonnell Genome Institute,
Director, Center for Clinical Genomics of Microbial Systems,
Washington University School of Medicine

The Elizabeth H. and James S. McDonnell III Genome Institute, Washington University, Campus Box 8501
4444 Forest Park Blvd., Saint Louis, MO 63108 **Phone:** 314.286.1800 **Fax:** 314.286.1810 **Web:** genome.wustl.edu

Click here to access/download;Personal Cover;Reviewer Responses for Gigascience V4.docx

GIGA-D-19-00411

Point-by Point Response to reviewers' comments

Blue text: author's response.
Green text: Edited text on the manuscript.

Reviewer 1

The manuscript entitled "Comparative genomics and transcriptomics of four Paragonimus species provide insights into lung fluke parasitism and pathogenesis" provides four new draft genomes for the genus, which are a great contribution for the field. Overall, the group did a great job in the whole paper, using mostly good method strategies and were very descriptive. Here are some comments:

1-1. The data description section seems to be a poor "method" section which seems redundant. The software names are missing (just their citations are shown) and is basically the same information provided in the methods section, which is well written. If any information on this section is important to be kept, I would fit this information in the methods section and delete the whole section.

Author response: As suggested, we have removed the "data description" section and moved some of relevant details into the "methods" section.

1-2. The Analysis section should be renamed to Results and Discussion

Author response: This change has now been made.

1-3. Line 185-186 - the authors mentioned the draft genome sizes obtained and their respective completeness. Is this based in which expected complete genome size? Is there any complete genome of the genus complete (no gaps, physical evidence, telomere to telomere)? I understand that this is an estimation, but the authors should be careful and at least mention the expected "complete" genome size. Since we are talking about different species, these sizes should vary for each species.

Author response: "Completeness" is based on BUSCO completeness scores, which quantify the number of conserved genes identified among the genomes. We have changed this sentence to clarify in the results and discussion, and we have updated Table 1 to Include additional BUSCO statistics (see also response 2-3 to reviewer 2):

Section: Results and Discussion
"These draft genomes are estimated to be between 87% and 96% complete, according to BUSCO completeness estimates that include complete and fragmented eukaryote genes [17], with the new P. westermani genome produced from a sample collected from Japan being slightly more complete than the previously-sequenced genome produced from a sample collected from India [12] (96.4% vs 94.1%, respectively; Table 1)."

1-4. Line 205 - the group mentioned that some Orthologs vary in intron lengths and number of exons. Genomes that are highly repetitive (>50% repetitive) are usually very fragmented or have their most complex regions poorly assembled by short reads. Besides the group method using two libraries sizes for the Illumina applied using AllPaths, which I consider one of the best approaches for Illumina only assembly for this kind of complex genomes, there is a chance that these variations are due to problems in the assembly or frameshifts. Please provide how all these variations were validates to be real (Alignment support, etc).

Author response: In order to minimize the effect of gene fragmentation on calculating the gene/exon/intron length statistics, as stated already in the methods, these statistics "were defined using only the longest and only the complete mRNA (with identified start and stop codon) for each gene". This ensured that the genes used for the analysis were all complete and would exclude those split by repeats or short contigs. However, we

do still recognize the potential for some of the species-to-species differences to arise due to different assembly read lengths and qualities. We have appended this text acknowledging this potential issue:

Section: Results and Discussion, genome features

"Focusing on the gene content, P. kellicotti had the shortest average total gene length among the species, and the lung flukes overall had similar gene lengths to other flukes, while platyhelminth species other than trematodes have shorter genes overall (Figure 2A). The variability in gene lengths observed between species results from differences in both average intron lengths (Figure 2B) and the average number of exons per gene (Figure 2C) while the average coding sequence (CDS) lengths of the exons across all the platyhelminth species were similar to each other (Figure 2D). Whereas there was species-to-species variability in gene lengths and exon counts, consistent patterns among the types of flukes were not apparent. **Some of this variability may have arisen due to the variation in quality of the assemblies, but these differences were minimized by only using complete gene models with a start and stop codon identified in the same frame.**

1-5. Line 422 - There is no need to mention the method in the Result and Discussion section. This was also observed in other lines in the Results section (eg. Line 291, 355,etc).

Author response: We have removed references to the tools used at these lines in the text, since they are indeed mentioned already in the methods section (Interpro, Orthofinder and DESeq2, respectively).

1-6. Line 231-2 - How the identity was calculated? WGS or Orthologs? Amino-acid or Nucleotide level? This is really important when comparing identities between species, since assembly bias could be detected. This information should be added in the methods.

Author response: We reported the sequence identity between the geographically diverse *P. westermani* samples at the nucleotide level. We have revised the text to clarify this point, and expanded the methods section to describe the approach that was used to estimate the genome-wide mean divergence rate.

Section: Results and Discussion

"Although our P. westermani reference genome was assembled using samples collected from Japan (Amakusa, Kyusyu). We compared the genomic sequences of our East Asian P. westermani to the recently published P. westermani genome from India (Changlang, Arunachal Pradesh) [12] to estimate the genetic divergence between geographically diverse samples. This analysis identified an average nucleotide sequence identity of 87.6%."

Section: Methods

"MUMmer v4.0 [95] was used to estimate the level of genetic divergence between P. westermani samples from Japan and India. Nucmerum was run first to generate genome alignments using draft assembly sequences. Dnadiff was then used to calculate the average sequence identity between the genomes considering only 1-to-1 alignments."

1-7. Line 383-6 - I understand the idea of the group to give the organism-specific conserved orthologs for potential drug targeting, but when doing this I would recommend adding more information about these proteins, like localization, protein weight, TM and signal peptides, is that any hit in CHEMBL, etc. This would save time for the community that will read the paper to remove possible noise before starting to test the screening.

Author response: We have now added additional annotation data to Table S2, including (i) localization predictions by PANNZER gene ontology (cellular component category), (ii) TM and signal peptide predicitons by Phobius, (iii) protein weight predictions and pi values (ExPASy) and (iv) hits to CHEMBL. This is in addition to the existing annotations that included Interpro domains, GO categorizations, MEROPS protease predictions, and KEGG enzyme predictions. We have added the following to the text:

Section: Results and Discussion

"The comparative analysis presented here identifies valuable putative protein targets for drug development, including Paragonimus-specific proteins and trematode-conserved proteins which do not share orthology to human proteins. The protein annotation data available in Supplementary Table S2 also will enable prioritization including biological functional annotations [58, 59], protein weight and pi predictions [60], predictions of signal peptides and transmembrane domains [61] and cellular compartment localization [58], and sequence similarity matches to targets in the CHEMBL database [62]."

Section: Methods

"The completeness of annotated gene sets was assessed using BUSCO v3.0, eukaryota_odb9 [17]. Gene Ontology (GO), KEGG and protease annotations were performed using InterProScan v5.19 [59], GhostKOALA [60], and MEROPS [89], respectively. ExPASy was used to perform protein weight and pi predictions [61], SignalP was used to predict predictions signal peptides and transmembrane domains [62], and gene product localization was predicted using the "cellular component" Gene Ontology annotations provided by InterProScan [59]."

1-8. Line 419 - Discussion should change to Conclusion

Author response: This has been changed.

1-9. Line 435-443 - Fresh Paragonimus (never frozen or stored for a long time) should be better for long sequencing, since there is less chance to have their DNA broken. This could affect differences in contiguity of some genomes.

Author response: Most of the samples that were used for PacBio sequencing were prepared fresh, but if storage was required they were flash frozen at -80°C to minimize degradation.

1-10. Line 446-454 - Why the group didn't try a hybrid approach for the assembly using long and short reads together? Why PBJELLY (usually reported to be used as gap filler tool for pacbio) was used for assembly of the long reads (CANU, HGAP and FALCON are much better), maybe it should be revised.

Author response: We generated PacBio reads primarily for the purpose of assembly improvement, such as gap-closing and scaffolding. The depth of coverage was adequate (43x) to perform PBJelly-based genome improvements, but too shallow to try other approaches. At the time, long-read sequencing was still too costly to perform de novo assembly of ~1GB genome (using CANU, HGAP or FALCON).

1-11. Besides Pilon being a good choice for basecall polishing, I would recommend ICORN for the mitochondrial polishing. From my personal experience it usually corrects more regions than Pilon.

Author response: We have manually confirmed the validity of the corrections made by Pilon by critically assessing any inconsistencies between the assembly and the evidence in the reads. We have had a good experience using Pilon, and it can outperform iCORN for certain datasets (Walker et al., 2014, PMID:25409509).

1-12. There is no coverage obtained in the text about the sequencing datasets (Illumina/pacbio #X coverage). This is important to check how good was the basecall and polishing.

Author response: The coverage statistics are now included in the supplementary table:

Supplementary Table S1: *Paragonimus* genome and RNA-Seq accessions

Genome assemblies, annotations and raw reads

Species	NCBI accession	Bioproject ID	Genome coverage (x)
Paragonimus miyazakii	JTDE00000000	PRJNA245325	162
Paragonimus heterotremus	LUCH00000000	PRJNA284523	81
Paragonimus kellicotti	LOND00000000	PRJNA179523	77 (43*)
Paragonimus westermani	JTDF00000000	PRJNA219632	152

*Pacbio dataset coverage

1-13. And here are some minor points: Line 113 - P. westermani is not in italic;

Author response: This has been fixed.

1-14. Line 513 - change 3 for "three";

Author response: This has been fixed.

1-15. Figures are in low resolution.

Author response: The PDF displays the figures in low resolution, but they are all the maximum width and resolution specified (6.693 inches wide, 300 dpi) in their uploaded format, which can be accessed by clicking the top-right of each page.

Reviewer 2

The submitted manuscript describes the sequencing, assembly, annotated and analysis of four species of the genus Paragonimus. The sequencing was predominantly Illumina short reads, with PacBio long reads generated for P. kellicotti. The authors conduct different gene family analyses, propose molecular components of host-parasite interactions, and identify proteins which are potential targets for vaccines or diagnostics. The authors also generate some RNA-Seq data for each species.

The generation and presentation of genomic assemblies for these four species will be useful in understanding their biology and developing new treatment. For the most part the manuscript is well written and easy to understand, for which the authors should be commended. However, I do have major concerns with the manuscript as presented.

2-1: I tried to download much of the data to repeat the analyses but the speed of connection was slow. Therefore, I have looked into one section in more detail, the prediction of mimicry between Paragonimus proteins and their hosts. From lines 330 to 347, the authors describe orthologous genes (OGs) which are shared between at least one species of Paragonimus and their host to the exclusion of other trematodes (Figure 5D). The authors then speculate that these "may have evolved uniquely in lung flukes to mimic host factors[.]" Unfortunately, this is an artefact of sampling bias. I used BLAST to compare human STOX1, Zip67, and C5orf63 with Panagonimus, Schmidtea mediterranea and Caenorhabditis elegans proteins. For the first two, it is clear sequence similarity is similar or greater in S. mediterranea and C. elegans, raising reasonable doubt on specific mimicry between Paragonimus and human proteins. For C5orf63, the evalue of the alignment with a P. westermani protein was 0.041 and over only 40 amino acids. This suggests that it is an artifact of the clustering process in the OG generation.

```
blastp -outfmt 6 -max_hsps 1 -query STOX1.pep.fsa -db ../data/all.protein.fa | head -5
STOX1_HUMAN    F53B2.6 33.758  157     102     1       33      189     16      170     3.44e-28        120
STOX1_HUMAN    SMEST040264001  29.348  184     130     0       19      202     15      198     2.75e-22        103
STOX1_HUMAN    PKEL_11588      35.088  114     71      2       33      144     28      140     2.39e-13        71.6
STOX1_HUMAN    PMIY_01855      33.043  115     74      2       32      144     27      140     3.42e-12        72.0
STOX1_HUMAN    PWES_01040      33.628  113     72      2       34      144     29      140     1.20e-09        63.2

blastp -outfmt 6 -max_hsps 1 -query Zip67.pep.fsa -db ../data/all.protein.fa | head -6
ZN653_HUMAN    F45B8.4 33.918  171     106     4       442     612     101     264     7.44e-22        98.6
```

ZN653_HUMAN	SMEST004840001	44.048	84	47	0	496	579	211	294	1.50e-18	89.0
ZN653_HUMAN	SMEST060422001	36.607	112	68	2	469	577	464	575	2.11e-18	91.7
ZN653_HUMAN	SMEST058261001	35.484	155	92	5	460	614	77	223	1.63e-17	86.7
ZN653_HUMAN	SMEST042630001	36.885	122	73	2	490	611	183	300	6.75e-17	84.3
ZN653_HUMAN	PMIY_03311	46.988	83	44	0	496	578	200	282	7.00e-17	83.6

```
Query= YD286_HUMAN Glutaredoxin-like protein C5orf63 OS=Homo sapiens
OX=9606 GN=C5orf63 PE=2 SV=3
Length=138
                                                              Score        E
Sequences producing significant alignments:                  (Bits)     Value
PWES_06707                                                    33.5       0.041
>PWES_06707
Length=136
 Score = 33.5 bits (75),  Expect = 0.041, Method: Compositional matrix adjust.
 Identities = 17/43 (40%), Positives = 23/43 (53%), Gaps = 2/43 (5%)
Query  16  FGLFLRNCSASKTTLPVLTLFTKDPCPLCDEAKEVLKPYENRQ  58
           G ++   S +K  LP L +FTK  C LC  A   L+PY N+
Sbjct  26  LGQYISTISIAK--LPTLIVFTKPDCSLCKAAIVQLQPYVNKH  66
```

I recommend that the authors rethink their strategy for identifying molecular mimicry or remove the section entirely.

Author response: We acknowledge that OrthoFinder's classification system may produce false positives in terms of species-restricted orthology, since in some cases such as those described here, similar genes may be divided into several orthogroups. In order to avoid overstating the significance of these particular orthologous groups, we have taken the reviewer's suggestion and removed this section from the manuscript, along with the accompanying Figure 5D and Table 3, and other mentions throughout the text.

2-2. The authors generated several RNA-Seq datasets for each species. Most of these were done single copies. Where replication was done, the authors note that it they are 'technical replicates', from which I understand that the samples are from the same biological source but run sequenced twice. These data are great for genome annotation, i.e. the identification of gene models. But, the accurate identification differentially expressed genes requires biological replicates. The authors' use of DESeq is not appropriate given the available data. Further, they should not be comparing FPKM as a statistically robust method to determine differential gene expression. Traditionally, people have asked for three biological replicates, though in depth modelling has shown that one needs to consider sequencing depth in addition to replication. I encourage the authors to read Schurch et al. https://www.ncbi.nlm.nih.gov/pubmed/27022035. I do appreciate that getting sufficient number of biological replicates in parasite systems is a challenge. However, this cannot justify having insufficient power in an analysis. Better not to conduct the analysis at all. I recommend that all references to differentially expressed genes is removed from the manuscript.

Author response: The "technical replicates" are only indicated in order to clarify that there are two accessions on NCBI for the same samples. For all downstream analysis, mapped read counts were summed for technical replicates to generate a single biological replicate. We recognize that these should never be used for statistical purposes. As the reviewer acknowledges, it is difficult to obtain biological samples. Given the limitations of the dataset, we have made the best possible use of the data and treated the three samples from the "cavities" as "replicates" (one pleural cavity, two biological replicates from the peritoneal cavity), and the two samples from the "tissues" as "replicates" (one from the lung, one from the liver). See the bottom-right corner of Figure 6C for how those samples cluster and how they were used. Using these different sample types as replicates will increase noise since they will vary within groups, which is why we see a relatively low number of differentially expressed genes (216 higher in cavities, 172 higher in tissues), but we feel that as long as it is not misrepresented in the text as direct biological replicates of the exact same tissues, there is nothing statistically incorrect about performing the analysis this way, especially when the alternative is having very little analysis of these rare and valuable samples.

FPKM values were only calculated for providing normalized expression values (normalized to library size and gene length), for the purposes of visualization (Figure 6) and data presentation (Table 4). DESeq2 was ran using raw read counts per gene per sample, as indicated in its manual, since it uses a negative binomial

statistic. Supplementary Table S2 also provides both the raw read counts and the normalized expression values, so that readers can run additional analyses using either value. The relevant DESeq output (FDR-adjusted P values and log2 fold changes) are also provided in that table, for every gene, so that the analysis process is as transparent as possible.

We recognize that more biological replicates from each tissue would be ideal, but since these are very difficult to obtain and since this analysis is not the main focus of the manuscript (just two paragraphs in results/discussion), we feel that it is still valuable to provide a preliminary analysis of the transcriptional differences during parasitism in Paragonimus.

To more clearly demonstrate the correlation values between the sample sets, we have now added **Supplementary Figure S4,** which shows the complete correlation table in addition to the clustering:

Section: Supplementary Information

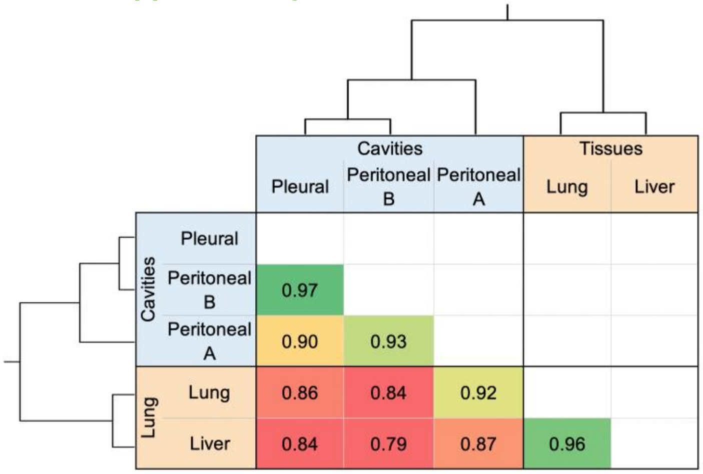

Supplementary Figure S4: Pearson correlation values between *Paragonimus miyazakii* RNA-seq samples, based on overall gene expression patterns.

We also now reference this more directly in the text:

Section: Results and Discussion - Gene expression analysis identifies stage-specific lung fluke functions

"Worms from additional life cycle stages were collected for *P. miyazakii*, including samples sequenced from cavities (peritoneal and pleural cavities) and tissues (lung and liver). Based on gene expression profiles across all genes, the cavity samples clustered and correlated more closely with each other than with the peritoneal samples (and vice versa; Supplementary Figure S1)."

Unlike with the differential expression data, the expression data for conserved-and-exclusive orthologous groups did use single replicate data. We have now added the following to directly acknowledge the lack of replication used for this approach:

Section: Results and Discussion - Gene expression analysis identifies stage-specific lung fluke functions

"However, to confirm these gene expression patterns for specific larval stages, followup studies with additional biological replicates are needed."

2-3: The reported BUSCO scores are between 86% and 96% (Table 1). When comparing to parasite.wormbase, three of these Paragonimus assemblies would have the highest BUSCO score for any platyheliminth species and all are far above the best trematode, a reference quality assembly of S. mansoni. Further, in Table 1, the authors report a BUSCO score of 94.1% for P. westermani (India) previously sequenced (Oey et al.). However, Oey reports a BUSCO score of 65.3%. I ran BUSCO on P. westermani

(Japan) using the eukayota orthologue set (-l eukaryota_odb9) and got "C:77.9%[S:76.9%,D:1.0%],F:8.9%,M:13.2%,n:303". I presume that the authors used a different orthologue set for the "--lineage", but they do not state which one. Please can the authors provide further clarification.

Author response: We ran BUSCO v 3.0 using the eukaryota_odb9. This was ran against the gene set to better reflect the quality of both the assembly and the annotation. However, in our estimates of completeness, we had included the “fragmented” genes as being present. Since these may or may not be considered “complete”, we have now expanded Table 1 to include the classifications of BUSCO genes (complete single copy, complete duplicated, fragmented, missing, and overall completeness that includes the complete + fragmented, as before). Our numbers for P. westermani (Japan) are slightly higher than the numbers provided by the reviewer here because our assembly has been updated (88.78% complete, singly copy, vs 77.9%). Both Oey and WormBase Parasite use the metazoan lineage dataset (n = 978) rather than eukaryota (n = 303), and according to Wormbase’s release notes, they are still using BUSCO 2.0 (Dec 2018 update). Using WormBase Parasite’s approach, even *Schistosoma mansoni*, which has a chromosome-scale assembly (PMID: 22253936) has only 78.9% completeness (71.2% complete single, 3.5% duplicated, 4.2% fragmented), so we feel that using eukaryote is more reflective of accurate genome completeness. This same approach has been used for other helminth species including *Heterohabditis bacteriophora* in Gigascience, 2019 (PMID: 29617768).

In addition to updating Table 1 (below), we have added additional clarification in the text:

Section: Results and Discussion
“These draft genomes are estimated to be between 87% and 96% complete (**according to BUSCO completeness estimates that include complete and fragmented eukaryote genes [17]**), with the new P. westermani genome produced from a sample collected from Japan being slightly more complete than the previously-sequenced genome produced from a sample collected from India [12] (96.4% vs 94.1%, respectively; Table 1).”

Section: Methods
“The completeness of annotated gene sets was assessed using BUSCO v3.0, **eukaryota_odb9** [17].”

Table 1: The draft genome of *Paragonimus*: assembly, size and annotation characteristics

Statistic	*Paragonimus miyazakii*	*Paragonimus heterotremus*	*Paragonimus kellicotti*	*Paragonimus westermani* (Japan)	*Paragonimus westermani* (India)
**Assembly statistics**					
Total genome length (Mb)	915.8	841.2	696.5	923.3	922.8
Number of contigs	22,318	27,557	29,377	22,477	30,455
Mean contig size (kb)	41	30.5	23.7	41.1	30.3
Median contig size (kb)	15.1	9.3	10.2	17.2	4.8
Max. contig size (kb)	919.8	715.6	826	829	809.4
N50 length (kb)	108.8	92.5	56.0	100.8	135.2
N50 number	2,320	2,506	3,316	2,664	1,943
**BUSCO completeness (303 genes, eukarota_odb9)**					
Complete, single copy	84.5%	82.5%	70.3%	88.78%	76.90%
Complete, duplicated	1.3%	0.0%	1.3%	1.32%	2.31%
Fragmented	7.6%	10.9%	15.2%	6.27%	14.85%
Missing	6.6%	6.6%	13.2%	3.63%	5.94%
Overall completeness	93.4%	93.4%	86.8%	96.37%	94.06%
**Gene statistics**					
Number of genes	12,652	12,490	12,853	12,072	12,771

Avg gene length (kb)	25.9	22.6	17.6	24.1	18.0
Avg CDS length (kb)	1.5	1.4	1.1	1.4	1.4
Avg intron length (kb)	4.2	4	3.6	4.2	4.0
Avg # exons per gene	6.7	6.2	5.3	6.3	5.2
% annotated InterPro	82%	85%	81%	87%	82%
% annotated KEGG	40%	41%	34%	43%	43%

2-4. On reviewing the methods, I could not find sufficient detail to rerun many of the analyses properly. I recommend that the authors provide a file with all the commands, options and software versions. This file serves two purposes. The first is so that replication of the work will support its robustness. The second is so that other researchers can implement these methods for their own species of interest.

Author response: Following the reviewer's recommendation, we have provided the complete commands and parameters used to perform the analyses as Supplementary Text S1.

We have also added the following text to indicate that this information is available:

Sections: Methods, and '**Availability of supporting data and materials**'
"All relevant software versions, and commands specifying the parameters used are presented in **Supplementary Text S1**."
