# Supplementary material for: Comparative genomics and transcriptomics of 4 Paragonimus species provide insights into lung fluke parasitism and pathogenesis
Source: Gigascience. 2020 Jul 20;9(7):giaa073. doi: 10.1093/gigascience/giaa073 (PMC7370270; doi:10.1093/gigascience/giaa073)
Supplement: giaa073_GIGA-D-19-00411_Revision_2 [file giaa073_giga-d-19-00411_revision_2.pdf]

GigaScience

Comparative genomics and transcriptomics of four Paragonimus species provide insights into lung fluke parasitism and pathogenesis

--Manuscript Draft--

**Manuscript Number:**	GIGA-D-19-00411R2
**Full Title:**	Comparative genomics and transcriptomics of four Paragonimus species provide insights into lung fluke parasitism and pathogenesis
**Article Type:**	Research
**Funding Information:**	National Institutes of Health - National Human Genome Research Institute (U54HG003079) — Dr. Makedonka Mitreva<br>National Institutes of Health - National Institute of Allergy and Infectious Diseases (AI081803) — Dr. Makedonka Mitreva<br>National Institutes of Health - National Institute of General Medical Sciences (GM097435) — Dr. Makedonka Mitreva<br>Thailand Research Fund (TH) - Distinguished Research Professor Grant (DPG6280002) — Dr. Wanchai Maleewong

Abstract:

Background

Paragonimus spp . (lung flukes) are among the most injurious food-borne helminths, infecting ~23 million people, (~293 million with infection risk). Paragonimiasis is acquired from infected undercooked crustaceans and primarily affects the lungs, but often causes lesions elsewhere including the brain. The disease is easily mistaken for tuberculosis due to similar pulmonary symptoms, and accordingly, diagnostics are in demand.

Results

We assembled, annotated and compared draft genomes of four prevalent and distinct Paragonimus species: P. miyazakii , P. westermani , P. kellicotti and P. heterotremus . Genomes ranged from 697 to 923 Mb, included 12,072 to 12,853 genes, and were 71.6% to 90.1% complete according to BUSCO. Orthologous group (OG) analysis spanning 21 species (lung, liver and blood flukes, additional platyhelminths and hosts) provided insights into lung fluke biology, including identifying 256 lung fluke-specific and conserved OGs enriched for iron acquisition, immune modulation and other parasite functions. Transcriptome analysis identified consistent adult-stage Paragonimus expression profiles, and previously identified Paragonimus diagnostic antigens were matched to genes, providing an opportunity to optimize and ensure pan- Paragonimus -reactivity for diagnostic assays.

Conclusions

This report provides advances in molecular understanding of Paragonimus and underpins future studies into the biology, evolution and pathogenesis of Paragonimus and related food-borne flukes. We anticipate that these novel genomic and transcriptomic resources will be invaluable for future lung fluke research.

**Corresponding Author:**	Makedonka Mitreva<br><br>UNITED STATES
**Corresponding Author Secondary Information:**	
**Corresponding Author's Institution:**	
**Corresponding Author's Secondary Institution:**	
**First Author:**	Bruce A Rosa
**First Author Secondary Information:**	
**Order of Authors:**	Bruce A Rosa
	Young-Jun Choi
	Samantha N McNulty
	Hyeim Jung
	John Martin
	Takeshi Agatsuma
	Hiromu Sugiyama
	Thanh Le Hoa
	Pham Ngoc Doanh
	Wanchai Maleewong
	David Blair
	Paul J. Brindley
	Peter U. Fischer
	Makedonka Mitreva
**Order of Authors Secondary Information:**	
**Response to Reviewers:**	GIGA-D-19-00411R1<br><br>Point-by Point Response to editor and reviewers' comments<br><br>Editor:<br><br>We appreciate your email and your reasons for keeping this as a Research paper; however, we strongly suggest that this falls within our Data Note criteria - as the analysis you present is the validation that we ask for in a Data Note. So with this round of review, I'd like you to consider Reviewer #2's comments, as well as ours, and decide whether to change this into a Data Note or keep it as Research - removing the RNA-seq data for the specie, P. miyazakii.<br><br>Response:<br><br>We have completely removed the cavity vs tissue RNAseq analysis for the species, P. miyazakii. RNAseq analyses of the other species are presented in relevant sections. The key analyses include: i) validation of gene expression of all lung fluke-specific and conserved orthologous protein families (highly relevant analysis related to evolutionary adaptations in the genus Paragonimus), and ii) characterization of orthology among genes across this genus. The outcome confirmed consistent levels of expression of adult-stage genes supported by high Pearson correlations values.<br><br>Editor:<br><br>I would also like to point out that our Data Notes are indexed the same way as

Powered by Editorial Manager® and ProduXion Manager® from Aries Systems Corporation

Research papers, and they are also half the cost of publishing a Research paper. If you choose to keep your paper as Research, we will however have to send it for a third round of review with an Editorial Board member.

Response:

With regard to the suggestion to reassign the manuscript to Data Note, we have re-reviewed the scope of both manuscript types. The ‘Research Article’ type is for “Manuscripts containing more detailed biological, medical or technical analyses of data” whereas Data Notes “focus on a particular dataset, and provide detailed methodology on data production, validation, and potential reuse.” The rationale and findings of our manuscript are clearly within the ambit of the journal’s Research Article, and indeed better fit there than in the Data Note section. By contrast, the Data Note type is intended “to incentivize and more rapidly release data before subsequent detailed analysis has been carried out.” Specifically, this is because we first strategically choose four species spanning the genus Paragonimus to facilitate the presented analysis. Hence, in addition of presenting four novel genomes of neglected tropical disease pathogens, and transcriptomes for three of the species, we undertook detailed technical comparative analyses for four species of Paragonimus (lung flukes) and 17 other phylogenetically relevant species. The endeavor revealed key evolutionary genetic changes underlying diversification with the genus Paragonimus (e.g., gene family evolution and positive selection), which has provided novel biological knowledge about the tissue tropism of these medically important pathogens, which are among the most injurious food-borne helminths, infecting ~23 million people, with ~293 million people at risk for infection. Furthermore, the disease is frequently misdiagnosed as tuberculosis due to similar pulmonary symptoms (and maybe even for COVID-19), so there is an urgent need for the development of effective diagnostics. Because we strategically selected to sequence and analyze these 4 species that span the genus Paragonimus, we were able to match the new gene sets to previously identified single species-based Paragonimus diagnostic antigens, providing an opportunity to optimize and ensure consistent cross-reactivity for diagnostic assays, which is of a direct clinical. Thus, we have performed a thorough and extensive analysis of the available datasets for Paragonimus to provide insights of biological and clinical relevance. The manuscript provides far, far more than (to paraphrase the Data Note type) ‘a rapid release of a novel dataset that we wish to incentivize’.

Reviewer 1:

The group answered all questions posted before and made the changes properly to make the manuscript more clear. Just two minor points:

1.1. Line 429 - maybe a typo. I believe that the author wanted to mention "Ncmer" instead of "Nucmerum";

Response: This has been fixed.

1.2. Line 394 - The authors reported well why they use PBJelly, but my point is that PBJELLY is not an assembler. " For P. kellicotti, PacBio were assembled using PBJelly". PBJelly, is a polishing tool to upgrade draft assemblies. Using it was correct, but the word assembler should be removed.

Response: This has been fixed.

Reviewer 2:

2.1. I thank the authors for the detailed response to my concerns. Regarding the RNA-Seq data, I appreciate that these samples are precious. However, that does not compensate for the lack biological replicates. If insufficient material cannot be obtained than the experiment should be considered. I am unable to support the strategy to consider the pleural and two peritoneal samples as replicates for the "cavities", nor lung and liver to be considered replicates for "tissues". Figure S4 doesn't provide strong support for this division; the pearson correlation values are nearly identical for peritoneal B vs peritoneal A (0.93), as peritoneal A vs lung (0.92). Even if one were to

	ignore this, there remains the problem that "tissues" has only two replicates. Further, single samples are used to make claims as to expression, for example in line 302 and table 4. My resolute stance on the shortcomings of this analysis, is because the work presented will be cited by others with confidence and may lead to a snow-balling of over-interpretation that can have significant and negative impact on research into these important parasites. Due to the problems I list, I recommend that the expression section is removed from the analysis.<br><br>Response: The RNAseq analysis of the cavities vs tissue of P. miyazakii has been removed.<br><br>2.2. Regarding the measures of completeness, I thank the authors for providing more details. I agree with their use of the eukaryotic set of conserved genes in BUSCO. I disagree with the claim that "fragmented" genes "may or may not be considered complete." I challenge the authors to provide published examples of this. The paper on the Heterohabditis genome does also use the eukaryote set, but does not claim fragmented genes are complete. The authors, in their response, offer S. mansoni's completeness of 73.8% as a comparison. In Wormbase-Parasite, all of the Schistosoma species have relative low scores on both CEGMA and BUSCO. It has been hypothesised that the blood flukes have lost a suite of genes previously thought to be highly conserved. Perhaps more impressive for the presented Paragonimus assemblies is the low proportion of duplicated complete genes. This suggests a low level of mis-assembly due to heterozygosity. I strongly encourage the authors to remove the fragmented genes from this "overall completeness" score in Table 1 and throughout the text.<br><br>Response: We have modified the R2 text along the lines suggested by the reviewer. We also removed the “overall completeness” category from the Table 1.
**Additional Information:**	
**Question**	**Response**
Are you submitting this manuscript to a special series or article collection?	No
**Experimental design and statistics**<br><br>Full details of the experimental design and statistical methods used should be given in the Methods section, as detailed in our Minimum Standards Reporting Checklist. Information essential to interpreting the data presented should be made available in the figure legends.<br><br>Have you included all the information requested in your manuscript?	Yes
**Resources**<br><br>A description of all resources used, including antibodies, cell lines, animals and software tools, with enough information to allow them to be uniquely identified, should be included in the	Yes

Methods section. Authors are strongly encouraged to cite Research Resource Identifiers (RRIDs) for antibodies, model organisms and tools, where possible.<br><br>Have you included the information requested as detailed in our Minimum Standards Reporting Checklist?	
**Availability of data and materials**<br><br>All datasets and code on which the conclusions of the paper rely must be either included in your submission or deposited in publicly available repositories (where available and ethically appropriate), referencing such data using a unique identifier in the references and in the “Availability of Data and Materials” section of your manuscript.<br><br>Have you have met the above requirement as detailed in our Minimum Standards Reporting Checklist?	Yes

Manuscript Click here to access/download;Manuscript;Paragonimus_Genomes_REVISE

Comparative genomics and transcriptomics of four *Paragonimus* species provide insights into lung fluke parasitism and pathogenesis

Bruce A. Rosa[1]*, Young-Jun Choi[1]*, Samantha N. McNulty[2], Hyeim Jung[1], John Martin[1], Takeshi Agatsuma[3], Hiromu Sugiyama[4], Thanh Le Hoa[5], Pham Ngoc Doanh[6,7], Wanchai Maleewong[8], David Blair[9], Paul J. Brindley[10], Peter U. Fischer[1], Makedonka Mitreva[1,2†]

[1]Department of Internal Medicine, Washington University School of Medicine, St. Louis, MO 63110, USA

[2]The McDonnell Genome Institute at Washington University, School of Medicine, St. Louis, MO 63108, USA

[3]Department of Environmental Health Sciences, Kochi Medical School, Oko, Nankoku City, Kochi 783-8505, Japan

[4]Laboratory of Helminthology, Department of Parasitology, National Institute of Infectious Diseases, Tokyo 162-8640, Japan

[5]Department of Immunology, Institute of Biotechnology, Vietnam Academy of Science and Technology, Hanoi, Vietnam

[6]Institute of Ecology and Biological Resources, Vietnam Academy of Science and Technology, Hanoi, Vietnam

[7]Graduate University of Science and Technology, Vietnam Academy of Science and Technology, Hanoi, Vietnam

[8]Research and Diagnostic Center for Emerging Infectious Diseases, Khon Kaen University, Khon Kaen, Thailand, Department of Parasitology, Faculty of Medicine, Khon Kaen University, Khon Kaen, Thailand

[9]College of Marine and Environmental Sciences, James Cook University, Townsville, Queensland 4811, Australia

[10]Departments of Microbiology, Immunology and Tropical Medicine, and Research Center for Neglected Diseases of Poverty, and Pathology School of Medicine & Health Sciences, George Washington University, Washington, DC 20037, USA

*Authors contributed equally to this work

†Correspondence should be addressed to Makedonka Mitreva. Tel. +1-314-285-2005, Fax +1-314-286-1800, Email: mmitreva@wustl.edu

Emails:
Bruce A. Rosa: barosa@wustl.edu

Young-Jun Choi: choi.y@wustl.edu

Samantha N. McNulty: samantha.n.mcnulty@gmail.com

Hyeim Jung: jungh@wustl.edu

John Martin: jmartin@wustl.edu

Takeshi Agatsuma: agatsuma@kochi-u.ac.jp

Hiromu Sugiyama: hsugi@niid.go.jp

Thanh Le Hoa: imibtvn@gmail.com

Pham Ngoc Doanh: pndoanh@yahoo.com

Wanchai Maleewong: wanch_ma@kku.ac.th

David Blair: david.blair@jcu.edu.au

Paul J. Brindley: pbrindley@gwu.edu

Peter U. Fischer: pufischer@wustl.edu

Makedonka Mitreva: mmitreva@wustl.edu

Keywords

Lung flukes, genomics, transcriptomics, paragonimiasis, infectious disease, trematodes

Abstract

Background

Paragonimus spp. (lung flukes) are among the most injurious food-borne helminths, infecting ~23 million people, (~293 million with infection risk). Paragonimiasis is acquired from infected undercooked crustaceans and primarily affects the lungs, but often causes lesions elsewhere including the brain. The disease is easily mistaken for tuberculosis due to similar pulmonary symptoms, and accordingly, diagnostics are in demand.

Results

We assembled, annotated and compared draft genomes of four prevalent and distinct *Paragonimus* species: *P. miyazakii*, *P. westermani*, *P. kellicotti* and *P. heterotremus*. Genomes ranged from 697 to 923 Mb, included 12,072 to 12,853 genes, and were 71.6% to 90.1% complete according to BUSCO. Orthologous group (OG) analysis spanning 21 species (lung, liver and blood flukes, additional platyhelminths and hosts) provided insights into lung fluke biology, including identifying 256 lung fluke-specific and conserved OGs enriched for iron acquisition, immune modulation and other parasite functions. Transcriptome analysis identified consistent adult-stage *Paragonimus* expression profiles, and previously identified *Paragonimus* diagnostic antigens were matched to genes, providing an opportunity to optimize and ensure pan-*Paragonimus*-reactivity for diagnostic assays.

Conclusions

This report provides advances in molecular understanding of *Paragonimus* and underpins future studies into the biology, evolution and pathogenesis of *Paragonimus* and related food-borne flukes. We anticipate that these novel genomic and transcriptomic resources will be invaluable for future lung fluke research.

Background

The trematode genus *Paragonimus*, the lung flukes, is among the most injurious taxon of food-borne helminths. About 23 million people are infected with lung flukes [1], an estimated 292 million people are at-risk, mainly in eastern Asia [2] , and billions of people live in areas where *Paragonimus* infections of animals are endemic. The life-cycle of *Paragonimus* species involves freshwater snails, crustacean intermediate hosts and mammals in Asia, parts of Africa, and the Americas [3]. Human paragonimiasis is acquired by consuming raw or undercooked shrimp and crabs containing the metacercaria, which is the infective stage. Although primarily affecting the lungs, lesions can occur at other sites, including the brain [4], and pulmonary paragonimiasis is frequently mistaken for tuberculosis due to similar respiratory symptoms [4].

Pathogenesis ensues because of the migration of the newly invading juveniles from the gut to the lungs and through not-infrequent ectopic migration to the brain, reproductive organs, and subcutaneous sites at the extremities, and because of toxins and other mediators released by the parasites during the larval migration [4, 5]. The presence of the flukes in the lung causes hemorrhage, inflammation with leukocytic infiltration and necrosis of lung parenchyma that gradually proceeds to the development of fibrotic encapsulation except for a fistula from the evolving lesion to the respiratory tract. Eggs of the lung fluke exit the encapsulated lesion through the fistula to reach the sputum and/or feces of the host, where they pass to the external environment, accomplishing transmission of the parasite [6]. There are signs and symptoms that allow characterization of acute and chronic stages of paragonimiasis. In pulmonary paragonimiasis, for example, the most noticeable clinical symptom of an infected individual is a chronic cough with gelatinous, rusty brown, pneumonia-like, blood-streaked sputum [6]. Heavy work commonly induces hemoptysis. Pneumothorax, empyema from secondary bacterial infection and pleural effusion might also be presented. When symptoms include only a chronic cough, the disease may be misinterpreted as chronic bronchitis and bronchiectasis or bronchial asthma. Pulmonary paragonimiasis is frequently confused with pulmonary tuberculosis [7]. The symptoms of extra-pulmonary paragonimiasis vary depending on the location of the fluke, including cerebral [5] and abdominal paragonimiasis [6].

Paragonimus is a large genus that includes more than 50 nominal species [8]. Seven of these species or species complexes of *Paragonimus* are known to infect humans [3]. This is also an ancient genus, thought to have originated before the breakup of Gondwana [9], but possibly also dispersing as colonists from the original East

Asian clade, based on the distribution of host species [10]. To improve our understanding of pathogens across this genus at the molecular level, we have assembled, annotated and compared draft genomes of four of these, three from Asia (*P. westermani* from Japan, *P. heterotremus, P. miyazakii*) and one from North America (*P. kellicotti*). Among them, *P. westermani* is the best-known species causing pulmonary paragonimiasis. This name has been applied to a genetically and geographically diverse complex of lung fluke populations differing widely in biological features including infectivity to humans [11]. The complex extends from India and Sri Lanka eastwards to Siberia, Korea and Japan, and southwards into Vietnam, Indonesia and the Philippines. However, human infections are reported primarily from China, Korea, Japan and the Philippines. Until this study, an Indian member of the *P. westermani* complex was the only lung fluke species for which a genome sequence was available [12]. *Paragonimus heterotremus* is the most common cause of pulmonary paragonimiasis in southern China, Lao PDR, Vietnam, northeastern India and Thailand [6, 8]. *Paragonimus miyazakii* is a member of the *P. skrjabini* complex, to which Blair and co-workers accorded sub-specific status [13]. Flukes of this complex tend not to mature in humans but frequently cause ectopic disease at diverse sites, including the brain. In North America, infection with *P. kellicotti* is primarily a disease of native, crayfish-eating mammals including the otter and mink. The occasional human infections can be severe, and thoracic involvement is typical [14, 15].

These four species represent a broad sampling of the phylogenetic diversity of the genus. Most of the known diversity, as revealed by DNA sequences from portions of the mitochondrial genome and the nuclear ribosomal genes, resides in Asia [16]. Analysis of the ITS2 marker by Blair et al [16] indicates that each of the species sequenced occupies a distinct clade within the phylogenetic tree.

In addition to a greater understanding of the genome contents of this group of food-borne trematodes, the findings presented here provide new information to assist development of diagnostic tools and recognition of potential drug targets. The findings will facilitate evolutionary, zoogeographical and phylogenetic investigation of the genus *Paragonimus* and its host-parasite relationships through the comparative analysis of gene content relative to other sequenced platyhelminth and host species, and to known *Paragonimus* diagnostic antigen targets.

Results and Discussion

Genome features

The sizes of the four novel *Paragonimus* genomes range from 697 to 923 Mb, containing between 12,072 and 12,853 genes. These draft genomes are estimated to be between 71.6% and 90.1% complete, according to the number of complete BUSCO eukaryote genes (single-copy or duplicate) [17], with the new *P. westermani* genome produced from a sample collected from Japan being more complete than the previously-sequenced genome produced from a sample collected from India [12] (90.1% vs 70.2%, respectively; **Table 1**). Here, statements about *P. westermani* apply to the new Japanese genome, unless otherwise stated. The total genome lengths of the *Paragonimus* spp. are larger than those of the Schistosomatidae and Opisthorchiidae, but smaller than those of Fasciolidae. However, the total numbers of protein-coding genes are comparable (**Table 1**). Repetitive sequences occupy between 49% and 54% of the *Paragonimus* genomes (**Figure 1A**). The repeat landscapes, depicting the relative abundance of repeat classes in the genome, versus the Kimura divergence from the consensus, revealed that *P. kellicotti* in particular has a significant number of copies of transposable elements (TE) with high similarity to consensus (Kimura substitution level: 0-5), indicating recent and current TE activity (**Figure 1B**). In a recent study [18], TE activity in the Fasciolidae was found to be low. TEs are potent sources of mutation that can rapidly create genetic variance, especially following genetic bottlenecks and environmental changes, providing bursts of allelic and phenotypic diversity upon which selection can act [19, 20]. Therefore, changes in TE activity, modulated by environmentally induced physiological or genomic stress, may have a major effect on adaptation of populations and species facing novel habitats and large environmental perturbations [21].

Focusing on the gene content, *P. kellicotti* had the shortest average total gene length among the species, and the lung flukes overall had similar gene lengths to other flukes, while platyhelminth species other than trematodes have shorter genes overall (**Figure 2A**). The variability in gene lengths observed between species results from differences in both average intron lengths (**Figure 2B**) and the average number of exons per gene (**Figure 2C**) while the average coding sequence (CDS) lengths of the exons across all the platyhelminth species were similar to each other (**Figure 2D**). Whereas there was species-to-species variability in gene lengths and exon counts, consistent patterns among the types of flukes were not apparent. Some of this variability may have

arisen due to the variation in quality of the assemblies, but these differences were minimized by only using complete gene models with a start and stop codon identified in the same frame.

Mitochondrial whole genome-based clustering was performed for the four *Paragonimus* species plus some additional existing mitochondrial genome assemblies for *P. ohirai* and four for *P. westermani*, including previously-sequenced mitochondrial genomes of *Paragonimus* (**Figure 3A**). This indicated that our Japanese *P. westermani* sample clustered with the existing known *P. westermani* samples from eastern Asia, and that all the other three newly sequenced species were distinct from *P. ohirai*.

We generated a PacBio long-read based mitochondrial assembly for *P. kellicotti*. The fully circularized complete genome was 17.3 kb in length, including a 3.7 kb non-coding repeat region between $tRNA^{Gly}$ and *cox3* (**Supplementary Figure S1**). There are seven copies of long repeats (378 bp) and 9.5 copies of short repeats (111 bp). The long repeats overlap with six copies of $tRNA^{Glu}$. This structural organization of repeat sequences does not resemble those found in *Paragonimus ohirai* [12] and *P. westermani* [12] where the non-coding region is partitioned by $tRNA^{Glu}$ into two parts.

Clustering of the four new lung fluke genomes, four liver fluke genomes, three blood fluke genomes, five other platyhelminth species, four host species and a yeast outgroup was performed based on the shared phylogeny among orthologous protein groups. These findings mirrored the mitochondrial clustering results for the lung fluke species (**Figure 3B**), indicating that *P. westermani* is the earlier-diverging taxon, as previously suggested based on ribosomal RNA [22].

Although our *P. westermani* reference genome was assembled using samples collected from Japan (Amakusa, Kyusyu). We compared the genomic sequences of our East Asian *P. westermani* to the recently published *P. westermani* genome from India (Changlang, Arunachal Pradesh) [12] to estimate the genetic divergence between geographically diverse samples. This analysis identified an average nucleotide sequence identity of 87.6%.

<u>Gene-family dynamics identify expanded functions distinguishing lung fluke species</u>

We investigated large-scale differences in gene complements among families of digenetic trematodes (**Figure 4A**) and modeled gene gain and loss while accounting for the phylogenetic history of species [23]. Gene families of interest that displayed pronounced differential expansion or contraction (**Figure 4B**) included the

papain-family cysteine proteases, cathepsins L, B and F, dynein heavy chain, spectrin/dystrophin, heat shock 70 kDa protein, major vault protein, and multidrug resistance protein. Total protease and protease inhibitor counts are shown in **Figure 4C**. Cathepsin F genes may have roles in nutrient digestion and remodeling of other physiologically active molecules, and Ahn et al. [24] reported differential expression of cathepsin F genes during development of *P. westermani*, and showed that most are highly immunogenic. This flagged them as prospective diagnostic targets. The importance of cathepsin F for *Paragonimus* contrasts with its function in the fasciolids, where cathepsin L genes are expanded and are thought to play a more critical role in host invasion [18, 25].

Differential expansion of cytoskeletal molecules is of interest in the context of tegument physiology [26]. Dynein is a microtubule motor protein, which transports intracellular cargo. Spectrin is an actin-binding protein, with a key role in maintenance of integrity of the plasma membrane. Dystrophin links microfilaments with extracellular matrix. The syncytial tegument of the surface of flatworms is a complex structure and a major adaptation to parasitism, and plays critical roles in nutrient uptake, immune response modulation and evasion, and other processes [26].

In *Paragonimus* spp., expanded gene families included heat shock proteins (HSPs), major vault proteins, and multidrug resistance proteins that play roles in maintaining cellular homeostasis under stress conditions. HSPs of flatworm parasites play a key role as molecular chaperones in the maintenance of protein homeostasis. They also are immunogenic and immunomodulatory. HSP is the most abundant family of proteins in the immature and mature egg of *Schistosoma mansoni*, and in the miracidium [27] and is highly abundant in the tegument of the adult schistosome [28]. In addition, HSP is abundant in the excretory/secretory products of the adult *Schistosoma japonicum* blood fluke [29]. HSP stimulates diverse immune cells, eliciting release of pro- and anti-inflammatory cytokines [30], binds human LDL (the purpose of which is unknown but may be associated with transport of apoprotein B or in lipid trafficking [31]) and, given these properties, HSP represents a promising vaccine and diagnostic candidate [32]. Vaults, ribonucleoprotein complexes, are highly conserved in eukaryotes. Although their exact function remains unclear, it may be associated with multidrug resistance phenotypes and with signal transduction. In *S. mansoni*, up-regulation of major vault protein has been observed during the transition from cercaria to schistosomulum and in praziquantel-resistant adult worms [33]. ATP-binding cassette transporters (ABC transporters) are essential components of cellular physiological machinery, and some ABC

transporters, including P-glycoproteins, pump toxins and xenobiotics out of the cell. Overexpression of P-glycoprotein has been reported in a praziquantel-resistant *S. mansoni* [34].

Tetraspanin sequence evolution in *P. kellicotti*

We searched for genes that evolved under positive selection in the four *Paragonimus* spp. based on the non-synonymous to synonymous substitution rate ratio (d_N/d_S). We conducted the branch-site test of positive selection to identify adaptive gene variants that became fixed in each species [35] (**Supplementary Table S3**). A tetraspanin from *P. kellicotti* (PKEL_00573) reached statistical significance after correction for multiple testing (d_N/d_S = 9.9, FDR = 0.018). Tetraspanins are small integral proteins bearing four transmembrane domains which form two extracellular loops [36]. In trematodes, they are major components of the tegument at the host-parasite interface [37], are highly immunogenic vaccine antigens [38, 39], and may play a role in immune evasion [40]. In the tetraspanin sequence of *P. kellicotti*, we detected six amino acid sites under positive selection (**Supplementary Figure S2**). Five of the six sites were predicted to be located within the extracellular loops believed to interact with the immune system of the host. A similar pattern of positive selection within regions that code for extracellular loops has been reported in tetraspanin-23 from African *Schistosoma* species [41].

Gene phylogeny analysis identifies functions conserved and specific to fluke groups

We classified orthologous groups (OGs) based on phelogenetic distribution of proteins from each of the 21 species (**Figure 3B)**. Complete gene counts and lists per species and per OG are provided in **Supplementary Table S4**. These results were parsed to identify the OGs containing members among the platyhelminth species, and those that were conserved across all members of each group (lung, liver, and blood flukes, and other platyhelminth species (**Figure 5A**). This analysis identified 256 OGs that were conserved among, and exclusive to, the lung flukes (**Figures 5A and 5B**). The lung fluke-conserved and -specific genes were significantly enriched for several gene ontology (GO) terms (**Table 2**; using *P. miyazakii* genes to test significance), most of which were related to peptidase activity (including serine proteases which are involved in host tissue invasion, anticoagulation, and immune evasion [42]), as well as “iron binding” (which may be related to novel iron acquisition mechanisms from host tissue, which is not well understood in most metazoan parasites, but has been described in schistosomes [43]). Lung (adult) stage RNA-Seq datasets were collected for each of the four lung

fluke species (accessions in **Supplementary Table S1**), and reads were mapped to each of their respective genomes. Based on the 1:1 gene orthologs (as defined by the previously described OG dataset), the orthologous genes across the lung flukes had consistent adult-stage gene expression levels, with Pearson correlations ranging from 0.72 to 0.85 (**Figure 6A**, **6B**).

Expansion of unique aspartic proteases (including those predicted to be retropepsins) and other peptidases in the lung flukes may be associated with digestion of ingested blood, given the key role of this category of hydrolases and their inhibitors in nutrition and digestion of hemoglobin by schistosomes, and indeed other blood-feeding worms including hookworms [44, 45]. Given that pulmonary hemorrhage and hemoptysis are cardinal signs of lung fluke infection, it can be anticipated that the lung flukes ingest host blood when localized at the ulcerous lesion induced in the pulmonary parenchyma by infection. Overall, protease counts across species were similar (**Figure 4C**) although *P. kellicotti* had substantially fewer protease inhibitors compared to the other *Paragonimus* species (34 vs 57, 62 and 66), *F. hepatica* (61) and *S. mansoni* (55). Protease inhibitors in flukes are thought to be important for creating a safe environment for the parasite inside the host by inhibiting and regulating protease activity and immunomodulation [91], so this may suggest a novel host interaction strategy by *P. kellicotti*.

Analysis of the adult-stage gene expression levels of the discrete protease classes (**Supplementary Figure S3**) did not identify substantial differences among the *Paragonimus* species, except for a lower expression of threonine proteases in *P. kellicotti.* During the adult stage, cysteine proteases in all *Paragonimus* species exhibited significantly higher expression overall compared to *F. hepatica*, but similar expression levels to *S. mansoni.* A previous study identified immunodominant excretory-secretory cysteine proteases of adult *Paragonimus westermani* involved in immune evasion [46] and another study identified critical roles for excretory-secretory cysteine proteases during tissue invasion by newly excysted metacercariae of *P. westermani* [47]. The rapid diversification and critical host-interaction functions of the proteases highlights their importance, both in terms of understanding *Paragonimus* biology and in terms of identifying targets for control.

Functional enrichment analysis among the lung, liver and blood fluke conserved-and-exclusive OGs (**Figure 5C**) indicated that each family of fluke has evolved a distinct set of aspartic peptidases, trematode eggshell synthesis genes and saposin-like genes (which interact with lipids and are strongly immunogenic during fascioliasis [48]). The lung flukes, meanwhile, have uniquely expanded sets of serine proteases, as well as other

genes families with functions including FAR1 DNA binding (a class of proteins which are important secreted host-interacting proteins in some parasitic nematodes [49]), fatty-acid binding, and ferritin-like functions (intracellular proteins involved in iron metabolism, localized in vitelline follicles and eggs [50]).

Treatments, vaccine targets and diagnostics

The World Health Organization (WHO) currently recommends the use of praziquantel or, as a backup, triclabendazole for the treatment of paragonimiasis; both are highly effective for curing infections [51]. However, there are concerns about the development of resistance to these drugs; triclabendazole resistance of *P. westermani* was reported in a human case from Korea [52]. Furthermore, there is widespread resistance to triclabendazole in liver flukes in cattle in Australia and South America [53], and praziquantel resistance is anticipated in the future due to its widespread use as a single treatment for schistosomiasis, a worrisome situation which has encouraged the search for novel drugs [54]. The comparative analysis presented here identifies valuable putative protein targets for drug development, including *Paragonimus*-specific proteins and trematode-conserved proteins which do not share orthology to human proteins. The protein annotation data available in **Supplementary Table S2** also will enable prioritization including biological functional annotations [55, 56], protein weight and pi predictions [57], predictions of signal peptides and transmembrane domains [58] and cellular compartment localization [55], and sequence similarity matches to targets in the CHEMBL database [59]. This information can provide a starting point for future bioinformatic prioritization and drug testing (**Supplementary Tables S2** and **S3**).

Vaccination to prevent future infections would offer an attractive alternative to treatment, but development of vaccine protection against trematode infection has so far been unsuccessful and is unlikely to be practical for paragonimiasis in the near future [60]. However, the complete genome sequences and comparative analysis of the gene sets presented here provide valuable resources for future vaccine target development.

Pulmonary paragonimiasis is frequently mistaken for tuberculosis or pneumonia, and often patients do not shed eggs, which leads to false positive diagnoses of other conditions such as malaria or pneumonia [4, 61, 62]. This highlights a pressing need for accurate, rapid and affordable diagnostic approaches for paragonimiasis, a topic which has been the focus of numerous reports. We performed BLAST sequence similarity searches of previously identified *Paragonimus* diagnostic antigen targets among the four species (**Supplementary Figure**

S4). These included: (i) *P. westermani* and *P. pseudoheterotremus* cysteine proteases identified in two previous studies [63, 64] (matching to the same protein targets from both studies in *P. heterotremus* and *P. kellicotti*), one of which had high adult-stage expression levels in all four species [63]; (ii) three different tyrosine kinases (one of which was identified in two different studies, in *Clonorchis sinensis* and in *P. westermani* [65, 66]), all of which had relatively low gene expression levels in adult stages; (iii) a previously unannotated *P. heterotremus* ELISA antigen [67] with low expression across life cycle stages, which we now annotate as a saposin protein (which we found to rapidly evolve among flukes [**Figure 5C**], and which is strongly immunogenic in fascioliasis [48]); (iv) eggshell proteins of *P. westermani* [68], for which we now provide full-length sequences. We observed that this gene was conserved across and specific to the lung flukes, with lower gene expression in the young adult stage (*P. heterotremus*), but higher expression in the adult stages of all species; (v) among serodiagnostic *P. kellicotti* antigens based on a transcriptome assembly and proteomic evidence [69], we identified the top 10 of the 25 prioritized transcripts that best matched between the transcript sequence and the newly annotated draft genome of *P. kellicotti.* Thereafter, the full-length gene sequence in *P. kellicotti* was employed to query the other species. Several of these were highly expressed in the adult stage of all four species, including one that is fluke specific (PKEL_05597). However, not all of these had high sequence conservation across all species, with two only having weak hits in *P. heterotremus* (PKEL_00171 and PKEL_01872).

As a result of this newly developed genomic resource for the lung flukes, previously identified diagnostic targets were identified with full gene sequences across all four species. The complete gene sequences, conservation information and transcriptomic gene expression data for these target proteins can allow for optimization of the targets for diagnostic testing that is effective on species spanning the genus (**Supplementary Figure S5**). This is noteworthy given the absence of a standardized, commercially-available test for serodiagnosis for human paragonimiasis.

Conclusion

To substantially improve our understanding of the lung flukes at the molecular level, we sequenced, assembled, annotated and compared draft genomes of four species of *Paragonimus*, three from Asia (*P. miyazakii*, *P. westermani* from Japan, *P. heterotremus*) and one from North America (*P. kellicotti*), thereby providing novel and valuable genomic resources across these important parasites for the first time. We have

utilized these new resources to compare and analyze phylogenies, to identify gene sets and biological functions associated with parasitism in lung flukes, and to contribute a key resource for future investigation into host-parasite interactions for these poorly-understood agents of neglected tropical disease. Our identification of previously prioritized *Paragonimus* diagnostic markers in each of the four lung fluke species revealed that the same protein targets were identified in multiple studies, and hence the availability of full gene sequences now should facilitate diagnostic assays aiming for reactivity across all species of lung fluke. Overall, the novel genomic and transcriptomic resources developed here will be invaluable for research on paragonimiasis, guiding experimental design and generation of novel hypotheses.

Methods

Parasite specimens

Samples of DNA and RNA of *Paragonimus westermani* were sourced in Japan. *Paragonimus heterotremus* (LC strain, Vietnam) were recovered from a cat experimentally infected with metacercariae from Lai Chau province, northern Vietnam (70% ethanol preserved; whole worm). *Paragonimus miyazakii* metacercariae were recovered from freshwater crabs (*Geothelphusa dehaani*), collected in Shizuoka Prefecture, central Japan [15], and were raised to adulthood in rats. DNA and RNA samples were prepared for each of the (pre-)adult flukes recovered from the lungs and from the pleural and peritoneal cavities of experimentally infected rats. *Paragonimus kellicotti* adult worms for genome sequencing were recovered from the lungs of Mongolian gerbils infected in the laboratory with metacercariae recovered from Missouri crayfish [70].

Genome sequencing, assembly and annotation

DNA and RNA samples were collected from adult-stage parasites of four distinct *Paragonimus* species: *P. miyazakii* (Japan), *P. heterotremus* (LC strain, Vietnam), *P. kellicotti* (Missouri, USA) and *Paragonimus westermani* (Japan). Illumina DNA sequencing produced fragments, 3kb- and 8kb-insert whole-genome shotgun libraries, and PacBio reads were generated for *P. kellicotti*. The sequences were generated on the Illumina platform and assembled using Allpaths_LG [71]. Scaffolding was improved using an in-house tool called Pygap (gap closure tool), the Pyramid assembler with Illumina paired reads to close gaps and extend contigs, and L_RNA_scaffolder [72] which uses transcript alignments to improve contiguity. For *P. kellicotti*, Nanocorr was

used to perform error correction on the PacBio data and PBJelly was used to fill gaps and improve the Illumina allpaths assembly using the PacBio reads [73]. The nuclear genomes were annotated using the MAKER pipeline v2.31.8 [74]. Repetitive elements were softmasked with RepeatMasker v4.0.6 using a species-specific repeat library created by RepeatModeler v1.0.8, RepBase repeat libraries [75], and a list of known transposable elements provided by MAKER [74]. RNA-seq reads were aligned to their respective genome assemblies and assembled using StringTie v1.2.4 [76] (*P. miyazakii* samples collected from stages in the liver, peritoneal cavity [2 replicates], lung (adult) and pleural cavity; *P. heterotremus* samples from adults and young adults [2 replicates]; *P. westermani* [69] and *P. kellicotti* [77] adult-stage transcriptomic reads were retrieved from published reports). The resulting alignments and transcript assemblies were used by BRAKER [78] and MAKER pipelines, respectively, as extrinsic evidence. In addition, mRNA and EST sequences for each species were retrieved from NCBI, and were provided to MAKER as protein homology evidence along with protein sequences from UniRef100 [79] (Trematoda-specific, n=205,161) and WormBase ParaSite WBPS7 [80]. *Ab initio* gene predictions from BRAKER v2 [78] and AUGUSTUS v3.2.2 (trained by BRAKER and run within MAKER) were refined using the transcript and protein evidence. Previously unpredicted exons and UTRs were added, and split models were merged. The best-supported gene models were chosen based on Annotation Edit Distance (AED) [81]. To reduce false positives, gene predictions without supporting evidence were excluded in the final annotation build, with the exception of those encoding Pfam domains, as detected by InterProScan v5.19 [55]. These Pfam encoding domains were rescued in order to improve the annotation accuracy overall by balancing sensitivity and specificity [74, 82]. Gene products were named using PANNZER2 [83] and sma3s v2 [84]. **Supplementary Table S1** provides details of database accessions for the genomes. The completeness of annotated gene sets was assessed using BUSCO v3.0, eukaryota_odb9 [17]. Gene Ontology (GO), KEGG and protease annotations were performed using InterProScan v5.19 [55], GhostKOALA [56], and MEROPS [85], respectively. ExPASy was used to perform protein weight and pi predictions [57], SignalP was used to predict predictions signal peptides and transmembrane domains [58], and gene product localization was predicted using the “cellular component” Gene Ontology annotations provided by InterProScan [55].

Functional enrichment testing was performed using GOSTATS [86] for GO enrichment and negative binomial distribution tests for InterPro domain enrichment (minimum 3 annotated genes required for significant enrichment). Ribosomal RNAs and tRNAs were annotated using RNAmmer v1.2.1 [87] and tRNAscan-SE v1.23

[88], respectively. Genome characteristics and statistics including CDS, numbers and lengths of genes, exons and introns were defined using the longest complete mRNA (with start and stop codon) for each gene. Across the four species of *Paragonimus*, complete mRNAs were found for an average of 86.2% of all annotated genes.

Assembly of the mitochondrial genome of *P. kellicotti* was achieved using CANU [89] to align PacBio long-reads, followed by error-correction using Pilon [90].

MUMmer v4.0 [91] was used to estimate the level of genetic divergence between *P. westermani* samples from Japan and India. Nucmer was run first to generate genome alignments using draft assembly sequences. Dnadiff was then used to calculate the average sequence identity between the genomes considering only 1-to-1 alignments.

<u>Transcriptome datasets and gene functional annotations</u>

RNA-seq datasets were trimmed for adapters [92] and aligned [93] to their respective genome assemblies, and gene expression levels (FPKM) were quantified per gene per sample in each of the four species [94]. Interpro domains and Gene Ontology (GO) terms [55], KEGG enzymes [56], and protease [85] annotations of the genes were used to identify putative functions of genes of interest and perform pathway enrichment [86]. All raw RNA-Seq fastq files were uploaded to the NCBI Sequence Read Archive (SRA [95]), and complete sample metadata and accession information are provided in **Supplementary Table S1**. **Supplementary Table S2** provides, for each of the species, complete gene lists and gene expression levels for each of the RNA-Seq samples. Complete functional annotations for every gene are also provided for *P. miyazakii* in this table.

<u>Repeat analysis</u>

RepeatModeler v1.0.8 (with WU-BLAST as its search engine) was used to build, refine and classify consensus models of putative interspersed repeats for each species. With the resulting repeat libraries, genomic sequences were screened using RepeatMasker v4.0.6 in “slow search” mode to generate a detailed annotation of the interspersed and simple repeats. Per-copy distances to consensus were calculated (Kimura 2-parameter model, excluding CpG sites) and were plotted as repeat landscapes where divergence distribution reflected the activity of transposable elements (TE) on a relative time scale per genome using the calcDivergenceFromAlign.pl and createRepeatLandscape.pl scripts included in the RepeatMasker package.

Gene family evolution

Orthologous groups (OG) of genes of 21 species were inferred with OrthoFinder v1.1.4 [96] using the longest isoform for each gene (*Paragonimus* genome source information in **Supplementary Table S1**; Worm gene sets were retrieved from WormBase ParaSite in June 2017 [80]; Outgroup species gene sets were retrieved from Ensembl in June 2017 [97]). CAFE method [23] was employed to model gene gain and loss while accounting for the species' phylogenetic history based on an ultrametric species tree and the number of gene copies found in each species for each gene family. Birth-death (λ) parameters were estimated and the statistical significance of the observed family size differences among taxa were assessed. Results from OrthoFinder [96] were parsed to identify the OGs of interest based on conservation, including the lung fluke-conserved, liver fluke-conserved and blood fluke-conserved OGs and gene sets per species. **Supplementary Table S4** provides details of full OG counts per species and gene membership.

We used PosiGene [98] to search genome-wide for genes that evolved under positive selection based on the non-synonymous to synonymous substitution ratio. TMMOD [99] and Protter [100] were used for transmembrane helical topology prediction and visualization, respectively. We searched for genes that evolved under positive selection in the four *Paragonimus* spp. based on the non-synonymous to synonymous substitution rate ratio (d_N/d_S). We conducted the branch-site test of positive selection to identify adaptive gene variants that became fixed in each species [35].

Previously identified *Paragonimus* diagnostic antigen search

Nucleotide sequences (or, if unavailable, amino acid sequences) were retrieved from each of the cited publications (**Supplementary Figure S5**). Diamond blastx (nucleotides; v0.9.9.110) or Diamond blastp (amino acids; v0.9.9.110) were used to identify the top hit gene in each *Paragonimus* genome annotation (default settings). The best BLAST E-value was used to identify the top match, followed by top bitscore, length and % ID in the case of ties. For the top 25 *P. kellicotti* immunodominant antigen transcripts identified in McNulty et al, 2014 [77], matches were identified between the assembled transcript and the annotated gene. For the other three species, the BLAST searches are performed against the identified *P. kellicotti* gene, and not the original transcript sequence.

RNAseq-based gene expression profiling

After adapter trimming using Trimmomatic v0.36 [92], RNA-seq reads were aligned to their respective genome assemblies using the STAR aligner [93] (2-pass mode, basic). All raw RNA-Seq fastq files were uploaded to the NCBI Sequence Read Archive (SRA [95]), and complete sample metadata and accession information are provided in **Supplementary Table S1**. Read fragments (read pairs or single reads) were quantified per gene per sample using featureCounts (version 1.5.1) [94]. FPKM (fragments per kilobase of gene length per million reads mapped) normalization was also performed. Pearson correlation-based RNA-Seq sample clustering was performed in R (using the hclust package, complete linkage).

Statistics

ANOVA analysis followed by Tukey's HSD post-hoc testing was performed to compare genome statistics and protease expression between species (**Figure 2**, **Supplementary Figure S3**). Because comparisons for the genome statistics by *t* tests involved large numbers of values, which can falsely indicate positive statistical significance, a random selection of 100 values from each species was used (excluding the upper and lower 1% of data to avoid outliers). Letter labels above the species indicate statistical groups, i.e., if two species share the same letter then they were not statistically significant from each other.

Availability of supporting data and materials

Genomic raw reads, genome assemblies, genome annotations, and raw transcriptomic (RNA-Seq) fastq files were uploaded and are available for download from the NCBI Sequence Read Archive (SRA [95]), with all accession numbers and relevant metadata provided in **Supplementary Table S1**. **Supplementary Table S2** provides, for each of the species, complete gene lists and gene expression levels for each of the RNA-Seq samples. All results of the genome-wide selection scan are provided in **Supplementary Table S3**. For each orthologous group identified, **Supplementary Table S4** provides complete gene lists, counts of genes per species, and average gene expression levels from each the *Paragonimus* transcriptome datasets described

above. All relevant software versions, and commands specifying the parameters used are presented in **Supplementary Text S1**.

Declarations

List of Abbreviations

FPKM - Fragments Per Kilobase of gene length per Million reads mapped (gene expression level)

OG - Orthologous Group

TE – Transposable Elements

Consent for Publication

Not Applicable.

Competing Interests

The authors declare that they have no competing interests.

Funding

Sequencing of the genomes was supported by the 'Sequencing the etiological agents of the Food-Borne Trematodiases' project (National Institutes of Health - National Human Genome Research Institute award number U54HG003079). Comparative genome analysis was funded by grants National Institutes of Health - National Institute of Allergy and Infectious Diseases AI081803 and National Institutes of Health - National Institute of General Medical Sciences GM097435 to M.M. Parasite material from Thailand was supported by Distinguished Research Professor Grant (WM), Thailand Research Fund (Grant no. DPG6280002).

Author's Contributions

1. **Conceptualization:** MM PJB.
2. **Formal analysis:** BAR YJC SNM HJ JM.
3. **Funding acquisition:** PJB MM.
4. **Methodology:** PJB PUF DB MM.

5. **Resources:** MM TA HS TLH PND WM DB PUF.
6. **Visualization:** BAR YJC.
7. **Writing – original draft:** BAR YJC MM.
8. **Writing – review & editing:** DB PJB PUF MM.

Acknowledgements

We gratefully acknowledge assistance provided by Xu Zhang and Kymberlie Pepin with genome assembly and annotation and by Rahul Tyagi for figure graphics. We thank Kurt Curtis for his help generating *P. kellicotti* parasite material.

References

1. Furst T, Keiser J and Utzinger J. Global burden of human food-borne trematodiasis: a systematic review and meta-analysis. Lancet Infect Dis. 2012;12 3:210-21. doi:10.1016/S1473-3099(11)70294-8.
2. Utzinger J, Becker SL, Knopp S, Blum J, Neumayr AL, Keiser J, et al. Neglected tropical diseases: diagnosis, clinical management, treatment and control. Swiss Med Wkly. 2012;142:w13727. doi:10.4414/smw.2012.13727.
3. Blair D. Paragonimiasis. Adv Exp Med Biol. 2014;766:115-52. doi:10.1007/978-1-4939-0915-5_5.
4. Furst T, Sayasone S, Odermatt P, Keiser J and Utzinger J. Manifestation, diagnosis, and management of foodborne trematodiasis. BMJ. 2012;344:e4093. doi:10.1136/bmj.e4093.
5. Lv S, Zhang Y, Steinmann P, Zhou XN and Utzinger J. Helminth infections of the central nervous system occurring in Southeast Asia and the Far East. Adv Parasitol. 2010;72:351-408. doi:S0065-308X(10)72012-1 [pii]
6. Sripa B, Kaewkes S, Intapan PM, Maleewong W and Brindley PJ. Food-borne trematodiases in Southeast Asia epidemiology, pathology, clinical manifestation and control. Adv Parasitol. 2010;72:305-50. doi:S0065-308X(10)72011-X [pii]
7. Liu Q, Wei F, Liu W, Yang S and Zhang X. Paragonimiasis: an important food-borne zoonosis in China. Trends Parasitol. 2008;24 7:318-23. doi:S1471-4922(08)00137-2 [pii]
8. Blair D, Xu ZB and Agatsuma T. Paragonimiasis and the genus Paragonimus. Adv Parasitol. 1999;42:113-222.
9. Blair D, Davis GM and Wu B. Evolutionary relationships between trematodes and snails emphasizing schistosomes and paragonimids. Parasitology. 2001;123:S229-S43. doi:Doi 10.1017/S003118200100837x.
10. Attwood SW, Upatham ES, Meng XH, Qiu DC and Southgate VR. The phylogeography of Asian Schistosoma (Trematoda: Schistosomatidae). Parasitology. 2002;125 Pt 2:99-112. doi:10.1017/s0031182002001981.
11. Doanh NP, Tu AL, Bui TD, Loan TH, Nonaka N, Horii Y, et al. Molecular and morphological variation of Paragonimus westermani in Vietnam with records of new second intermediate crab hosts and a new locality in a northern province. Parasitology. 2016;143 12:1639-46. doi:10.1017/S0031182016001219.
12. Oey H, Zakrzewski M, Narain K, Devi KR, Agatsuma T, Nawaratna S, et al. Whole-genome sequence of the oriental lung fluke Paragonimus westermani. Gigascience. 2019;8 1 doi:10.1093/gigascience/giy146.
13. Blair D, Chang Z, Chen M, Cui A, Wu B, Agatsuma T, et al. Paragonimus skrjabini Chen, 1959 (Digenea: Paragonimidae) and related species in eastern Asia: a combined molecular and morphological approach to identification and taxonomy. Syst Parasitol. 2005;60 1:1-21. doi:10.1007/s11230-004-1378-5.
14. Lane MA, Marcos LA, Onen NF, Demertzis LM, Hayes EV, Davila SZ, et al. Paragonimus kellicotti flukes in Missouri, USA. Emerg Infect Dis. 2012;18 8:1263-7. doi:10.3201/eid1808.120335.
15. Fischer PU and Weil GJ. North American paragonimiasis: epidemiology and diagnostic strategies. Expert Rev Anti-Infe. 2015;13 6:779-86. doi:10.1586/14787210.2015.1031745.
16. Blair D, Nawa Y, Mitreva M and Doanh PN. Gene diversity and genetic variation in lung flukes (genus Paragonimus). Trans R Soc Trop Med Hyg. 2016;110 1:6-12. doi:10.1093/trstmh/trv101.
17. Waterhouse RM, Seppey M, Simao FA, Manni M, Ioannidis P, Klioutchnikov G, et al. BUSCO applications from quality assessments to gene prediction and phylogenomics. Mol Biol Evol. 2017; doi:10.1093/molbev/msx319.
18. Choi YJ, Fontenla S, Fischer PU, Le TH, Costabile A, Blair D, et al. Adaptive Radiation of the Flukes of the Family Fasciolidae Inferred from Genome-Wide Comparisons of Key Species. Mol Biol Evol. 2020;37 1:84-99. doi:10.1093/molbev/msz204.
19. Stapley J, Santure AW and Dennis SR. Transposable elements as agents of rapid adaptation may explain the genetic paradox of invasive species. Mol Ecol. 2015;24 9:2241-52. doi:10.1111/mec.13089.
20. Schrader L and Schmitz J. The impact of transposable elements in adaptive evolution. Mol Ecol. 2018; doi:10.1111/mec.14794.

21. Chenais B, Caruso A, Hiard S and Casse N. The impact of transposable elements on eukaryotic genomes: from genome size increase to genetic adaptation to stressful environments. Gene. 2012;509 1:7-15. doi:10.1016/j.gene.2012.07.042.
22. Prasad PK, Tandon V, Biswal DK, Goswami LM and Chatterjee A. Phylogenetic reconstruction using secondary structures and sequence motifs of ITS2 rDNA of Paragonimus westermani (Kerbert, 1878) Braun, 1899 (Digenea: Paragonimidae) and related species. BMC Genomics. 2009;10 Suppl 3:S25. doi:10.1186/1471-2164-10-S3-S25.
23. Han MV, Thomas GW, Lugo-Martinez J and Hahn MW. Estimating gene gain and loss rates in the presence of error in genome assembly and annotation using CAFE 3. Mol Biol Evol. 2013;30 8:1987-97. doi:10.1093/molbev/mst100.
24. Ahn CS, Na BK, Chung DL, Kim JG, Kim JT and Kong Y. Expression characteristics and specific antibody reactivity of diverse cathepsin F members of Paragonimus westermani. Parasitol Int. 2015;64 1:37-42. doi:10.1016/j.parint.2014.09.012.
25. McNulty SN, Tort JF, Rinaldi G, Fischer K, Rosa BA, Smircich P, et al. Genomes of Fasciola hepatica from the Americas Reveal Colonization with Neorickettsia Endobacteria Related to the Agents of Potomac Horse and Human Sennetsu Fevers. PLoS Genet. 2017;13 1:e1006537. doi:10.1371/journal.pgen.1006537.
26. Jones MK, Gobert GN, Zhang L, Sunderland P and McManus DP. The cytoskeleton and motor proteins of human schistosomes and their roles in surface maintenance and host-parasite interactions. Bioessays. 2004;26 7:752-65. doi:10.1002/bies.20058.
27. Mathieson W and Wilson RA. A comparative proteomic study of the undeveloped and developed Schistosoma mansoni egg and its contents: the miracidium, hatch fluid and secretions. Int J Parasitol. 2010;40 5:617-28. doi:10.1016/j.ijpara.2009.10.014.
28. Sotillo J, Pearson M, Becker L, Mulvenna J and Loukas A. A quantitative proteomic analysis of the tegumental proteins from Schistosoma mansoni schistosomula reveals novel potential therapeutic targets. Int J Parasitol. 2015;45 8:505-16. doi:10.1016/j.ijpara.2015.03.004.
29. Liu F, Cui SJ, Hu W, Feng Z, Wang ZQ and Han ZG. Excretory/secretory proteome of the adult developmental stage of human blood fluke, Schistosoma japonicum. Mol Cell Proteomics. 2009;8 6:1236-51. doi:10.1074/mcp.M800538-MCP200.
30. Kolinski T, Marek-Trzonkowska N, Trzonkowski P and Siebert J. Heat shock proteins (HSPs) in the homeostasis of regulatory T cells (Tregs). Cent Eur J Immunol. 2016;41 3:317-23. doi:10.5114/ceji.2016.63133.
31. Pereira AS, Cavalcanti MG, Zingali RB, Lima-Filho JL and Chaves ME. Isoforms of Hsp70-binding human LDL in adult Schistosoma mansoni worms. Parasitol Res. 2015;114 3:1145-52. doi:10.1007/s00436-014-4292-z.
32. He S, Yang L, Lv Z, Hu W, Cao J, Wei J, et al. Molecular and functional characterization of a mortalin-like protein from Schistosoma japonicum (SjMLP/hsp70) as a member of the HSP70 family. Parasitol Res. 2010;107 4:955-66. doi:10.1007/s00436-010-1960-5.
33. Reis EV, Pereira RV, Gomes M, Jannotti-Passos LK, Baba EH, Coelho PM, et al. Characterisation of major vault protein during the life cycle of the human parasite Schistosoma mansoni. Parasitol Int. 2014;63 1:120-6. doi:10.1016/j.parint.2013.10.005.
34. Messerli SM, Kasinathan RS, Morgan W, Spranger S and Greenberg RM. Schistosoma mansoni P-glycoprotein levels increase in response to praziquantel exposure and correlate with reduced praziquantel susceptibility. Mol Biochem Parasitol. 2009;167 1:54-9. doi:10.1016/j.molbiopara.2009.04.007.
35. Yang Z. PAML 4: phylogenetic analysis by maximum likelihood. Mol Biol Evol. 2007;24 8:1586-91. doi:10.1093/molbev/msm088.
36. Huang S, Yuan S, Dong M, Su J, Yu C, Shen Y, et al. The phylogenetic analysis of tetraspanins projects the evolution of cell-cell interactions from unicellular to multicellular organisms. Genomics. 2005;86 6:674-84. doi:10.1016/j.ygeno.2005.08.004.
37. Chaiyadet S, Krueajampa W, Hipkaeo W, Plosan Y, Piratae S, Sotillo J, et al. Suppression of mRNAs encoding CD63 family tetraspanins from the carcinogenic liver fluke Opisthorchis viverrini results in distinct tegument phenotypes. Sci Rep. 2017;7 1:14342. doi:10.1038/s41598-017-13527-5.
38. Krautz-Peterson G, Debatis M, Tremblay JM, Oliveira SC, Da'dara AA, Skelly PJ, et al. Schistosoma mansoni Infection of Mice, Rats and Humans Elicits a Strong Antibody Response to a Limited Number

of Reduction-Sensitive Epitopes on Five Major Tegumental Membrane Proteins. PLoS Negl Trop Dis. 2017;11 1:e0005306. doi:10.1371/journal.pntd.0005306.
39. Tran MH, Pearson MS, Bethony JM, Smyth DJ, Jones MK, Duke M, et al. Tetraspanins on the surface of Schistosoma mansoni are protective antigens against schistosomiasis. Nat Med. 2006;12 7:835-40. doi:10.1038/nm1430.
40. Wu C, Cai P, Chang Q, Hao L, Peng S, Sun X, et al. Mapping the binding between the tetraspanin molecule (Sjc23) of Schistosoma japonicum and human non-immune IgG. PLoS One. 2011;6 4:e19112. doi:10.1371/journal.pone.0019112.
41. Sealey KL, Kirk RS, Walker AJ, Rollinson D and Lawton SP. Adaptive radiation within the vaccine target tetraspanin-23 across nine Schistosoma species from Africa. Int J Parasitol. 2013;43 1:95-103. doi:10.1016/j.ijpara.2012.11.007.
42. Yang Y, Wen Y, Cai YN, Vallee I, Boireau P, Liu MY, et al. Serine proteases of parasitic helminths. Korean J Parasitol. 2015;53 1:1-11. doi:10.3347/kjp.2015.53.1.1.
43. Glanfield A, McManus DP, Anderson GJ and Jones MK. Pumping iron: a potential target for novel therapeutics against schistosomes. Trends Parasitol. 2007;23 12:583-8. doi:10.1016/j.pt.2007.08.018.
44. Brindley PJ, Kalinna BH, Wong JY, Bogitsh BJ, King LT, Smyth DJ, et al. Proteolysis of human hemoglobin by schistosome cathepsin D. Mol Biochem Parasitol. 2001;112 1:103-12.
45. Williamson AL, Brindley PJ, Abbenante G, Prociv P, Berry C, Girdwood K, et al. Cleavage of hemoglobin by hookworm cathepsin D aspartic proteases and its potential contribution to host specificity. FASEB J. 2002;16 11:1458-60. doi:10.1096/fj.02-0181fje.
46. Lee EG, Na BK, Bae YA, Kim SH, Je EY, Ju JW, et al. Identification of immunodominant excretory-secretory cysteine proteases of adult Paragonimus westermani by proteome analysis. Proteomics. 2006;6 4:1290-300. doi:10.1002/pmic.200500399.
47. Na BK, Kim SH, Lee EG, Kim TS, Bae YA, Kang I, et al. Critical roles for excretory-secretory cysteine proteases during tissue invasion of Paragonimus westermani newly excysted metacercariae. Cell Microbiol. 2006;8 6:1034-46. doi:10.1111/j.1462-5822.2006.00685.x.
48. Caban-Hernandez K and Espino AM. Differential expression and localization of saposin-like protein 2 of Fasciola hepatica. Acta Trop. 2013;128 3:591-7. doi:10.1016/j.actatropica.2013.08.012.
49. Basavaraju SV, Zhan B, Kennedy MW, Liu Y, Hawdon J and Hotez PJ. Ac-FAR-1, a 20 kDa fatty acid- and retinol-binding protein secreted by adult Ancylostoma caninum hookworms: gene transcription pattern, ligand binding properties and structural characterisation. Mol Biochem Parasitol. 2003;126 1:63-71.
50. Jones MK, McManus DP, Sivadorai P, Glanfield A, Moertel L, Belli SI, et al. Tracking the fate of iron in early development of human blood flukes. Int J Biochem Cell Biol. 2007;39 9:1646-58. doi:10.1016/j.biocel.2007.04.017.
51. World Health Organization. 2019. Accessed August 25, 2019.
52. Kyung SY, Cho YK, Kim YJ, Park JW, Jeong SH, Lee JI, et al. A paragonimiasis patient with allergic reaction to praziquantel and resistance to triclabendazole: successful treatment after desensitization to praziquantel. Korean J Parasitol. 2011;49 1:73-7. doi:10.3347/kjp.2011.49.1.73.
53. Kelley JM, Elliott TP, Beddoe T, Anderson G, Skuce P and Spithill TW. Current Threat of Triclabendazole Resistance in Fasciola hepatica. Trends Parasitol. 2016; doi:10.1016/j.pt.2016.03.002.
54. Mader P, Rennar GA, Ventura AMP, Grevelding CG and Schlitzer M. Chemotherapy for Fighting Schistosomiasis: Past, Present and Future. ChemMedChem. 2018;13 22:2374-89. doi:10.1002/cmdc.201800572.
55. Jones P, Binns D, Chang HY, Fraser M, Li W, McAnulla C, et al. InterProScan 5: genome-scale protein function classification. Bioinformatics. 2014;30 9:1236-40. doi:10.1093/bioinformatics/btu031
56. Kanehisa M, Sato Y and Morishima K. BlastKOALA and GhostKOALA: KEGG Tools for Functional Characterization of Genome and Metagenome Sequences. J Mol Biol. 2016;428 4:726-31. doi:10.1016/j.jmb.2015.11.006.
57. Artimo P, Jonnalagedda M, Arnold K, Baratin D, Csardi G, de Castro E, et al. ExPASy: SIB bioinformatics resource portal. Nucleic Acids Res. 2012;40 Web Server issue:W597-603. doi:10.1093/nar/gks400.
58. Almagro Armenteros JJ, Tsirigos KD, Sonderby CK, Petersen TN, Winther O, Brunak S, et al. SignalP 5.0 improves signal peptide predictions using deep neural networks. Nat Biotechnol. 2019;37 4:420-3. doi:10.1038/s41587-019-0036-z.

59. Mendez D, Gaulton A, Bento AP, Chambers J, De Veij M, Felix E, et al. ChEMBL: towards direct deposition of bioassay data. Nucleic Acids Res. 2019;47 D1:D930-D40. doi:10.1093/nar/gky1075.
60. Stutzer C, Richards SA, Ferreira M, Baron S and Maritz-Olivier C. Metazoan Parasite Vaccines: Present Status and Future Prospects. Front Cell Infect Microbiol. 2018;8:67. doi:10.3389/fcimb.2018.00067.
61. Radzikowska E, Chabowski M and Bestry I. Tuberculosis mimicry. Eur Respir J. 2006;27 3:652; author reply doi:10.1183/09031936.06.00121205.
62. Eapen S, Espinal E and Firstenberg M. Delayed diagnosis of paragonimiasis in Southeast Asian immigrants: A need for global awareness. 2018;4 2:173-7. doi:10.4103/ijam.Ijam_2_18.
63. Yang SH, Park JO, Lee JH, Jeon BH, Kim WS, Kim SI, et al. Cloning and characterization of a new cysteine proteinase secreted by Paragonimus westermani adult worms. Am J Trop Med Hyg. 2004;71 1:87-92.
64. Yoonuan T, Nuamtanong S, Dekumyoy P, Phuphisut O and Adisakwattana P. Molecular and immunological characterization of cathepsin L-like cysteine protease of Paragonimus pseudoheterotremus. Parasitol Res. 2016;115 12:4457-70. doi:10.1007/s00436-016-5232-x.
65. Kim SH and Bae YA. Lineage-specific expansion and loss of tyrosinase genes across platyhelminths and their induction profiles in the carcinogenic oriental liver fluke, Clonorchis sinensis. Parasitology. 2017;144 10:1316-27. doi:10.1017/S003118201700083X.
66. Bae YA, Kim SH, Ahn CS, Kim JG and Kong Y. Molecular and biochemical characterization of Paragonimus westermani tyrosinase. Parasitology. 2015;142 6:807-15. doi:10.1017/S0031182014001942.
67. Pothong K, Komalamisra C, Kalambaheti T, Watthanakulpanich D, Yoshino TP and Dekumyoy P. ELISA based on a recombinant Paragonimus heterotremus protein for serodiagnosis of human paragonimiasis in Thailand. Parasit Vectors. 2018;11 1:322. doi:10.1186/s13071-018-2878-5.
68. Bae YA, Kim SH, Cai GB, Lee EG, Kim TS, Agatsuma T, et al. Differential expression of Paragonimus westermani eggshell proteins during the developmental stages. Int J Parasitol. 2007;37 3-4:295-305. doi:10.1016/j.ijpara.2006.10.006.
69. Li BW, McNulty SN, Rosa BA, Tyagi R, Zeng QR, Gu KZ, et al. Conservation and diversification of the transcriptomes of adult Paragonimus westermani and P. skrjabini. Parasit Vectors. 2016;9:497. doi:10.1186/s13071-016-1785-x.
70. Fischer PU, Curtis KC, Marcos LA and Weil GJ. Molecular characterization of the North American lung fluke Paragonimus kellicotti in Missouri and its development in Mongolian gerbils. Am J Trop Med Hyg. 2011;84 6:1005-11. doi:10.4269/ajtmh.2011.11-0027.
71. Gnerre S, Maccallum I, Przybylski D, Ribeiro FJ, Burton JN, Walker BJ, et al. High-quality draft assemblies of mammalian genomes from massively parallel sequence data. Proc Natl Acad Sci U S A. 2011;108 4:1513-8. doi:10.1073/pnas.1017351108.
72. Xue W, Li JT, Zhu YP, Hou GY, Kong XF, Kuang YY, et al. L_RNA_scaffolder: scaffolding genomes with transcripts. BMC Genomics. 2013;14:604. doi:10.1186/1471-2164-14-604.
73. English AC, Richards S, Han Y, Wang M, Vee V, Qu J, et al. Mind the gap: upgrading genomes with Pacific Biosciences RS long-read sequencing technology. PLoS One. 2012;7 11:e47768. doi:10.1371/journal.pone.0047768.
74. Holt C and Yandell M. MAKER2: an annotation pipeline and genome-database management tool for second-generation genome projects. BMC Bioinformatics. 2011;12:491. doi:10.1186/1471-2105-12-491.
75. Bao W, Kojima KK and Kohany O. Repbase Update, a database of repetitive elements in eukaryotic genomes. Mob DNA. 2015;6:11. doi:10.1186/s13100-015-0041-9.
76. Pertea M, Pertea GM, Antonescu CM, Chang TC, Mendell JT and Salzberg SL. StringTie enables improved reconstruction of a transcriptome from RNA-seq reads. Nat Biotechnol. 2015;33 3:290-5. doi:10.1038/nbt.3122.
77. McNulty SN, Fischer PU, Townsend RR, Curtis KC, Weil GJ and Mitreva M. Systems biology studies of adult paragonimus lung flukes facilitate the identification of immunodominant parasite antigens. PLoS Negl Trop Dis. 2014;8 10:e3242. doi:10.1371/journal.pntd.0003242.
78. Hoff KJ, Lange S, Lomsadze A, Borodovsky M and Stanke M. BRAKER1: Unsupervised RNA-Seq-Based Genome Annotation with GeneMark-ET and AUGUSTUS. Bioinformatics. 2016;32 5:767-9. doi:10.1093/bioinformatics/btv661.

79. The UniProt C. UniProt: the universal protein knowledgebase. Nucleic Acids Res. 2017;45 D1:D158-D69. doi:10.1093/nar/gkw1099.
80. Howe KL, Bolt BJ, Shafie M, Kersey P and Berriman M. WormBase ParaSite - a comprehensive resource for helminth genomics. Mol Biochem Parasitol. 2017;215:2-10. doi:10.1016/j.molbiopara.2016.11.005.
81. Eilbeck K, Moore B, Holt C and Yandell M. Quantitative measures for the management and comparison of annotated genomes. BMC Bioinformatics. 2009;10:67. doi:10.1186/1471-2105-10-67.
82. Campbell MS, Law M, Holt C, Stein JC, Moghe GD, Hufnagel DE, et al. MAKER-P: a tool kit for the rapid creation, management, and quality control of plant genome annotations. Plant Physiol. 2014;164 2:513-24. doi:10.1104/pp.113.230144.
83. Koskinen P, Toronen P, Nokso-Koivisto J and Holm L. PANNZER: high-throughput functional annotation of uncharacterized proteins in an error-prone environment. Bioinformatics. 2015;31 10:1544-52. doi:10.1093/bioinformatics/btu851.
84. Casimiro-Soriguer CS, Munoz-Merida A and Perez-Pulido AJ. Sma3s: A universal tool for easy functional annotation of proteomes and transcriptomes. Proteomics. 2017;17 12 doi:10.1002/pmic.201700071.
85. Rawlings ND, Barrett AJ and Finn R. Twenty years of the MEROPS database of proteolytic enzymes, their substrates and inhibitors. Nucleic Acids Res. 2016;44 D1:D343-50. doi:10.1093/nar/gkv1118.
86. Falcon S and Gentleman R. Using GOstats to test gene lists for GO term association. Bioinformatics. 2007;23 2:257-8. doi:10.1093/bioinformatics/btl567.
87. Lagesen K, Hallin P, Rodland EA, Staerfeldt HH, Rognes T and Ussery DW. RNAmmer: consistent and rapid annotation of ribosomal RNA genes. Nucleic Acids Res. 2007;35 9:3100-8. doi:10.1093/nar/gkm160.
88. Lowe TM and Eddy SR. tRNAscan-SE: a program for improved detection of transfer RNA genes in genomic sequence. Nucleic Acids Res. 1997;25 5:955-64.
89. Koren S, Walenz BP, Berlin K, Miller JR, Bergman NH and Phillippy AM. Canu: scalable and accurate long-read assembly via adaptive k-mer weighting and repeat separation. Genome Res. 2017;27 5:722-36. doi:10.1101/gr.215087.116.
90. Walker BJ, Abeel T, Shea T, Priest M, Abouelliel A, Sakthikumar S, et al. Pilon: an integrated tool for comprehensive microbial variant detection and genome assembly improvement. PLoS One. 2014;9 11:e112963. doi:10.1371/journal.pone.0112963.
91. Marcais G, Delcher AL, Phillippy AM, Coston R, Salzberg SL and Zimin A. MUMmer4: A fast and versatile genome alignment system. PLoS Comput Biol. 2018;14 1:e1005944. doi:10.1371/journal.pcbi.1005944.
92. Bolger AM, Lohse M and Usadel B. Trimmomatic: a flexible trimmer for Illumina sequence data. Bioinformatics. 2014;30 15:2114-20. doi:10.1093/bioinformatics/btu170.
93. Dobin A, Davis CA, Schlesinger F, Drenkow J, Zaleski C, Jha S, et al. STAR: ultrafast universal RNA-seq aligner. Bioinformatics. 2013;29 1:15-21. doi:10.1093/bioinformatics/bts635.
94. Liao Y, Smyth GK and Shi W. featureCounts: an efficient general purpose program for assigning sequence reads to genomic features. Bioinformatics. 2014;30 7:923-30. doi:10.1093/bioinformatics/btt656.
95. Leinonen R, Sugawara H, Shumway M and on behalf of the International Nucleotide Sequence Database C. The Sequence Read Archive. Nucleic Acids Res. 2011;39 Database issue:D19-D21. doi:10.1093/nar/gkq1019.
96. Emms DM and Kelly S. OrthoFinder: solving fundamental biases in whole genome comparisons dramatically improves orthogroup inference accuracy. Genome Biol. 2015;16:157. doi:10.1186/s13059-015-0721-2.
97. Zerbino DR, Achuthan P, Akanni W, Amode MR, Barrell D, Bhai J, et al. Ensembl 2018. Nucleic Acids Res. 2018;46 D1:D754-D61. doi:10.1093/nar/gkx1098.
98. Sahm A, Bens M, Platzer M and Szafranski K. PosiGene: automated and easy-to-use pipeline for genome-wide detection of positively selected genes. Nucleic Acids Res. 2017;45 11:e100. doi:10.1093/nar/gkx179.
99. Kahsay RY, Gao G and Liao L. An improved hidden Markov model for transmembrane protein detection and topology prediction and its applications to complete genomes. Bioinformatics. 2005;21 9:1853-8. doi:10.1093/bioinformatics/bti303.

100. Omasits U, Ahrens CH, Muller S and Wollscheid B. Protter: interactive protein feature visualization and integration with experimental proteomic data. Bioinformatics. 2014;30 6:884-6. doi:10.1093/bioinformatics/btt607.

Figure Captions

Figure 1. Comparisons of the overall content of the assembled *Paragonimus* genome assemblies. Comparisons are based on (**A**) length (including statistics for other sequenced trematode genomes) and (**B**) Repeat landscapes, measured using the Kimura substitution level, which indicates how much a repeat sequence has degenerated since its incorporation into the genome (i.e., how recently the repeat sequence was added). The high peak at the far left of *P. kellicotti* indicates a recent incorporation or active transposable element activity.

Figure 2: Comparison of genome annotation characteristics and attributes among several species of flatworms. Attributes characterized included (**A**) Full gene lengths, including coding and noncoding sequences, (**B**) Average intron lengths per gene, (**C**) Number of exons per gene, and (**D**) Coding sequence (CDS) length per exon. *P* values and letter groupings indicating significant differences among species, as calculated using ANOVA with Tukey's HSD post-hoc test.

Figure 3. Clustering of *Paragonimus* species. (**A**) Mitochondrial whole genome-based phylogeny, including previously-sequenced *Paragonimus* mitochondrial genomes (with accessions indicated). (**B**) Species clustering based on single-member OPF sequences. 262,720 genes (85% of all genes across the species) were assigned to 17,953 OPFs; 2,493 genes are in 326 species-specific OPFs.

Figure 4. Gene-family dynamics among platyhelminth species. (**A**) Rapidly evolving families of interest are quantified at each stage of the phylogeny, including genes gained (blue) and lost (red) relative to other species. The number of rapidly evolving genes are indicated in parentheses. (**B**) Functionally annotated gene families of interest that displayed most pronounced differential expansions or contractions. (**C**) Overall protease and protease inhibitor abundance per species.

Figure 5. Orthologous Group (OG) distribution analysis. (**A**) OGs identified among groups of flukes. The OGs conserved in at least one of the species from each group are indicated in black, and the OGs conserved among all the species in the overlapping groups are indicated in red. (**B**) Counts of OGs among the four *Paragonimus*

species, with *Paragonimus*-specific gene sets indicated in red text. The 256 *Paragonimus* conserved-and-specific genes are indicated with highlight (Table 4). (**C**) Significant functional enrichment (Interpro domains) among the gene sets conserved among, and specific to, each major group of flukes (256, 758 and 270 OPFs in lung, liver and blood flukes, respectively), relative to the functions in the complete gene sets.

Figure 6: Analysis of gene expression data for species of lung flukes of the genus *Paragonimus*. (**A**) Comparison of adult-stage gene expression levels among 1:1 orthologs shared by *P. westermani* and *P. miyazakii*. Pearson correlation = 0.79. (**B**) Pearson correlation values between all lung fluke species for the adult-stage expression levels of all 1:1 orthologous genes.

Tables

Table 1: The draft genome of *Paragonimus*: assembly, size and annotation characteristics

Statistic	*Paragonimus miyazakii*	*Paragonimus heterotremus*	*Paragonimus kellicotti*	*Paragonimus westermani* (Japan)	*Paragonimus westermani* (India)
**Assembly statistics**					
Total genome length (Mb)	915.8	841.2	696.5	923.3	922.8
Number of contigs	22,318	27,557	29,377	22,477	30,455
Mean contig size (kb)	41	30.5	23.7	41.1	30.3
Median contig size (kb)	15.1	9.3	10.2	17.2	4.8
Max. contig size (kb)	919.8	715.6	826	829	809.4
N50 length (kb)	108.8	92.5	56.0	100.8	135.2
N50 number	2,320	2,506	3,316	2,664	1,943
**BUSCO completeness (303 genes, eukarota_odb9)**					
Complete, single copy	84.5%	82.5%	70.3%	88.78%	76.90%
Complete, duplicated	1.3%	0.0%	1.3%	1.32%	2.31%
Fragmented	7.6%	10.9%	15.2%	6.27%	14.85%
Missing	6.6%	6.6%	13.2%	3.63%	5.94%
**Gene statistics**					
Number of genes	12,652	12,490	12,853	12,072	12,771
Avg gene length (kb)	25.9	22.6	17.6	24.1	18.0
Avg CDS length (kb)	1.5	1.4	1.1	1.4	1.4
Avg intron length (kb)	4.2	4	3.6	4.2	4.0
Avg # exons per gene	6.7	6.2	5.3	6.3	5.2
% annotated InterPro	82%	85%	81%	87%	82%
% annotated KEGG	40%	41%	34%	43%	43%

Table 2. "Molecular Function" Gene Ontology terms enriched among *P. miyazakii* genes that are conserved among and exclusive to lung flukes.

GO ID	GO term name	P value	# Conserved and Specific	Total # in genome
GO:0004175	endopeptidase activity	5.2E-05	8	132
GO:0008236	serine-type peptidase activity	5.6E-05	6	67
GO:0017171	serine hydrolase activity	5.6E-05	6	67
GO:0004252	serine-type endopeptidase activity	1.6E-04	5	51
GO:0070011	peptidase activity, acting on L-amino acid peptides	6.1E-04	9	237
GO:0008233	peptidase activity	8.7E-04	9	249
GO:0004568	chitinase activity	2.1E-03	2	7
GO:0004190	aspartic-type endopeptidase activity	1.1E-02	2	16
GO:0070001	aspartic-type peptidase activity	1.1E-02	2	16
GO:0008199	ferric iron binding	1.1E-02	2	16

Click here to access/download;Figure;Figure 1.tif

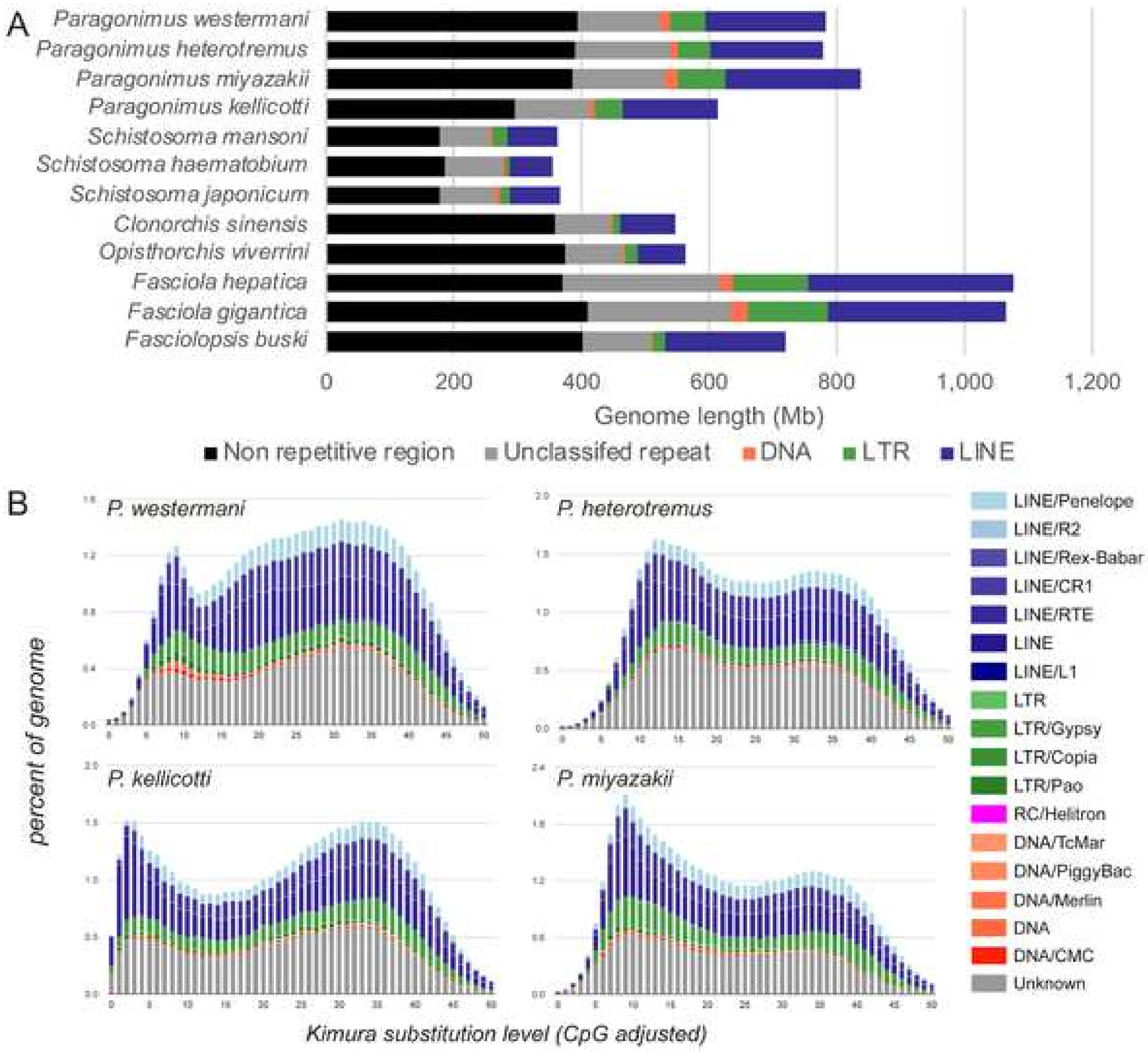

Figure 2

Click here to access/download;Figure;Figure 2.tif

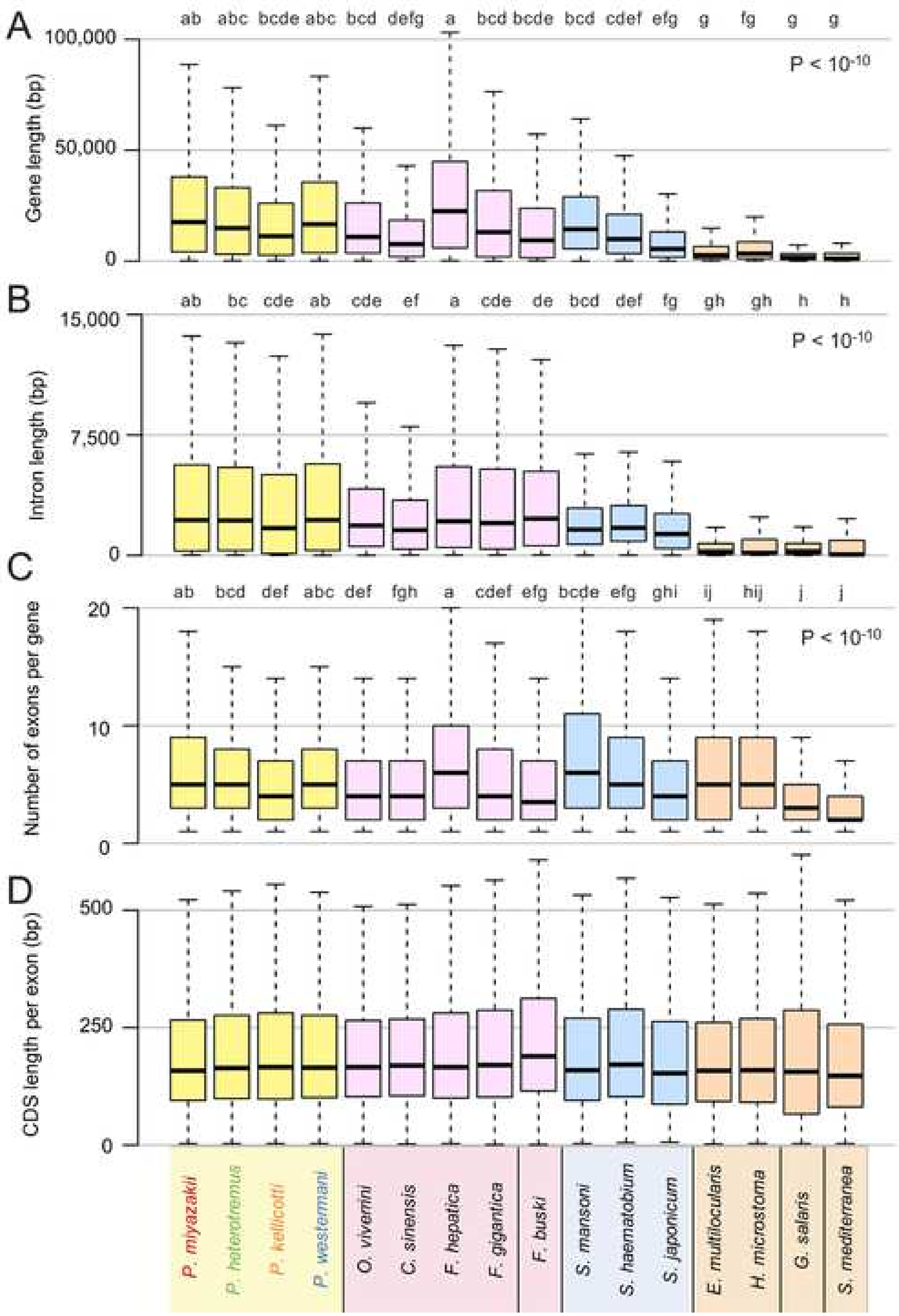

Figure 3

Click here to access/download;Figure;Figure 3.tif

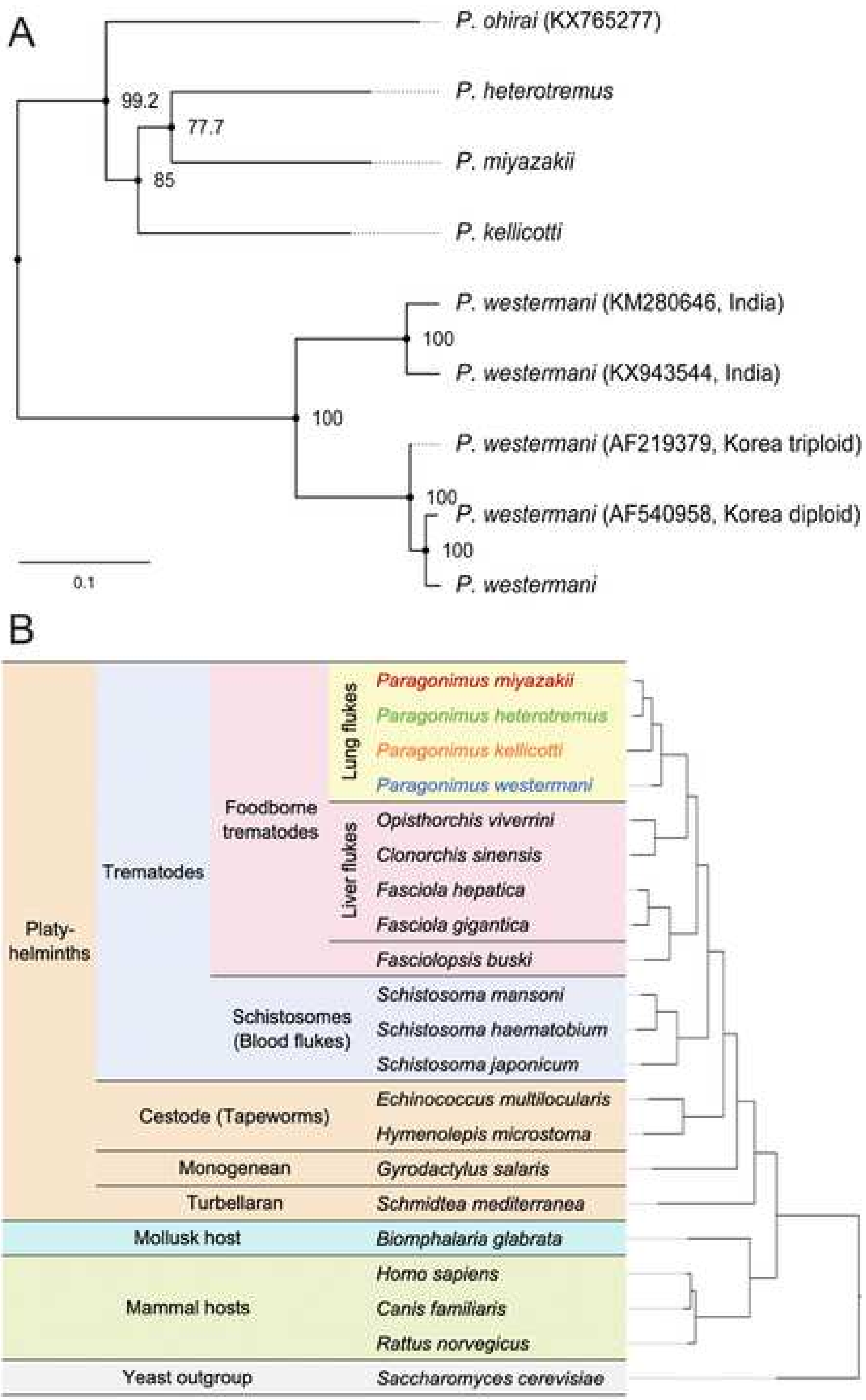

Click here to access/download;Figure;Figure 4.tif

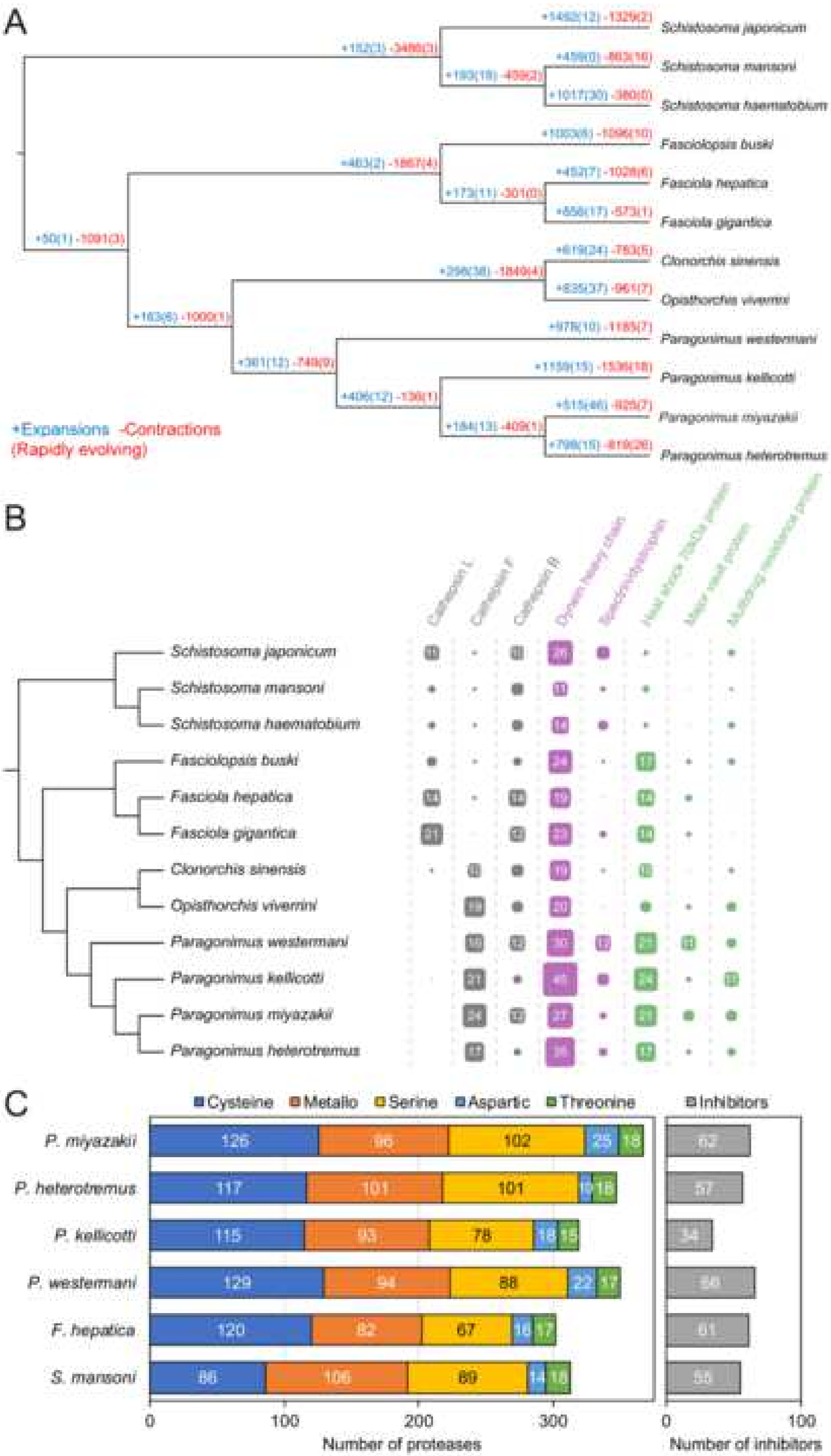

Figure 5

Click here to access/download;Figure;Figure 5.tif

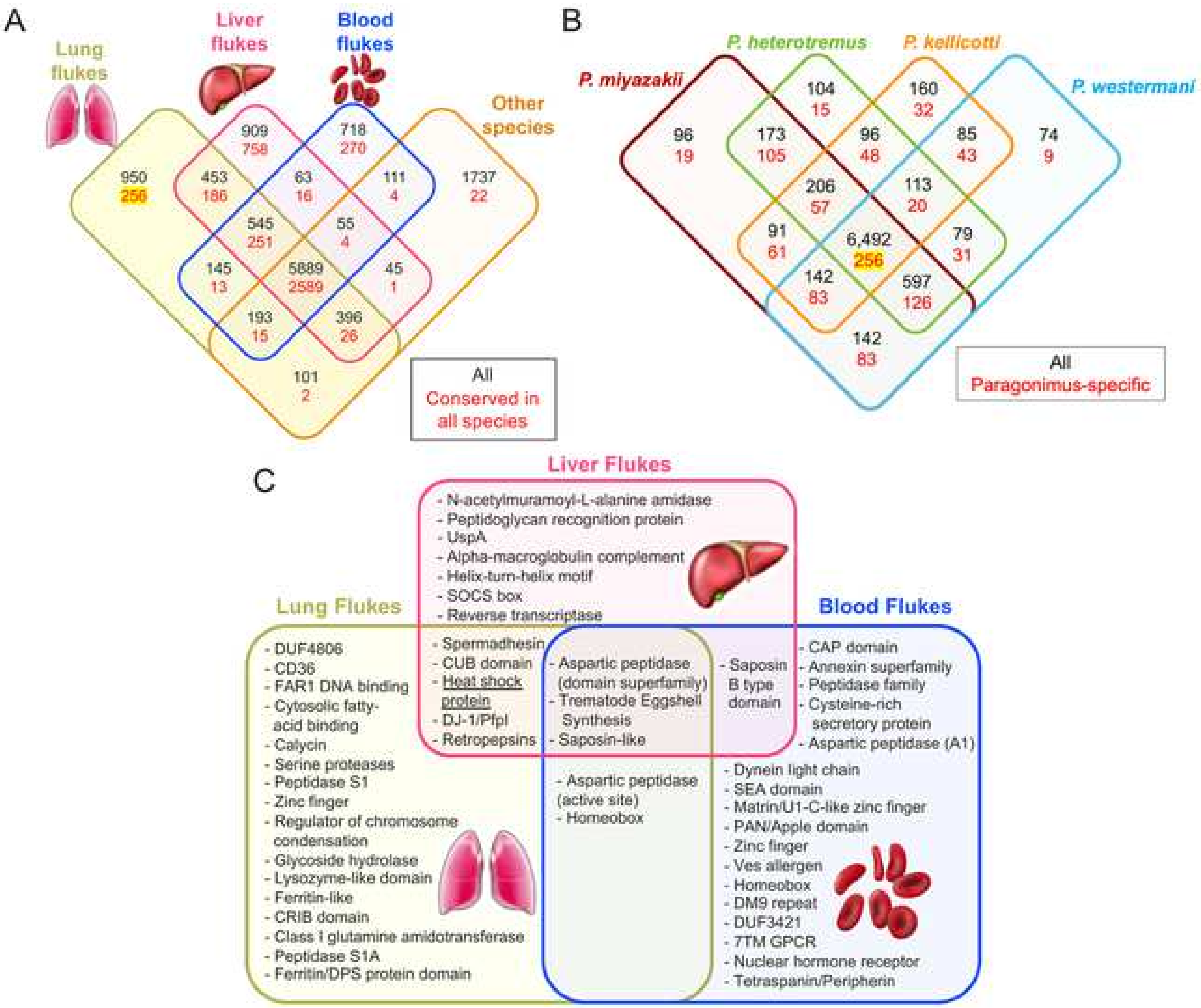

Figure 6 Click here to access/download;Figure;Figure 6 - REVISED.tif

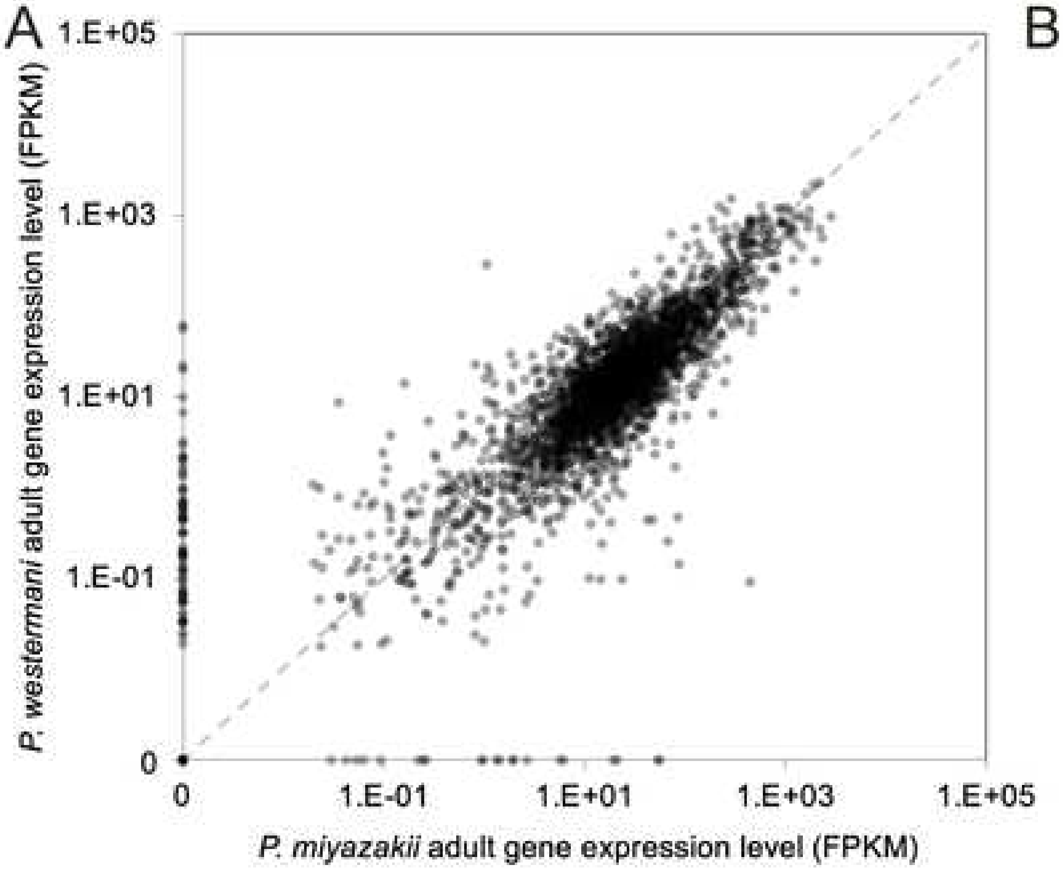

B

Adult stage 1:1 gene correlation

	P. miyazakii	*P. heterotremus*	*P. kellicotti*	*P. westermani*
P. miyazakii				
P. heterotremus	0.76			
P. kellicotti	0.72	0.75		
P. westermani	0.79	0.85	0.76	

Click here to access/download
Supplementary Material
Supplementary Information (Combined) V5 - REVISED.docx

Supp Table S1

Click here to access/download
Supplementary Material
Supp Table S1 - Accessions.xlsx

Click here to access/download

Supplementary Material

Supp Table S2 - Paragonimus expression data - REVISED.xlsx

Click here to access/download
Supplementary Material
Supp Table S3 - Genome-wide selection scan.xlsx

Supp Table S4

Click here to access/download
Supplementary Material
Supp Table S4 - OGs and FPKM.xlsx

Response to reviewers

Click here to access/download
Supplementary Material
Reviewer Responses for Gigascience R2_FINAL.docx

Personal Cover
Click here to access/download;Personal Cover;Gigascience Cover Letter R2.docx

Washington University in St. Louis
SCHOOL OF MEDICINE

MCDONNELL GENOME INSTITUTE

To
Dr. Nicole Nogoy
Editor
Gigascience

4/30/2020

Re: **GIGA-D-19-00411R1** revision

Dear Dr. Nogoy,

Thank you for inviting us to submit a revised version of our manuscript: "*Comparative genomics and transcriptomics of four* Paragonimus *species provide insights into lung fluke parasitism and pathogenesis*" (GIGA-D-19-00411R1).

We appreciate the suggestions for improving the manuscript. As our point-by-point response document shows we have addressed all of the editorial and reviewers' concerns and we have revised the manuscript in accordance with the recommendations.

We very much appreciate your and the efforts of the referee in recommending how to best revise this manuscript. We have followed the advice especially closely and are hopeful that you will find it suitable for publication in Gigascience as a Research Article.

Thank you for your consideration.
Yours sincerely,

M. Mitreva

Makedonka Mitreva, PhD

Professor, Department of Medicine and of Genetics,
Assistant Director, McDonnell Genome Institute,
Director, Center for Clinical Genomics of Microbial Systems,
Washington University School of Medicine
