# Supplementary material for: Comparative genomics and transcriptomics of 4 Paragonimus species provide insights into lung fluke parasitism and pathogenesis
Source: Gigascience. 2020 Jul 20;9(7):giaa073. doi: 10.1093/gigascience/giaa073 (PMC7370270; doi:10.1093/gigascience/giaa073)
Supplement: giaa073_Supplemental_Files [file giaa073_supplemental_files.zip › Supplementary Text S1.docx]

**Supplementary Information**

*Comparative genomics and transcriptomics of four Paragonimus species provide insights into lung fluke parasitism and pathogenesis*

Bruce A. Rosa^1^*, Young-Jun Choi^1^*, Samantha N. McNulty^2^, Hyeim Jung^1^, John Martin^1^, Takeshi Agatsuma^3^, Hiromu Sugiyama^4^, Thanh Hoa Le^5^, Pham Ngoc Doanh^6,7^, Wanchai Maleewong^8^, David Blair^9^, Paul J. Brindley^10^, Peter U. Fischer^1^, Makedonka Mitreva^1,2†^

^1^Department of Internal Medicine, Washington University School of Medicine, St. Louis, MO 63110, USA

^2^The McDonnell Genome Institute at Washington University, School of Medicine, St. Louis, MO 63108, USA

^3^Department of Environmental Health Sciences, Kochi Medical School, Oko, Nankoku City, Kochi 783-8505, Japan

^4^Laboratory of Helminthology, Department of Parasitology, National Institute of Infectious Diseases, Tokyo 162-8640, Japan

^5^Department of Immunology, Institute of Biotechnology, Vietnam Academy of Science and Technology, Hanoi, Vietnam

^6^Institute of Ecology and Biological Resources, Vietnam Academy of Science and Technology, Hanoi, Vietnam

^7^Graduate University of Science and Technology, Vietnam Academy of Science and Technology, Hanoi, Vietnam

^8^Research and Diagnostic Center for Emerging Infectious Diseases, Khon Kaen University, Khon Kaen, Thailand, Department of Parasitology, Faculty of Medicine, Khon Kaen University, Khon Kaen, Thailand

^9^College of Marine and Environmental Sciences, James Cook University, Townsville, Queensland 4811, Australia

^10^Departments of Microbiology, Immunology and Tropical Medicine, and Research Center for Neglected Diseases of Poverty, and Pathology School of Medicine & Health Sciences, George Washington University, Washington, DC 20037, USA

*Authors contributed equally to this work

^†^Correspondence should be addressed to Makedonka Mitreva. Tel. +1-314-285-2005,

Fax +1-314-286-1800, Email: [mmitreva@wustl.edu](mailto:mmitreva@wustl.edu)

**Supplementary Text S1. Commands and parameters for analyses**

**1. Nuclear genome assembly and sequence divergence estimation**

PrepareAllPathsInputs.pl DATA_DIR=<full path of data directory> PLOIDY=2 IN_GROUPS_CSV=<in groups file> IN_LIBS_CSV=<in libs file> PICARD_TOOLS_DIR=<picard tools directory>

blat -t=dna -q=rna -maxIntron=100000 -noHead <assembly.fasta> <rnaseq.fasta> <output.psl>

L_RNA_scaffolder -d <path to L_RNA_scaffolder programs folder> -i <blat psl file> -j <assembly.fasta> -o <output dir>

PBJelly was run as described in the documentation included with the download (from: https://sourceforge.net/projects/pb-jelly/) with the protocol.xml file being setup with the following BLASR arguments: "-minMatch 8 -minPctIdentity 70 -bestn 1 -nCandidates 10 -maxScore -500 -nproc 1 -noSplitSubreads".

nucmer -t <threads> <genome1.fasta> <genome2.fasta>

dnadiff -d <out.delta>

**2. RNA-seq assembly and expression profiling**

java -jar trimmomatic-0.36.jar PE -threads <threads> <input.forward.fastq> <input.reverse.fastq> <output.forward_paired.fq.gz> <output.forward_unpaired.fq.gz> <output.reverse_paired.fq.gz> <output.reverse_unpaired.fq.gz> ILLUMINACLIP:TruSeq3-PE-2.fa:2:30:10

STAR --runThreadN <threads> --runMode genomeGenerate --genomeDir <path to genome fasta> --genomeFastaFiles <genome.fasta>

STAR --outSAMstrandField intronMotif --outSAMtype BAM SortedByCoordinate --twopassMode Basic --readFilesCommand zcat --runThreadN <threads> --genomeDir <path to genome fasta> --readFilesIn <forward_paired.fq.gz> <reverse_paired.fq.gz> --outFileNamePrefix <out prefix>

stringtie <in.bam> -p <threads> -o <out.gtf>

cufflinks2gff3 <in.gtf> > <out.gff3>

featureCounts -C -Q 10 -T <threads> -p -t exon -g gene_name -a <annotation.gtf> -o <out counts> <in.bam>

DESeq2 and PCA (R)

dds <- DESeqDataSetFromMatrix(countData=COUNTS, colData=META, design = ~Group)
dds <- DESeq(dds)
outputtable <- results(dds, contrast=c("Group", "Tissue", "Cavity"))

rld <- rlogTransformation(dds, blind=TRUE)
plotPCA (rld, intgroup=c("Group"), ntop=500)

**3. Nuclear genome annotation**

braker.pl --species=<output dir> --genome=<genome.fasta> --bam=<in.bam> --cores <threads> --alternatives-from-evidence=false --gff3 --softmasking

maker -fix_nucleotides -genome <genome.fasta> -base <base name> <maker_opts> <maker_bopts> <maker_exe>

gff3_merge -n -g -d <maker.output/master_datastore_index.log>

fasta_merge -d <maker.output/master_datastore_index.log>

interproscan.sh -appl PfamA -iprlookup -goterms -f tsv -i <proteins.fasta>

ipr_update_gff <max.gff> <proteins.fasta.tsv> > <max.functional_ipr.gff>

quality_filter.pl -s <max.functional_ipr.gff> > <standard.functional_ipr.gff>

maker_map_ids --prefix <locus tag> --suffix '' --iterate '' --justify 5 --sort_order <contig order> <standard.functional_ipr.gff> > <standard.functional_ipr.map>

map_gff_ids <standard.functional_ipr.map> <standard.functional_ipr.gff>

tRNAscan-SE -d -y -G -o <output name> <genome.fasta>

Rnammer -S euk -m tsu,ssu,lsu -gff <output gff> -h <output HMM report> <genome.fasta>

sma3s_v2.pl -i <proteins.fasta> -d <uniref90.fasta> -goslim -num_threads <threads>

/InterProScan/interproscan-5.28-67.0/interproscan.sh --disable-precalc -iprlookup -goterms -f tsv –tempdir <temp dir> -i <proteins.fasta> -o <out file>

phobius.pl -short <proteins.fasta>

BUSCO_v3.0/scripts/run_BUSCO.py -i <proteins.fasta> -o <out prefix> -m prot -l eukaryota_odb9 -c <threads>

**4. Mitochondrial genome assembly and phylogenetic analysis**

perl NOVOPlasty2.6.3.pl -c config.txt

canu -p <assembly-prefix> -d <assembly-directory> genomeSize=20k -pacbio-raw <pacbio_subreads.fasta> useGrid=false

bwa index <contig.fasta>

bwa mem -t <threads> <contig.fasta> <forward_paired.fq.gz> <reverse_paired.fq.gz> | samtools view -S -h -F 4 -o <out.sam> -

samtools sort -@ <threads> -o <sorted.bam> <in.sam>

samtools index <sorted.bam>

java -jar pilon-1.22.jar --genome <contig.fasta> --frags <sorted.bam> --outdir <output dir> --threads <threads>

CSA R <mito genomes fasta>

mafft --genafpair --maxiterate 1000 <rotated.fasta> > <mafft.fasta>

trimal -in <mafft.fasta> -out <mafft.trimal.fasta> -automated1

readal -in <mafft.trimal.fasta> -out <mafft.trimal.phy> -phylip

iqtree -s <mafft.trimal.phy> -pre <output_prefix> -alrt 10000 -ntmax <threads> -nt AUTO

**5. Repeat analysis**

xdformat -n -I <in.fasta>

RepeatModeler -engine wublast -database <in.fasta> -pa <threads>

RepeatClassifier -consensi <consensi.fa>

RepeatMasker -s -a -gff -nolow -xsmall -pa <threads> -lib <consensi.fa.classified> <in.fasta>

faToTwoBit -noMask <in.fasta> <fasta.2bit>

RepeatMasker/util/buildSummary.pl -genome <fasta.2bit> <in.fasta.out>

RepeatMasker/util/calcDivergenceFromAlign.pl -s <align.divsum> <in.fasta.align>

RepeatMasker/util/createRepeatLandscape.pl -twoBit <fasta.2bit> -div <align.divsum> > <out.html>

**6. Gene family evolution**

OrthoFinder-2.2.6_source/orthofinder/orthofinder.py -f <input directory>

python python_scripts/cafetutorial_clade_and_size_filter.py -i <unfiltered cafe input> -o <filtered cafe input> -s

CAFE-4.1/release/cafe

load -i <filtered cafe input> -t <threads> -l reports/log.txt tree <ultrametric tree>
lambda -l <lamda value> -t <tree>
report reports/output

perl PosiGene.pl -o=<out dir> -as=<anchor species> -tn=<threads> -rs=<reference species> -ts=<target species> -nhsbr=<non homologene species by reference>

**7. Diagnostic antigen search**

diamond blastx –db <path_to_db> -outfmt 101 -out <output_name> -q <path_to_query>

diamond blastp –db <path_to_db> -outfmt 101 -out <output_name> -q <path_to_query>
