# Supplementary material for: Comparative genomics and transcriptomics of 4 Paragonimus species provide insights into lung fluke parasitism and pathogenesis
Source: Gigascience. 2020 Jul 20;9(7):giaa073. doi: 10.1093/gigascience/giaa073 (PMC7370270; doi:10.1093/gigascience/giaa073)
Supplement: giaa073_Response_to_Reviewer_Comments_Original_Submission [file giaa073_response_to_reviewer_comments_original_submission.pdf]

Author's Response To Reviewer Comments

Close

Please see attached point-by-point response file (at the end of the PDF).

Close
