# Supplementary material for: Comparative genomics and transcriptomics of 4 Paragonimus species provide insights into lung fluke parasitism and pathogenesis
Source: Gigascience. 2020 Jul 20;9(7):giaa073. doi: 10.1093/gigascience/giaa073 (PMC7370270; doi:10.1093/gigascience/giaa073)
Supplement: giaa073_Response_to_Reviewer_Comments_Revision_1 [file giaa073_response_to_reviewer_comments_revision_1.pdf]

Author's Response To Reviewer Comments

Close

GIGA-D-19-00411R1

Point-by Point Response to editor and reviewers' comments

Editor:

We appreciate your email and your reasons for keeping this as a Research paper; however, we strongly suggest that this falls within our Data Note criteria - as the analysis you present is the validation that we ask for in a Data Note. So with this round of review, I'd like you to consider Reviewer #2's comments, as well as ours, and decide whether to change this into a Data Note or keep it as Research - removing the RNA-seq data for the specie, P. miyazakii.

Response:

We have completely removed the cavity vs tissue RNAseq analysis for the species, P. miyazakii. RNAseq analyses of the other species are presented in relevant sections. The key analyses include: i) validation of gene expression of all lung fluke-specific and conserved orthologous protein families (highly relevant analysis related to evolutionary adaptations in the genus Paragonimus), and ii) characterization of orthology among genes across this genus. The outcome confirmed consistent levels of expression of adult-stage genes supported by high Pearson correlations values.

Editor:

I would also like to point out that our Data Notes are indexed the same way as Research papers, and they are also half the cost of publishing a Research paper. If you choose to keep your paper as Research, we will however have to send it for a third round of review with an Editorial Board member.

Response:

With regard to the suggestion to reassign the manuscript to Data Note, we have re-reviewed the scope of both manuscript types. The 'Research Article' type is for "Manuscripts containing more detailed biological, medical or technical analyses of data" whereas Data Notes "focus on a particular dataset, and provide detailed methodology on data production, validation, and potential reuse." The rationale and findings of our manuscript are clearly within the ambit of the journal's Research Article, and indeed better fit there than in the Data Note section. By contrast, the Data Note type is intended "to incentivize and more rapidly release data before subsequent detailed analysis has been carried out." Specifically, this is because we first strategically choose four species spanning the genus Paragonimus to facilitate the presented analysis. Hence, in addition of presenting four novel genomes of neglected tropical disease pathogens, and transcriptomes for three of the species, we undertook detailed technical comparative analyses for four species of Paragonimus (lung flukes) and 17 other phylogenetically relevant species. The endeavor revealed key evolutionary genetic changes underlying diversification with the genus Paragonimus (e.g., gene family evolution and positive selection), which has provided novel biological knowledge about the tissue tropism of these medically important pathogens, which are among the most injurious food-borne helminths, infecting ~23 million people, with ~293 million people at risk for infection. Furthermore, the disease is frequently misdiagnosed as tuberculosis due to similar pulmonary symptoms (and maybe even for COVID-19), so there is an urgent need for the development of effective diagnostics. Because we strategically selected to sequence and analyze these 4 species that span the genus Paragonimus, we were able to match the new gene sets to previously identified single species-based Paragonimus diagnostic antigens, providing an opportunity to optimize and ensure consistent cross-reactivity for diagnostic assays, which is of a direct clinical. Thus, we have performed a thorough and extensive analysis of the available datasets for Paragonimus to provide insights of biological and clinical relevance. The manuscript provides far, far more than (to paraphrase the Data Note type) 'a rapid release of a novel dataset that we wish to incentivize'.

Reviewer 1:

The group answered all questions posted before and made the changes properly to make the manuscript more clear. Just two minor points:

1.1. Line 429 - maybe a typo. I believe that the author wanted to mention "Ncmer" instead of "Nucmerum";

Response: This has been fixed.

1.2. Line 394 - The authors reported well why they use PBJelly, but my point is that PBJELLY is not an assembler. " For P. kellicotti, PacBio were assembled using PBJelly". PBJelly, is a polishing tool to upgrade draft assemblies. Using it was correct, but the word assembler should be removed.

Response: This has been fixed.

Reviewer 2:

2.1. I thank the authors for the detailed response to my concerns. Regarding the RNA-Seq data, I appreciate that these samples are precious. However, that does not compensate for the lack biological replicates. If insufficient material cannot be obtained than the experiment should be considered. I am unable to support the strategy to consider the pleural and two peritoneal samples as replicates for the "cavities", nor lung and liver to be considered replicates for "tissues". Figure S4 doesn't provide strong support for this division; the pearson correlation values are nearly identical for peritoneal B vs peritoneal A (0.93), as peritoneal A vs lung (0.92). Even if one were to ignore this, there remains the problem that "tissues" has only two replicates. Further, single samples are used to make claims as to expression, for example in line 302 and table 4. My resolute stance on the shortcomings of this analysis, is because the work presented will be cited by others with confidence and may lead to a snow-balling of over-interpretation that can have significant and negative impact on research into these important parasites. Due to the problems I list, I recommend that the expression section is removed from the analysis.

Response: The RNAseq analysis of the cavities vs tissue of P. miyazakii has been removed.

2.2. Regarding the measures of completeness, I thank the authors for providing more details. I agree with their use of the eukaryotic set of conserved genes in BUSCO. I disagree with the claim that "fragmented" genes "may or may not be considered complete." I challenge the authors to provide published examples of this. The paper on the Heterohabditis genome does also use the eukaryote set, but does not claim fragmented genes are complete. The authors, in their response, offer S. mansoni's completeness of 73.8% as a comparison. In Wormbase-Parasite, all of the Schistosoma species have relative low scores on both CEGMA and BUSCO. It has been hypothesised that the blood flukes have lost a suite of genes previously thought to be highly conserved. Perhaps more impressive for the presented Paragonimus assemblies is the low proportion of duplicated complete genes. This suggests a low level of mis-assembly due to heterozygosity. I strongly encourage the authors to remove the fragmented genes from this "overall completeness" score in Table 1 and throughout the text.

Response: We have modified the R2 text along the lines suggested by the reviewer. We also removed the “overall completeness” category from the Table 1.

Close
