# Supplementary material for: Comparative genomics and transcriptomics of 4 Paragonimus species provide insights into lung fluke parasitism and pathogenesis
Source: Gigascience. 2020 Jul 20;9(7):giaa073. doi: 10.1093/gigascience/giaa073 (PMC7370270; doi:10.1093/gigascience/giaa073)
Supplement: giaa073_Response_to_Reviewer_Comments_Revision_2 [file giaa073_response_to_reviewer_comments_revision_2.pdf]

Author's Response To Reviewer Comments

Close

GIGA-D-19-00411R2

Response to editor comments

>Your manuscript "Comparative genomics and transcriptomics of four Paragonimus species provide insights into lung fluke parasitism and pathogenesis" (GIGA-D-19-00411R2) has been assessed by our Editorial Board member. Based on their feedback and my own assessment as Editor, I am pleased to inform you that it is potentially acceptable for publication in GigaScience, as a Research paper, once you have carried out some essential revisions suggested by myself.

>There are some sections missing that need to be added to the manuscript as well as a lot of key information in the supplementary files that should be moved to the main paper. - in particular under the "Availability of Supporting Data". Please see my comments in the attached PDF.

Author Response

Thank you for your provisional acceptance of the manuscript! As requested, we have made the following requested corrections to the manuscript, as indicated in the PDF:

- The keywords list has been moved down to the appropriate location following the Abstract.

- A “Data Description” section has been added, according to instructions.

- All supplementary figures have been moved into the manuscript as main figures, and the numbering has been fixed accordingly, both within the manuscript and on the system.

- Supplementary Table 1 was moved into the manuscript as a main table, and the table numbering has been fixed accordingly.

- However, due to the very large sizes of the remaining supplementary tables, it is impossible to provide these as tables within the text. These include:
• Supplementary Table S2 (now numbered S1) is a 6.2MB database, with 4 spreadsheets of >12,000 rows each, and up to 50 columns of data.
• Supplementary Table S3 (now numbered S2) is a 5.1 MB database, with 4 spreadsheets of ~5000 rows x ~75 columns.
• Supplementary Table S4 (now numbered as S3) is a 5.3MB database of ~20,000 rows x 36 columns.

We hope that it is acceptable to simply upload these as supplementary tables in order to make the data easily accessible to readers.

While not in your requested comment, we would also like to ask for your guidance in something that we are not able to do on the system. This MS has a co-shared first authorship and while this is correct in the MS file we could not find a way to make this distinction in the list of authors on the system. Can you please make sure this is filled out the correct way on the system.

Close
