# Supplementary material for: Comparative genomics and transcriptomics of 4 Paragonimus species provide insights into lung fluke parasitism and pathogenesis
Source: Gigascience. 2020 Jul 20;9(7):giaa073. doi: 10.1093/gigascience/giaa073 (PMC7370270; doi:10.1093/gigascience/giaa073)
Supplement: giaa073_Reviewer_1_Report_Original_Submission — Rodrigo Baptista, Ph.D. -- 1/19/2020 Reviewed [file giaa073_reviewer_1_report_original_submission.pdf]

Reviewer Report

Title: Comparative genomics and transcriptomics of four Paragonimus species provide insights into lung fluke parasitism and pathogenesis

Version: Original Submission **Date:** 1/19/2020

Reviewer name: Rodrigo Baptista, Ph.D.

Reviewer Comments to Author:

The manuscript entitled "Comparative genomics and transcriptomics of four Paragonimus species provide insights into lung fluke parasitism and pathogenesis" provides four new draft genomes for the genus, which are a great contribution for the field. Overall, the group did a great job in the whole paper, using mostly good method strategies and were very descriptive.
Here are some comments:
- The data description section seems to be a poor "method" section which seems redundant. The software names are missing (just their citations are shown) and is basically the same information provided in the methods section, which is well written. If any information on this section is important to be kept, I would fit this information in the methods section and delete the whole section.
- The Analysis section should be renamed to Results and Discussion
- Line 185-186 - the authors mentioned the draft genome sizes obtained and their respective completeness. Is this based in which expected complete genome size? Is there any complete genome of the genus complete (no gaps, physical evidence, telomere to telomere)? I understand that this is an estimation, but the authors should be careful and at least mention the expected "complete" genome size. Since we are talking about different species, these sizes should vary for each species.
- Line 205 - the group mentioned that some Orthologs vary in intron lengths and number of exons. Genomes that are highly repetitive (>50% repetitive) are usually very fragmented or have their most complex regions poorly assembled by short reads. Besides the group method using two libraries sizes for the Illumina applied using AllPaths, which I consider one of the best approaches for Illumina only assembly for this kind of complex genomes, there is a chance that these variations are due to problems in the assembly or frameshifts. Please provide how all these variations were validates to be real (Alignment support, etc).
- Line 422 - There is no need to mention the method in the Result and Discussion section. This was also observed in other lines in the Results section (eg. Line 291, 355,etc).
- Line 231-2 - How the identity was calculated? WGS or Orthologs? Amino-acid or Nucleotide level? This is really important when comparing identities between species, since assembly bias could be detected. This information should be added in the methods.
- Line 383-6 - I understand the idea of the group to give the organism-specific conserved orthologs for potential drug targeting, but when doing this I would recommend adding more information about these proteins, like localization, protein weight, TM and signal peptides, is that any hit in CHEMBL, etc. This would save time for the community that will read the paper to remove possible noise before starting to test the screening.

- Line 419 - Discussion should change to Conclusion
- Line 435-443 - Fresh Paragonimus (never frozen or stored for a long time) should be better for long sequencing, since there is less chance to have their DNA broken. This could affect differences in contiguity of some genomes.
- Line 446-454 - Why the group didn't try a hybrid approach for the assembly using long and short reads together? Why PBJELLY (usually reported to be used as gap filler tool for pacbio) was used for assembly of the long reads (CANU, HGAP and FALCON are much better), maybe it should be revised.
- Besides Pilon being a good choice for basecall polishing, I would recommend ICORN for the mitochondrial polishing. From my personal experience it usually corrects more regions than Pilon.
- There is no coverage obtained in the text about the sequencing datasets (Illumina/pacbio #X coverage). This is important to check how good was the basecall and polishing.

And here are some minor points:
- Line 113 - P. westermani is not in italic;
- Line 513 - change 3 for "three";
- Figures are in low resolution.

Methods

Are the methods appropriate to the aims of the study, are they well described, and are necessary controls included? Choose an item.

Conclusions

Are the conclusions adequately supported by the data shown? Choose an item.

Reporting Standards

Does the manuscript adhere to the journal's guidelines on [minimum standards of reporting?]() Choose an item.

Choose an item.

Statistics

Are you able to assess all statistics in the manuscript, including the appropriateness of statistical tests used? Choose an item.

Quality of Written English

Please indicate the quality of language in the manuscript: Choose an item.

Declaration of Competing Interests

Please complete a declaration of competing interests, considering the following questions:

- Have you in the past five years received reimbursements, fees, funding, or salary from an organisation that may in any way gain or lose financially from the publication of this manuscript, either now or in the future?
- Do you hold any stocks or shares in an organisation that may in any way gain or lose financially from the publication of this manuscript, either now or in the future?
- Do you hold or are you currently applying for any patents relating to the content of the manuscript?
- Have you received reimbursements, fees, funding, or salary from an organization that holds or has applied for patents relating to the content of the manuscript?
- Do you have any other financial competing interests?
- Do you have any non-financial competing interests in relation to this paper?

If you can answer no to all of the above, write 'I declare that I have no competing interests' below. If your reply is yes to any, please give details below.

I declare that I have no competing interests

I agree to the open peer review policy of the journal. I understand that my name will be included on my report to the authors and, if the manuscript is accepted for publication, my named report including any attachments I upload will be posted on the website along with the authors' responses. I agree for my report to be made available under an Open Access Creative Commons CC-BY license (http://creativecommons.org/licenses/by/4.0/). I understand that any comments which I do not wish to be included in my named report can be included as confidential comments to the editors, which will not be published.

Choose an item.

To further support our reviewers, we have joined with Publons, where you can gain additional credit to further highlight your hard work (see: https://publons.com/journal/530/gigascience). On publication of this paper, your review will be automatically added to Publons, you can then choose whether or not to claim your Publons credit. I understand this statement.

Yes Choose an item.
