# Supplementary material for: Comparative genomics and transcriptomics of 4 Paragonimus species provide insights into lung fluke parasitism and pathogenesis
Source: Gigascience. 2020 Jul 20;9(7):giaa073. doi: 10.1093/gigascience/giaa073 (PMC7370270; doi:10.1093/gigascience/giaa073)
Supplement: giaa073_Reviewer_2_Report_Original_Submission — James Wasmuth -- 2/3/2020 Reviewed [file giaa073_reviewer_2_report_original_submission.pdf]

Reviewer Report

Title: Comparative genomics and transcriptomics of four Paragonimus species provide insights into lung fluke parasitism and pathogenesis

Version: Original Submission **Date:** 2/3/2020

Reviewer name: James Wasmuth

Reviewer Comments to Author:

The submitted manuscript describes the sequencing, assembly, annotated and analysis of four species of the genus Paragonimus. The sequencing was predominantly Illumina short reads, with PacBio long reads generated for P. kellicotti. The authors conduct different gene family analyses, propose molecular components of host-parasite interactions, and identify proteins which are potential targets for vaccines or diagnostics. The authors also generate some RNA-Seq data for each species.

The generation and presentation of genomic assemblies for these four species will be useful in understanding their biology and developing new treatment. For the most part the manuscript is well written and easy to understand, for which the authors should be commended. However, I do have major concerns with the manuscript as presented.

**Major Concern 1: I tried to download much of the data to repeat the analyses but the speed of connection was slow. Therefore, I have looked into one section in more detail, the prediction of mimicry between Paragonimus proteins and their hosts. From lines 330 to 347, the authors describe orthologous genes (OGs) which are shared between at least one species of Paragonimus and their host to the exclusion of other trematodes (Figure 5D). The authors then speculate that these "may have evolved uniquely in lung flukes to mimic host factors[.]" Unfortunately, this is an artefact of sampling bias. I used BLAST to compare human STOX1, Zip67, and C5orf63 with Panagonimus, Schmidtea mediterranea and Caenorhabditis elegans proteins. For the first two, it is clear sequence similarity is similar or greater in S. mediterranea and C. elegans, raising reasonable doubt on specific mimicry between Paragonimus and human proteins. For C5orf63, the evalue of the alignment with a P. westermani protein was 0.041 and over only 40 amino acids. This suggests that it is an artifact of the clustering process in the OG generation.

```
blastp -outfmt 6 -max_hsps 1 -query STOX1.pep.fsa -db ../data/all.protein.fa | head -5
STOX1_HUMAN    F53B2.6 33.758  157    102    1    33    189    16    170    3.44e-28    120
STOX1_HUMAN    SMEST040264001  29.348  184    130    0    19    202    15    198    2.75e-22
103
STOX1_HUMAN    PKEL_11588    35.088  114    71    2    33    144    28    140    2.39e-13    71.6
STOX1_HUMAN    PMIY_01855    33.043  115    74    2    32    144    27    140    3.42e-12    72.0
STOX1_HUMAN    PWES_01040    33.628  113    72    2    34    144    29    140    1.20e-09    63.2
blastp -outfmt 6 -max_hsps 1 -query Zip67.pep.fsa -db ../data/all.protein.fa | head -6
ZN653_HUMAN    F45B8.4 33.918  171    106    4    442    612    101    264    7.44e-22    98.6
ZN653_HUMAN    SMEST004840001  44.048  84    47    0    496    579    211    294    1.50e-18
89.0
```

ZN653_HUMAN SMEST060422001 36.607 112 68 2 469 577 464 575 2.11e-18
91.7
ZN653_HUMAN SMEST058261001 35.484 155 92 5 460 614 77 223 1.63e-17
86.7
ZN653_HUMAN SMEST042630001 36.885 122 73 2 490 611 183 300 6.75e-17
84.3
ZN653_HUMAN PMIY_03311 46.988 83 44 0 496 578 200 282 7.00e-17 83.6
Query= YD286_HUMAN Glutaredoxin-like protein C5orf63 OS=Homo sapiens
OX=9606 GN=C5orf63 PE=2 SV=3
Length=138

```
                                          Score     E
Sequences producing significant alignments:          (Bits)   Value
PWES_06707                                33.5     0.041
>PWES_06707
Length=136
Score = 33.5 bits (75),  Expect = 0.041, Method: Compositional matrix adjust.
Identities = 17/43 (40%), Positives = 23/43 (53%), Gaps = 2/43 (5%)
Query  16  FGLFLRNCSASKTTLPVLTLFTKDPCPLCDEAKEVLKPYENRQ  58
             G ++   S +K  LP L +FTK  C LC  A   L+PY N+
Sbjct  26  LGQYISTISIAK--LPTLIVFTKPDCSLCKAAIVQLQPYVNKH  66
```

I recommend that the authors rethink their strategy for identifying molecular mimicry or remove the section entirely.

**Major Concern 2: The authors generated several RNA-Seq datasets for each species. Most of these were done single copies. Where replication was done, the authors note that it they are 'technical replicates', from which I understand that the samples are from the same biological source but run sequenced twice. These data are great for genome annotation, i.e. the identification of gene models. But, the accurate identification differentially expressed genes requires biological replicates. The authors' use of DESeq is not appropriate given the available data. Further, they should not be comparing FPKM as a statistically robust method to determine differential gene expression. Traditionally, people have asked for three biological replicates, though in depth modelling has shown that one needs to consider sequencing depth in addition to replication. I encourage the authors to read Schurch et al. https://www.ncbi.nlm.nih.gov/pubmed/27022035. I do appreciate that getting sufficient number of biological replicates in parasite systems is a challenge. However, this cannot justify having insufficient power in an analysis. Better not to conduct the analysis at all. I recommend that all references to differentially expressed genes is removed from the manuscript.

**Major Concern 3: The reported BUSCO scores are between 86% and 96% (Table 1). When comparing to parasite.wormbase, three of these Paragonimus assemblies would have the highest BUSCO score for any platyheliminth species and all are far above the best trematode, a reference quality assembly of S. mansoni. Further, in Table 1, the authors report a BUSCO score of 94.1% for P. westermani (India) previously sequenced (Oey et al.). However, Oey reports a BUSCO score of 65.3%. I ran BUSCO on P. westermani (Japan) using the eukayota orthologue set (-l eukaryota_odb9) and got "C:77.9%[S:76.9%,D:1.0%],F:8.9%,M:13.2%,n:303". I presume that the authors used a different

orthologue set for the "--lineage", but they do not state which one. Please can the authors provide further clarification.
**Major Concern 4: On reviewing the methods, I could not find sufficient detail to rerun many of the analyses properly. I recommend that the authors provide a file with all the commands, options and software versions. This file serves two purposes. The first is so that replication of the work will support its robustness. The second is so that other researchers can implement these methods for their own species of interest.

Methods

Are the methods appropriate to the aims of the study, are they well described, and are necessary controls included? Choose an item.

Conclusions

Are the conclusions adequately supported by the data shown? Choose an item.

Reporting Standards

Does the manuscript adhere to the journal's guidelines on minimum standards of reporting? Choose an item.

Choose an item.

Statistics

Are you able to assess all statistics in the manuscript, including the appropriateness of statistical tests used? Choose an item.

Quality of Written English

Please indicate the quality of language in the manuscript: Choose an item.

Declaration of Competing Interests

Please complete a declaration of competing interests, considering the following questions:

- Have you in the past five years received reimbursements, fees, funding, or salary from an organisation that may in any way gain or lose financially from the publication of this manuscript, either now or in the future?
- Do you hold any stocks or shares in an organisation that may in any way gain or lose financially from the publication of this manuscript, either now or in the future?
- Do you hold or are you currently applying for any patents relating to the content of the manuscript?

- Have you received reimbursements, fees, funding, or salary from an organization that holds or has applied for patents relating to the content of the manuscript?
- Do you have any other financial competing interests?
- Do you have any non-financial competing interests in relation to this paper?

If you can answer no to all of the above, write 'I declare that I have no competing interests' below. If your reply is yes to any, please give details below.

I declare that I have no competing interests

I agree to the open peer review policy of the journal. I understand that my name will be included on my report to the authors and, if the manuscript is accepted for publication, my named report including any attachments I upload will be posted on the website along with the authors' responses. I agree for my report to be made available under an Open Access Creative Commons CC-BY license (http://creativecommons.org/licenses/by/4.0/). I understand that any comments which I do not wish to be included in my named report can be included as confidential comments to the editors, which will not be published.

Choose an item.

To further support our reviewers, we have joined with Publons, where you can gain additional credit to further highlight your hard work (see: https://publons.com/journal/530/gigascience). On publication of this paper, your review will be automatically added to Publons, you can then choose whether or not to claim your Publons credit. I understand this statement.

Yes Choose an item.
