# Supplementary material for: Comparative genomics and transcriptomics of 4 Paragonimus species provide insights into lung fluke parasitism and pathogenesis
Source: Gigascience. 2020 Jul 20;9(7):giaa073. doi: 10.1093/gigascience/giaa073 (PMC7370270; doi:10.1093/gigascience/giaa073)
Supplement: giaa073_Reviewer_2_Report_Revision_1 — James Wasmuth -- 4/21/2020 Reviewed [file giaa073_reviewer_2_report_revision_1.pdf]

Reviewer Report

Title: Comparative genomics and transcriptomics of four Paragonimus species provide insights into lung fluke parasitism and pathogenesis

Version: Revision 1 **Date:** 4/21/2020

Reviewer name: James Wasmuth

Reviewer Comments to Author:

I thank the authors for the detailed response to my concerns.
Regarding the RNA-Seq data, I appreciate that these samples are precious. However, that does not compensate for the lack biological replicates. If insufficient material cannot be obtained than the experiment should be considered. I am unable to support the strategy to consider the pleural and two peritoneal samples as replicates for the "cavities", nor lung and liver to be considered replicates for "tissues". Figure S4 doesn't provide strong support for this division; the pearson correlation values are nearly identical for peritoneal B vs peritoneal A (0.93), as peritoneal A vs lung (0.92). Even if one were to ignore this, there remains the problem that "tissues" has only two replicates. Further, single samples are used to make claims as to expression, for example in line 302 and table 4. My resolute stance on the shortcomings of this analysis, is because the work presented will be cited by others with confidence and may lead to a snow-balling of over-interpretation that can have significant and negative impact on research into these important parasites. Due to the problems I list, I recommend that the expression section is removed from the analysis.
Regarding the measures of completeness, I thank the authors for providing more details. I agree with their use of the eukaryotic set of conserved genes in BUSCO. I disagree with the claim that "fragmented" genes "may or may not be considered complete." I challenge the authors to provide published examples of this. The paper on the Heterohabditis genome does also use the eukaryote set, but does not claim fragmented genes are complete. The authors, in their response, offer S. mansoni's completeness of 73.8% as a comparison. In Wormbase-Parasite, all of the Schistosoma species have relative low scores on both CEGMA and BUSCO. It has been hypothesised that the blood flukes have lost a suite of genes previously thought to be highly conserved. Perhaps more impressive for the presented Paragonimus assemblies is the low proportion of duplicated complete genes. This suggests a low level of mis-assembly due to heterozygosity. I strongly encourage the authors to remove the fragmented genes from this "overall completeness" score in Table 1 and throughout the text.

Methods

Are the methods appropriate to the aims of the study, are they well described, and are necessary controls included? Choose an item.

Conclusions

Are the conclusions adequately supported by the data shown? Choose an item.

Reporting Standards

Does the manuscript adhere to the journal's guidelines on minimum standards of reporting? Choose an item.

Choose an item.

Statistics

Are you able to assess all statistics in the manuscript, including the appropriateness of statistical tests used? Choose an item.

Quality of Written English

Please indicate the quality of language in the manuscript: Choose an item.

Declaration of Competing Interests

Please complete a declaration of competing interests, considering the following questions:

- Have you in the past five years received reimbursements, fees, funding, or salary from an organisation that may in any way gain or lose financially from the publication of this manuscript, either now or in the future?
- Do you hold any stocks or shares in an organisation that may in any way gain or lose financially from the publication of this manuscript, either now or in the future?
- Do you hold or are you currently applying for any patents relating to the content of the manuscript?
- Have you received reimbursements, fees, funding, or salary from an organization that holds or has applied for patents relating to the content of the manuscript?
- Do you have any other financial competing interests?
- Do you have any non-financial competing interests in relation to this paper?

If you can answer no to all of the above, write 'I declare that I have no competing interests' below. If your reply is yes to any, please give details below.

I declare that I have no competing interests.

I agree to the open peer review policy of the journal. I understand that my name will be included on my report to the authors and, if the manuscript is accepted for publication, my named report including any attachments I upload will be posted on the website along with the authors' responses. I agree for my report to be made available under an Open Access Creative Commons CC-BY license (http://creativecommons.org/licenses/by/4.0/). I understand that any comments which I do not wish to be included in my named report can be included as confidential comments to the editors, which will not be published.

Choose an item.

To further support our reviewers, we have joined with Publons, where you can gain additional credit to further highlight your hard work (see: https://publons.com/journal/530/gigascience). On publication of this paper, your review will be automatically added to Publons, you can then choose whether or not to claim your Publons credit. I understand this statement.

Yes Choose an item.
